# Spatiotemporal heterologous vaccination relies on type I interferons at priming to enhance mucosal immunity

**DOI:** 10.1038/s44321-026-00539-w

**Published:** 2026-10-12

**Authors:** Matthew J Wiest, Gabriel Laghlali, Julianne d’Acunzo, Chinghan Chung, Dilara Karadag, Vivian Yan, Seok-Chan Park, Moataz Noureddine, Katarzyna W Janczak, Chun Chen, Mohammad Farazuddin, Jessica J O’Konek, Adolfo García-Sastre, James R Baker, Michael Schotsaert, Pamela T Wong

**Affiliations:** 1 Michigan Nanotechnology Institute for Medicine and Biological Sciences, University of Michigan Medical School, Ann Arbor, MI USA; 2 Department of Internal Medicine, University of Michigan Medical School, Ann Arbor, MI USA; 3 Department of Microbiology, Icahn School of Medicine at Mount Sinai, New York, NY USA; 4 Global Health and Emerging Pathogens Institute, Icahn School of Medicine at Mount Sinai, New York, NY USA; 5 Department of Pharmaceutical Sciences, Ghent University, Ghent, Belgium; 6 Graduate School of Biomedical Sciences, Icahn School of Medicine at Mount Sinai, New York, NY USA; 7 Icahn Genomics Institute, Icahn School of Medicine at Mount Sinai, New York, NY USA; 8 Department of Medicine, Division of Infectious Diseases, Icahn School of Medicine at Mount Sinai, New York, NY USA; 9 Department of Pathology, Molecular and Cell-Based Medicine, Icahn School of Medicine at Mount Sinai, New York, NY USA; 10 Tisch Cancer Institute, Icahn School of Medicine at Mount Sinai, New York, NY USA; 11 Marc and Jennifer Lipschultz Institute for Precision Immunology, Icahn School of Medicine at Mount Sinai, New York, NY USA; 12 Department of Immunology and Immunotherapy, Icahn School of Medicine at Mount Sinai, New York, NY USA; 13 Michigan Center for Infectious Disease Threats and Pandemic Preparedness, University of Michigan, Ann Arbor, MI USA; 14 Department of Biomedical Engineering, University of Michigan, Ann Arbor, MI USA

**Keywords:** Immunology, Microbiology, Virology & Host Pathogen Interaction

## Abstract

Parenteral vaccines against respiratory viruses poorly block infection and transmission due to suboptimal induction of mucosal immunity. In contrast, hybrid immunity and heterologous-route immunization regimens generate protective mucosal and systemic immunity. Herein, we examine how chronological order in heterologous prime/boost immunization regimens impacts immune responses using combinations of intramuscular mRNA and intranasal adjuvanted subunit vaccines against SARS-CoV-2. Enhanced long-lived mucosal and systemic immunity depended strongly on vaccine delivery sequence, with heterologous regimens administered in alternate order of routes yielding markedly distinct immune profiles with different Th-biases and divergent localizations. Immunity imparted through heterologous immunization was more durable and responsive to delayed boosting than parenteral-only regimens. Furthermore, intramuscular mRNA immunization uniquely shaped gene expression locally and systemically through IFN-I induction, allowing for enhanced responses to subsequent mucosal immunizations. These findings demonstrate that the context of initial antigen exposure—including the route of exposure and associated inflammatory signals—profoundly shapes responses to subsequent encounters. Thus, vaccine responses in antigen-experienced hosts differ fundamentally from those induced on a naive baseline, reflecting the influence of pre-existing innate and adaptive immune memory.

## Synopsis

Sequential heterologous-route immunization using a rationally designed mucosal adjuvant enhances systemic and mucosal immunity compared with parenteral mRNA-only vaccination, highlighting the role of pre-established immunity in shaping subsequent vaccination responses.

- Vaccine type and priming route imprint T cell Th bias, dictate mucosal TRM and sIgA induction, and skew systemic antibody class switching.
- Type I IFN induction at priming conditions responses to subsequent mucosal immunization, enhancing mucosal cellular and humoral immunity.
- Heterologous route prime/boost immunization enhances the longevity ( > 3mo) of systemic and mucosal responses compared with homologous immunization.
- Robust mucosal immunity established through heterologous route immunization can be boosted after a prolonged interval ( > 3 mo) through either mucosal or parenteral routes.
- The same regimen can elicit distinct immune outcomes when administered in different chronological orders, highlighting the role of pre-existing immunity in shaping subsequent responses.

## Abstract (web summary)

Sequential heterologous-route immunization using a rationally designed mucosal adjuvant enhances systemic and mucosal immunity compared with parenteral mRNA-only vaccination, highlighting the role of pre-established immunity in shaping subsequent vaccination responses.

## Graphical abstract

**Figure:**
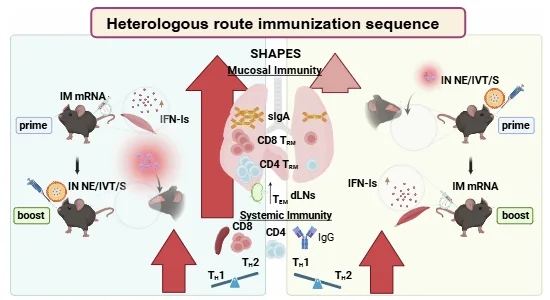

The paper explained **Problem**

Intramuscular mRNA vaccines effectively prevent severe disease, but limited durability and suboptimal induction of mucosal immunity constrain their efficacy in preventing infection and viral transmission. Hybrid immunity, vaccination plus infection, generates improved and durable respiratory mucosal immunity, motivating heterologous prime-boost strategies combining distinct routes of administration. However, the development of mucosal adjuvants that are both safe and capable of eliciting optimal systemic and mucosal antiviral responses remains challenging. Moreover, significant gaps remain in our understanding of how the chronological sequence of antigen exposure—including vaccine modality, route, and infection history—shapes the overall immune outcome. Defining how pre-existing immune landscapes can be strategically harnessed to guide subsequent immune responses will facilitate the development of more effective vaccination strategies.

**Results**

Herein, we developed a safe, rationally designed intranasal adjuvant consisting of a nanoemulsion with an RNA-based RIG-I agonist (NE/IVT). We demonstrate that NE/IVT used as a mucosal subunit booster following parenteral mRNA vaccination drives markedly enhanced, long-lived protective systemic and mucosal immune responses against SARS-CoV-2. Strikingly, the order of immunization in these heterologous regimens had a profound impact not only on the ability to induce long-lived mucosal immune responses but also shifted the polarization of the response (T_H_1/T_H_2). In other words, administering the same vaccines in reverse sequential order significantly alters the induced immune profile. Our findings indicate that this sequence dependence is due, at least in part, to innate immune signals induced specifically during priming that condition the host for enhanced responsiveness to subsequent mucosal vaccination.

**Impact**

This study demonstrates that pre-existing immune baselines, established during initial antigen exposure, critically shape downstream responses to subsequent vaccination, particularly at mucosal surfaces. By defining how sequence and route of immune exposure influence response magnitude, durability, and polarization, these findings provide mechanistic insight into how vaccination regimens can be strategically tailored to elicit more optimal systemic and mucosal immunity. This work informs the development of next-generation vaccination strategies that can maximize durable protection and has broader implications for understanding how mucosal infections shape later immune responses.

## Introduction

Compared to subunit and inactivated virus vaccines, the intramuscularly (IM)-administered mRNA-lipid nanoparticle (LNP) vaccines (ex. COMIRNATY, Spikevax) provide significant developmental advantages (Hogan and Pardi, 2022; Uddin and Roni, 2021) and are highly effective at preventing severe disease (Collier et al, 2021). However, lower durability of induced immune responses has now been documented across multiple mRNA vaccines against different pathogens (Collier et al, 2021; Andreoni et al, 2024). Furthermore, parenterally administered mRNA-LNP vaccines induce suboptimal mucosal immunity (Tang et al, 2022; Pieren et al, 2023; McMahan et al, 2024), making them less effective at preventing infection, lowering viral carriage, and blocking viral transmission (Roederer et al, 2024). These limitations have been accentuated as SARS-CoV-2 variants capable of immune escape continue to evolve and as targets of new mRNA vaccines expand to other respiratory viruses—especially those of pandemic potential such as influenza viruses, highlighting the need for improved strategies to induce durable and broad humoral, cellular, and mucosal immunity.

Lack of induction of protective mucosal immunity is a shortcoming common to all parenterally administered respiratory virus vaccines (Baker et al, 2022; Cokarić Brdovčak et al, 2022). While mucosal vaccines effectively generate mucosal immunity, these vaccines have required the addition of adjuvants or the use of replication-competent vaccines to generate effective immune responses, limiting their use to select populations (Lavelle and Ward, 2022). We previously demonstrated the safety and efficacy of our rationally designed combination intranasal (IN) adjuvant consisting of a nanoemulsion (NE) with an RNA-based RIG-I agonist (IVT) (Martínez-Gil et al, 2013) for mucosal delivery and immune potentiation of subunit vaccines toward SARS-CoV-2 spike (S) protein and inactivated influenza virus (Jangra et al, 2021, 2023; Wong et al, 2021). NE/IVT activates multiple pathogen recognition receptors, mimicking the viral innate immune activation events that occur during natural infection, induces robust mucosal immunity, and skews adaptive responses towards an optimal Th1/Th17 profile (Bielinska et al, 2014; Wong et al, 2021; Jangra et al, 2021).

With multiple licensed COVID-19 vaccines and large portions of the population infected pre- and/or post-immunization, a significant amount of data on vaccination with heterologous vaccine types and “hybrid immunity” (immunity obtained through vaccination plus infection) has been obtained. Characterizations of immune breadth and durability have shown the benefit of heterologous immunizations with regard to vaccine modalities in clinical studies, and to route (IM vs. IN) in preclinical models (Barros-Martins et al, 2021; Tan et al, 2022; Jara et al, 2022). However, the greatest enhancement has been shown, particularly with regard to mucosal immunity, in individuals with hybrid immunity (Bobrovitz et al, 2023). Key distinctions of natural viral infection include the route of antigen exposure (respiratory mucosa), activation of an array of antiviral-specific innate immune pathways, and robust antigen generation. It has become clear that the site of antigen presentation (mucosal vs. systemic) highly influences the resultant immune response due to the distinct immune environments of these compartments and their uniquely associated innate immune activation milieus. Despite this, much remains to be defined regarding how repeated antigen exposures through heterologous modalities and routes (infection, mucosal or systemic immunization) shape the resultant antibody response and memory T cell pool.

In hybrid immunity, the initial prime encounter (immunization or infection) biases both humoral (Voss et al, 2024) and cellular immune responses (Gray-Gaillard et al, 2024). For example, it has been shown to affect the cytotoxic and proliferative capacity of memory CD4^+^ T cells (Gray-Gaillard et al, 2024). It is therefore likely that in heterologous immunization, the route of initial antigen encounter along with the inflammatory milieu will be consequential in imprinting how immune responses are shaped by later boost immunizations. We herein assessed how modulating the spatiotemporal delivery of parenteral and mucosal vaccines in heterologous sequences can be used to guide the induced mucosal, humoral, and cellular immune profiles in a SARS-CoV-2 vaccination mouse model. To emulate hybrid immunity, we developed heterologous vaccination strategies utilizing IM mRNA vaccines to induce systemic immunity and a mucosal subunit vaccine containing our NE/IVT adjuvant to direct immune responses to the mucosa (Laghlali et al, 2024; Slamanig et al, 2024; Mao et al, 2022). Our results show that both the vaccine type and route of administration of the prime immunization play important roles in the ability of subsequent booster immunizations to establish mucosal immunity and have a dominant role in imprinting distinct Th biases. We demonstrate that this is partially explained by IM mRNA priming immunization establishing a strong type I interferon (IFN-I) response both locally within the muscle tissue and distally within the nasal mucosa, allowing for possible synergism with subsequent mucosal boosting, while mucosal IN NE/IVT/S priming effects are primarily localized to the nasal mucosa. These results demonstrate that even with the same overall administered vaccine components, how one arrives at the destination (chronological sequence of administration) is critically important. Moreover, we demonstrate that heterologous vaccination regimens with IM mRNA prime followed by IN NE/IVT adjuvanted S protein boost impart markedly more durable immune profiles than homologous vaccination and were most amenable to delayed boosting even months after the initial immunization series. Furthermore, despite waning immunity with homologous mRNA immunization, a single NE/IVT adjuvanted mucosal boost was able to successfully impart optimal mucosal cellular immunity as well as restore systemic immunity. These results strongly demonstrate that incorporation of mucosal boosters with an adjuvant triggering innate responses mirroring those during viral infection after a parenteral vaccination series can impart more durable and comprehensive protective immune responses to respiratory pathogens. Importantly, these findings have significant implications for designing strategic vaccination regimens that most effectively shape and redirect pre-existing immune responses.

## Results

### Prime immunization modality and delivery route shape antibody class switching and establishment of mucosal immunity in heterologous route immunization

**Figure 1:**
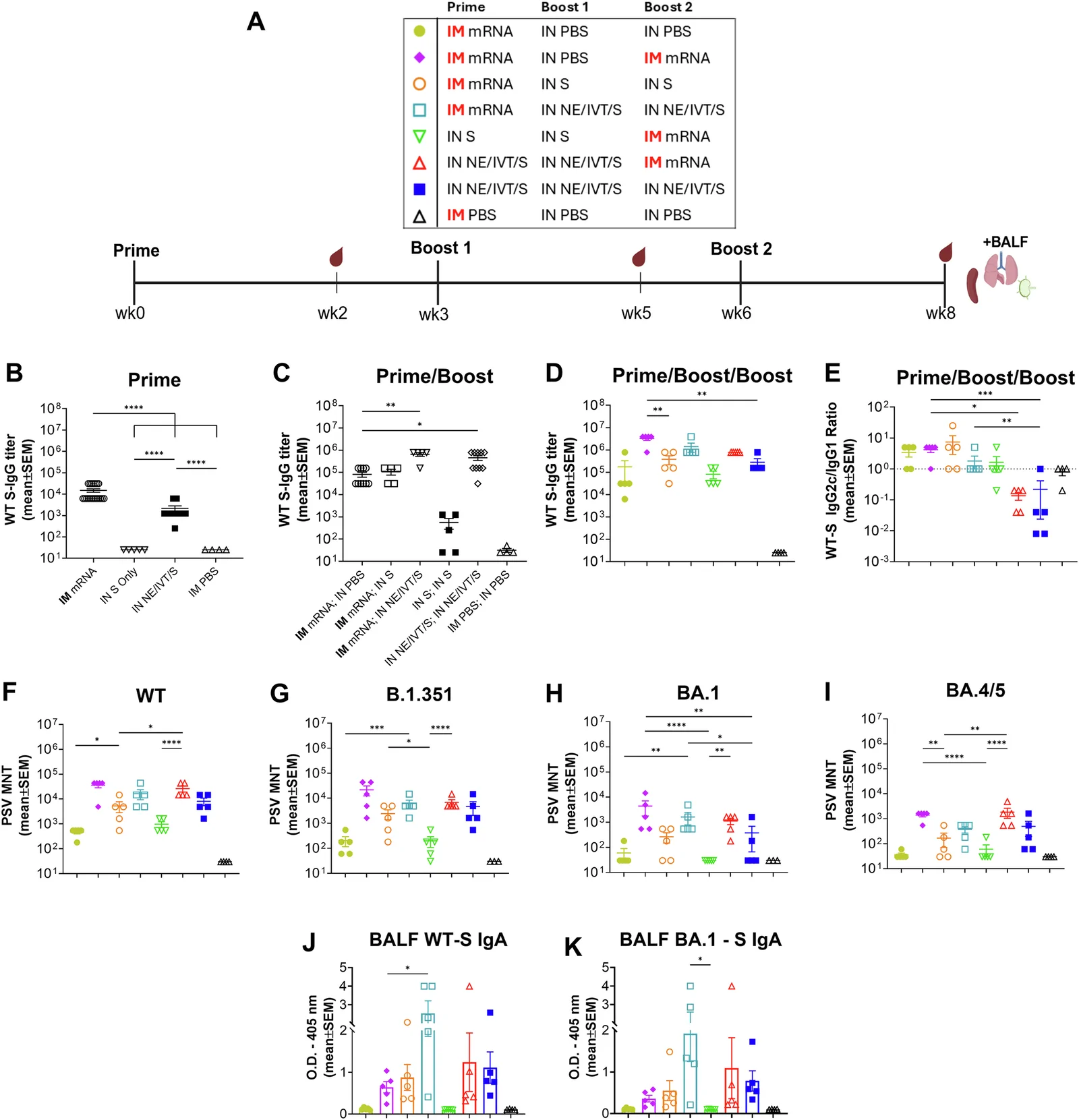
In heterologous immunization, the prime immunization route and immunogenicity shape antibody class switch dominance and mucosal IgA production. (**A**) C57BL/6J mice were immunized with a prime/boost/boost schedule at 3-week intervals. Humoral and cellular immune profiling was performed at week 8. Mice were separated based on prime/boost/boost grouping designation as the study progressed. Serum levels of WT-S IgG measured 2 weeks (**B**) post-prime, (**C**) post-prime/boost, and (**D**) post-prime/boost/boost immunization determined by ELISA. (**E**) Serum WT-S IgG2c/IgG1 ratios post-prime/boost/boost determined by ELISA. Serum neutralizing antibody titers post-prime/boost/boost to (**F**) WT, (**G**) B.1.351, (**H**) BA.1, and (**I**) BA.4/BA.5 variant PSVs as measured by microneutralization assay. (**J**) WT and (**K**) BA.1 S-specific IgA in BALF after prime/boost/boost immunization determined by ELISA. (*n* = 5/immunized groups, *n* = 3–4/group PBS controls) Graphs representative of mean ± SEM with titers log10-transformed prior to statistical analysis, with group differences assessed by one-way ANOVA with post-hoc Tukey’s multiple comparison test. *P* < 0.05, **P < 0.01, ***P < 0.001, ****P < 0.0001 shown for select groups (full statistical analysis with exact *P* values provided in Dataset EV1). Source data are available online for this figure.

To investigate the impact of the prime immunization on shaping responses to subsequent booster immunizations and determine whether the chronological sequence of repeated antigen exposures through heterologous routes (mucosal vs. parenteral) influences the final immune responses, mice were immunized with various heterologous prime/boost regimens at 3 week intervals, consisting of IM mRNA or IN S protein-based vaccines (see Fig. 1A for study design and abbreviations). A prime/boost/boost regimen was employed in which IN immunizations were performed with the S protein of Wuhan Hu-1 SARS-CoV-2 (WT S) (20 μg) with/without NE/IVT adjuvant, and IM immunizations were performed with the BNT162b2 mRNA vaccine (mRNA) (1.7 μg). To examine the influence of parenteral priming, mice were primed with IM mRNA and subsequently boosted twice with either IN S or IN NE/IVT/S, as the NE/IVT adjuvant requires two IN immunizations to induce optimal mucosal responses (Jangra et al, 2021). To examine the impact of mucosal priming, mice were primed and boosted with IN S or IN NE/IVT/S, followed by a final boost with IM mRNA. Comparator homologous regimens included prime/boost/boost immunizations with IN NE/IVT/S, and prime/boost immunizations with IM mRNA.

Post-prime (Fig. 1B), both IM mRNA and IN NE/IVT/S induced high levels of serum WT-S-specific IgG, while unadjuvanted IN S did not induce detectable IgG. Boosting IM mRNA primed mice with IN NE/IVT/S significantly enhanced serum S-specific IgG, whereas boosting with unadjuvanted IN S did not significantly enhance IgG compared to a single IM mRNA immunization (Fig. 1C). Homologous IN NE/IVT/S prime/boost immunization also induced robust IgG levels comparable to those induced by the heterologous IM mRNA; IN NE/IVT/S regimen. After the third immunization (prime/boost/boost), total S-specific IgG was further enhanced to a comparable level between groups receiving an IM mRNA immunization with two IN NE/IVT/S immunizations regardless of chronological sequence (IM mRNA; IN NE/IVT/S; IN NE/IVT/S vs. IN NE/IVT/S; IN NE/IVT/S; IM mRNA) (Fig. 1D). These groups produced slightly higher IgG compared to the group given homologous IN NE/IVT/S prime/boost/boost (hereafter referred to as homologous IN NE/IVT/S) and induced comparable titers as the homologous IM mRNA prime/boost group (hereafter referred to as homologous IM mRNA). Heterologous regimens with IM mRNA and unadjuvanted IN S resulted in significantly lower titers than those with IN NE/IVT adjuvant. However, mucosal boosting after IM mRNA priming enhanced WT-S IgG titers in comparison to IN S priming followed by IM mRNA boosting, emphasizing the role of sequence in shaping antibody responses.

Post-prime/boost/boost, subclass analysis revealed that all groups induced high levels of S-specific IgG1 reflecting the same relative pattern as total IgG between groups (Appendix Fig. S1A). However, IgG2c was much higher with homologous IM mRNA and heterologous IM mRNA; IN NE/IVT/S; IN NE/IVT/S (hereafter referred to as IM mRNA →IN NE/IVT/S) regimens than with homologous IN NE/IVT/S and IN NE/IVT/S; IN NE/IVT/S; IM mRNA (hereafter referred to as IN NE/IVT/S→IM mRNA) (Appendix Fig. S1B). The impact of chronological sequence of administration in heterologous immunization was especially highlighted by the IgG2c/IgG1 ratio, with groups primed IN showing substantially lower ratios than those primed with IM mRNA, suggesting a durable influence of the prime immunization type/route on skewing of responses to booster immunization (Fig. 1E).

We next examined the breadth of induced viral neutralizing antibodies (nAbs) utilizing pseudovirus (PSV) reporter viruses expressing the S proteins from the WT, B.1.351, BA.1, and BA.4/5 variants (Fig. 1F–I). Homologous IM mRNA immunization, and heterologous prime/boost/boost with IM mRNA and adjuvanted IN NE/IVT/S (regardless of sequence) all induced equivalent and robust titers across divergent variants. Intriguingly, in heterologous regimens with unadjuvanted IN S, nAb enhancement was strongly sequence dependent, with enhanced titers over a single mRNA dose only observed for the IM mRNA prime followed by IN S boosting. Furthermore, this improvement was only observed for the WT and less divergent B.1.351 PSVs, suggesting limited breadth of neutralization without the NE/IVT adjuvant. The homologous IN NE/IVT/S group demonstrated equivalent nAb titers against WT, BA.1.351, and BA.4/5 as the heterologous IM mRNA and IN NE/IVT/S regimens; however, these titers were notably reduced against BA.1, suggesting possible variations in epitope specificity.

As mucosal secretory IgA (sIgA) plays a major role in preventing infection and transmission, we quantified antigen-specific IgA in bronchoalveolar lavage fluid (BALF) post-prime/boost/boost. Heterologous IM mRNA→IN NE/IVT/S immunization resulted in a dramatic increase in BALF WT and BA.1 S-specific IgA, with similar reactivity towards both variants (Fig. 1J,K). Interestingly, while homologous IN NE/IVT/S induced higher IgA than homologous IM mRNA, heterologous prime/pull immunization (IM mRNA→IN NE/IVT/S) enhanced IgA induction to levels exceeding an additive effect of the homologous IN or IM regimens, pointing to a synergistic effect with the IM/IN approach. Importantly, this IgA enhancement was not observed with the reverse mucosal prime, parenteral boost (IN NE/IVT/S→IM mRNA) regimen, which induced levels of IgA similar to homologous IN NE/IVT/S. While boosting IM mRNA primed mice with unadjuvanted IN S gave modest IgA enhancement relative to the single IM mRNA immunization, no IgA was detected in mice given the reverse IN S→IM mRNA sequence. These results demonstrate the importance of adjuvanted mucosal immunization in driving optimal IgA induction and highlight the advantages of mucosal immunizations used as a booster to pull (and bolster) immune responses primed systemically to mucosal surfaces. Conversely, systemic boosting did not boost mucosally primed mucosal antibody responses. Thus, the vaccine-induced inflammatory signals and localization of priming signals with the first antigen exposure have a major role in shaping the immune responses to subsequent booster immunizations and ultimately in shaping optimal vaccination outcomes in heterologous immunization regimens.

### Priming route in heterologous immunization shapes the formation of tissue-resident memory T cells in the nasal mucosa and draining lymph nodes

In the absence of robust nAbs, which wane quickly after IM mRNA immunization (Evans et al, 2022), protection from viral infection is afforded by both CD4^+^ and CD8^+^ T cells (Montoya et al, 2024). Furthermore, as T cell epitopes are more conserved, they can provide durable protection as variants continue to emerge. Tissue-resident memory T cells (T_RM_s) in particular are important mediators in both protection and blocking viral transmission (Hassert and Harty, 2022). Thus, robust induction of both systemic effector memory (T_EM_s) T cells and respiratory T_RM_s through vaccination is critical to fortifying protection. While the role of mucosal immunization in heterologous prime/pull immunization in promoting T_RM_ induction has been demonstrated (Longet and Paul, 2023; Mao et al, 2022; Hassan et al, 2020), how the chronological sequence of route of antigen exposure in heterologous regimens shapes vaccine-induced cellular immune responses, especially within mucosal tissues, is not well defined. We therefore assessed how the vaccination sequence and the choice in priming route in heterologous immunization regimens impact the generation of CD4^+^/CD8^+^ S-specific CD69^+^ T_EM_s and T_RM_s within the systemic (spleen) and mucosal (mucosal draining lymph nodes (dLNs), lung, nasal-associated lymphoid tissue (NALT) immune compartments. Due to the suboptimal immunogenicity of unadjuvanted IN S, we focused our characterizations on NE/IVT adjuvanted IN immunization regimens.

**Figure 2:**
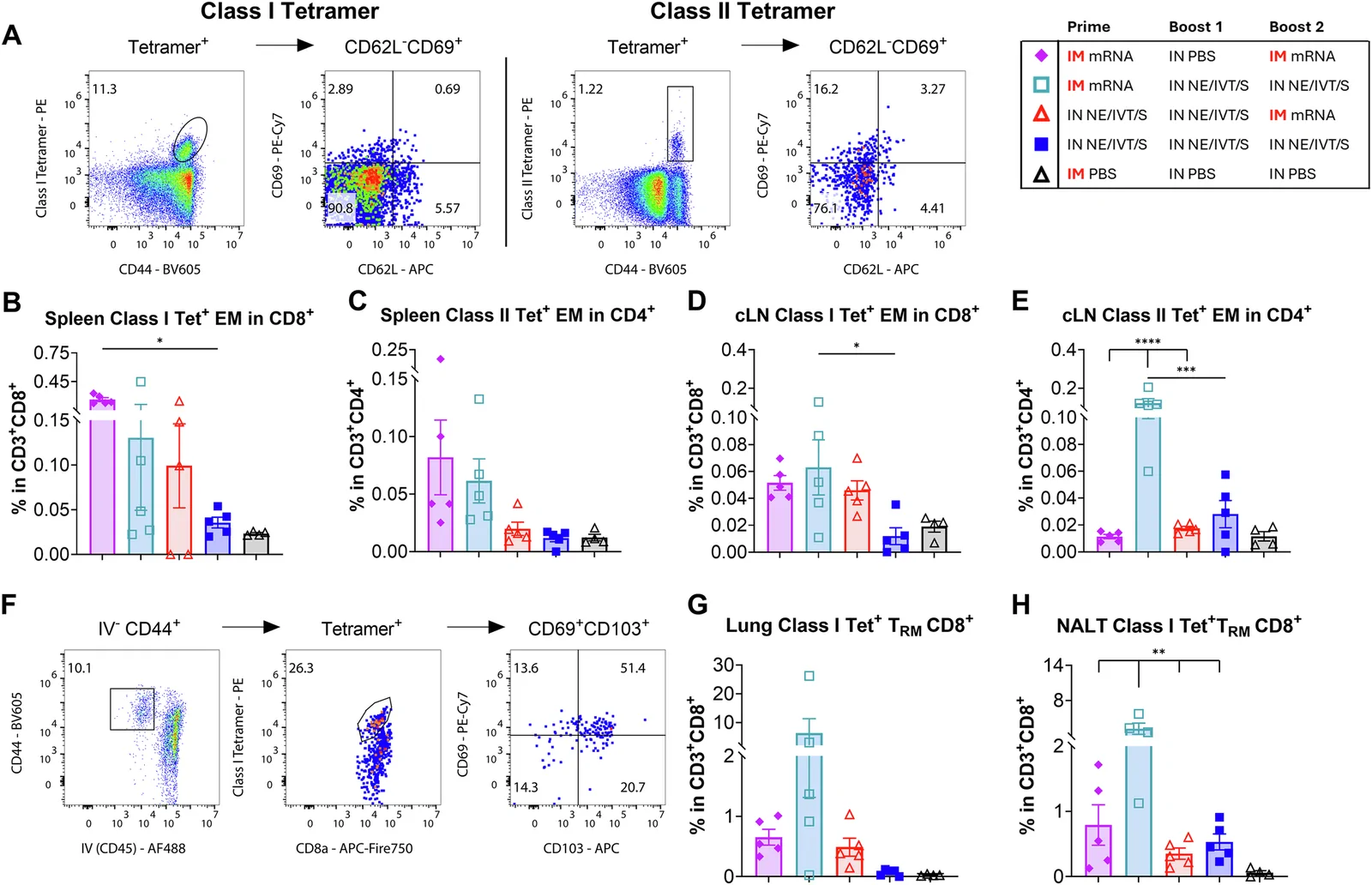
Antigen-specific T cells in the respiratory mucosa are fostered by heterologous immunization with systemic priming and mucosal boosting but not by the reverse. Single cell suspensions from the spleens, cLNs, lungs, and NALTs of immunized C57BL/6J mice were isolated 2 weeks post prime/boost/boost. (**A**) Representative gating pathway for CD8^+^Class I Tetramer^+^ and CD4^+^Class II Tetramer^+^ T cells in spleen and cLN. Frequencies of S-specific (**B**, **D**) effector memory (CD3^+^CD8^+^CD44^+^Class I Tet^+^ CD62L^-^) CD8^+^ T cells and (**C**, **E**) effector memory (CD3^+^CD4^+^CD44^+^Class II Tet^+^ CD62L^-^) CD4^+^ T cells expressing CD69 in the spleen and cLN assessed by flow cytometry. (**F**) Representative gating pathway for CD8^+^Class I Tetramer^+^ in lungs and NALT. Frequency of S-specific tissue-resident memory (CD3^+^CD8^+^IV^-^CD44^+^Class I Tet^+^CD69^+^CD103^+^) CD8^+^ T cells in the (**G**) lungs and (**H**) NALT assessed by flow cytometry. (*n* = 5/immunized groups, *n* = 4/group PBS controls) Graphs representative of mean ± SEM with significance determined by one-way ANOVA with post-hoc Tukey’s multiple comparison test. *P < 0.05, **P < 0.01, ***P < 0.001, ****P < 0.0001 shown for select groups (full statistical analysis with exact *P* values provided in Dataset EV1). Source data are available online for this figure.

**Figure EV1:**
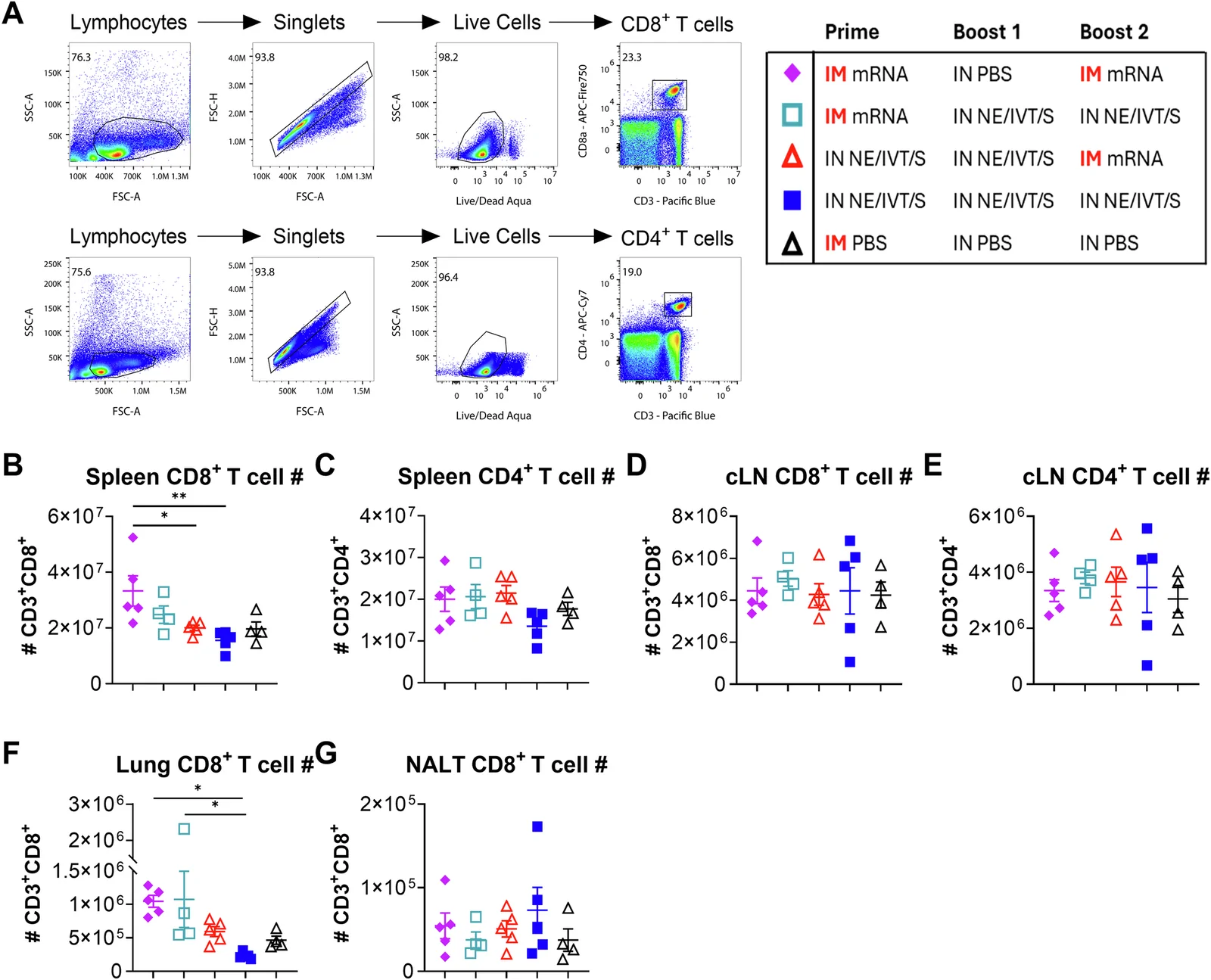
Homologous IM mRNA-LNP vaccination enhances the frequency of total CD8^+^ T cells within the circulation. Single cell suspensions from the spleens, cervical lymph nodes, lungs, and NALT of immunized C57BL/6 J mice were isolated 2 weeks post prime/boost/boost immunization. Cells were stained for viability, surface markers, and tetramer. (**A**) Parent gating for analysis. CD8^+^ T cell numbers within the (**B**) spleen, (**D**) cLN, (**F**) lungs, and (**G**) NALT. CD4^+^ T cell numbers within the (**C**) spleen and (**E**) cLN. Graphs representative of mean ± SEM with significance determined with one-way ANOVA with post-hoc Tukey’s multiple comparison test. (*n* = 4_5/immunized groups, *n* = 4/group PBS controls) **P < 0.01, ***P < 0.001, ****P < 0.0001 shown for select groups (full statistical analysis with exact *P* values provided in Dataset EV1).

Antigen-specific memory T cells of effector phenotype T_EM_s (CD44^+^ CD62L^-^) resident within the spleen and dLNs (gating Strategy in Fig. 2A and parent gating in Fig. EV1A) are specifically primed for immunosurveillance of the blood and tissues they drain (Beura et al, 2019; Shiow et al, 2006). Responses were therefore profiled from organs isolated 2 weeks post-final boost. Within the spleen, homologous IM mRNA immunization and heterologous immunization with IM mRNA and IN NE/IVT/S were effective in generating S-specific CD8^+^ T_EM_s (Fig. 2B) and CD4^+^ T_EM_s (Fig. 2C), which also expressed CD69, suggestive of residency; however, between heterologous regimens, parenteral priming with mucosal boost, as in IM mRNA→IN NE/IVT/S, generated higher frequencies of S-specific CD69^+^ CD4^+^ T_EM_s than priming mucosally, as with IN NE/IVT/S→IM mRNA. Homologous IM mRNA immunization increased the overall number of CD8^+^ T cells in the spleen compared to the other immunization regimens (Fig. EV1B), while both homologous IM mRNA and heterologous IM mRNA with IN NE/IVT/S regimens, regardless of sequence, had increased overall numbers of CD4^+^ T cells in the spleen (Fig. EV1C).

The cervical lymph nodes (cLNs) regulate upper respiratory tract immunity, making them an early site of viral detection and ideal for immunoprofiling the mucosal responses induced by IN immunization. Homologous IM mRNA and heterologous prime/boost/boost with IM mRNA and IN NE/IVT/S, regardless of sequence, induced S-specific CD69^+^ CD8^+^ T_EM_s within the cLNs, with slightly higher frequencies induced in the IM mRNA→IN NE/IVT/S group (Fig. 2D). We observed an even more pronounced benefit of heterologous IM→IN immunization on the CD4^+^ response. IM mRNA→IN NE/IVT/S immunization resulted in robust frequencies of S-specific CD69^+^ CD4^+^ T_EM_s within the cLNs compared to homologous IM mRNA, which induced minimal CD4^+^ T_EM_s. Notably, the reverse heterologous immunization sequence, IN NE/IVT/S→IM mRNA generated minimal or undetectable S-specific CD69^+^ CD4^+^ T_EM_s in the cLN (Fig. 2E). These results highlight that even though the mice received the same vaccines overall, the chronological sequence in which immune responses are primed systemically and then “pulled” to mucosal sites and boosted by IN immunization is most effective for inducing robust mucosal responses compared to systemically boosting mucosally primed immune responses. Accordingly, while significantly lower than the IM mRNA→IN NE/IVT/S group, the homologous IN NE/IVT/S regimen also resulted in induction of S-specific CD69^+^ CD4^+^ T_EM_’s in the cLN higher than both the IN NE/IVT/S→IM mRNA and homologous IM mRNA regimens. In contrast to what was observed in the spleen, no notable differences in the overall CD8^+^ or CD4^+^ T cell numbers in the cLN were observed between immunization groups (Fig. EV1D,E).

T_RM_s (CD44^+^CD69^+^CD103^+^IV^-^) within the lungs (gating strategy in Fig. 2F) play a major role in protection from respiratory viral infection by limiting viral replication before circulating T cells can be recruited (Pieren et al, 2023; Hassert and Harty, 2022; Szabo et al, 2021). The NALT located in the upper respiratory tract can also support antiviral T_RM_s, which limit viral dissemination to the lower respiratory tract (Pizzolla et al, 2017b, 2017a). Intriguingly, IM mRNA→IN NE/IVT/S generated the highest frequency of S-specific CD8^+^ T_RM_s (Fig. 2G) and had increased total CD8^+^ T cell numbers within the lungs (Fig. EV1F), whereas homologous IM mRNA and the reverse chronological sequence of IN NE/IVT/S→IM mRNA induced minimal S-specific CD8^+^ T_RM_s. Similarly, heterologous IM mRNA priming followed by IN NE/IVT/S boosting generated markedly higher S-specific CD8^+^ T cells within the NALT expressing tissue residency markers compared to all other immunization regimens (Fig. 2H), while all regimens had little effect on the overall total CD8^+^ T cell numbers (Fig. EV1G). These results further affirm that the sequence of heterologous immunization dramatically impacts the ability to foster CD8^+^ T_RM_s in the mucosa.

### Prime immunization route and immunogenicity imprint how subsequent booster immunizations shape T helper cell polarization

**Figure 3:**
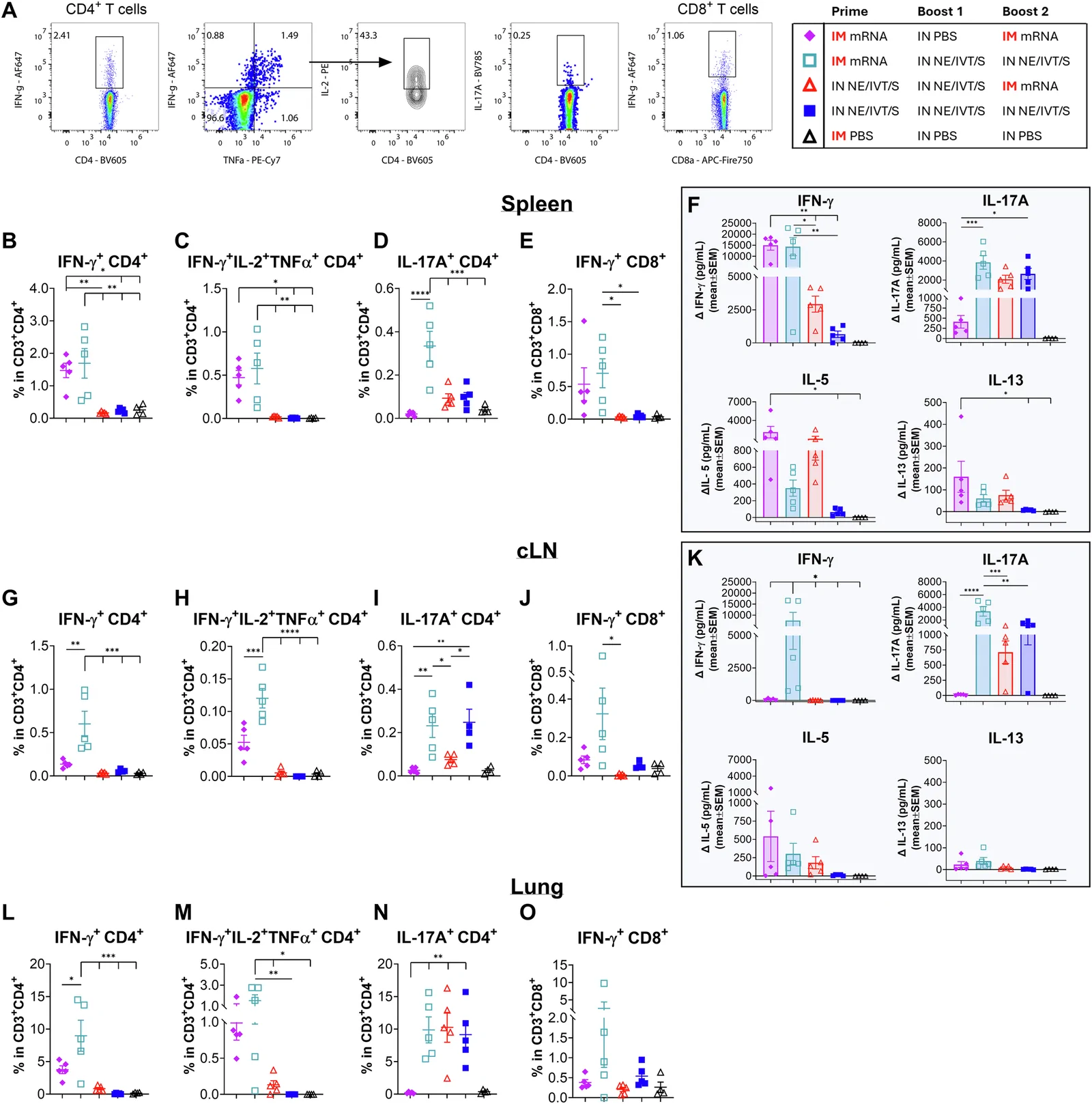
Heterologous IM mRNA prime; IN NE/IVT/S boost drives Th1/Th17 cellular immune responses at systemic and mucosal locales. Single cell suspensions from the spleens, cLNs, and lungs of immunized C57BL/6J mice were isolated 2 weeks post-prime/boost/boost and stimulated with 25 μg/mL WT S protein. (**A**) Representative gating of intracellular cytokine expression in CD4^+^ and CD8^+^ T cells. Cytokine expression in (**B**–**D**) CD4^+^ and (**E**) CD8^+^ T cells within the spleen 24 h after stimulation assessed by flow cytometry. (**F**) Cytokine production in supernatants from S-stimulated spleen cells after 3 d was measured by bead-based Luminex assay and normalized to media controls. Cytokine expression in (**G**–**I**) CD4^+^ and (**J**) CD8^+^ T cells within the cLN 24 h after stimulation assessed by flow cytometry. (**K**) Cytokine production in supernatants from S-stimulated cLN cells after 3 d was measured by bead-based Luminex assay and normalized to media controls. Cytokine expression in (**L**–**N**) CD4^+^ and (**O**) CD8^+^ T cells within the lungs 24 h after stimulation assessed by flow cytometry. (*n* = 4–5/immunized groups, *n* = 4/group PBS controls) Graphs representative of mean ± SEM with significance determined with one-way ANOVA with post-hoc Tukey’s multiple comparison test. *P < 0.05, **P < 0.01, ***P < 0.001, ****P < 0.0001 shown for select groups (full statistical analysis with exact *P* values provided in Dataset EV1). Source data are available online for this figure.

**Figure EV2:**
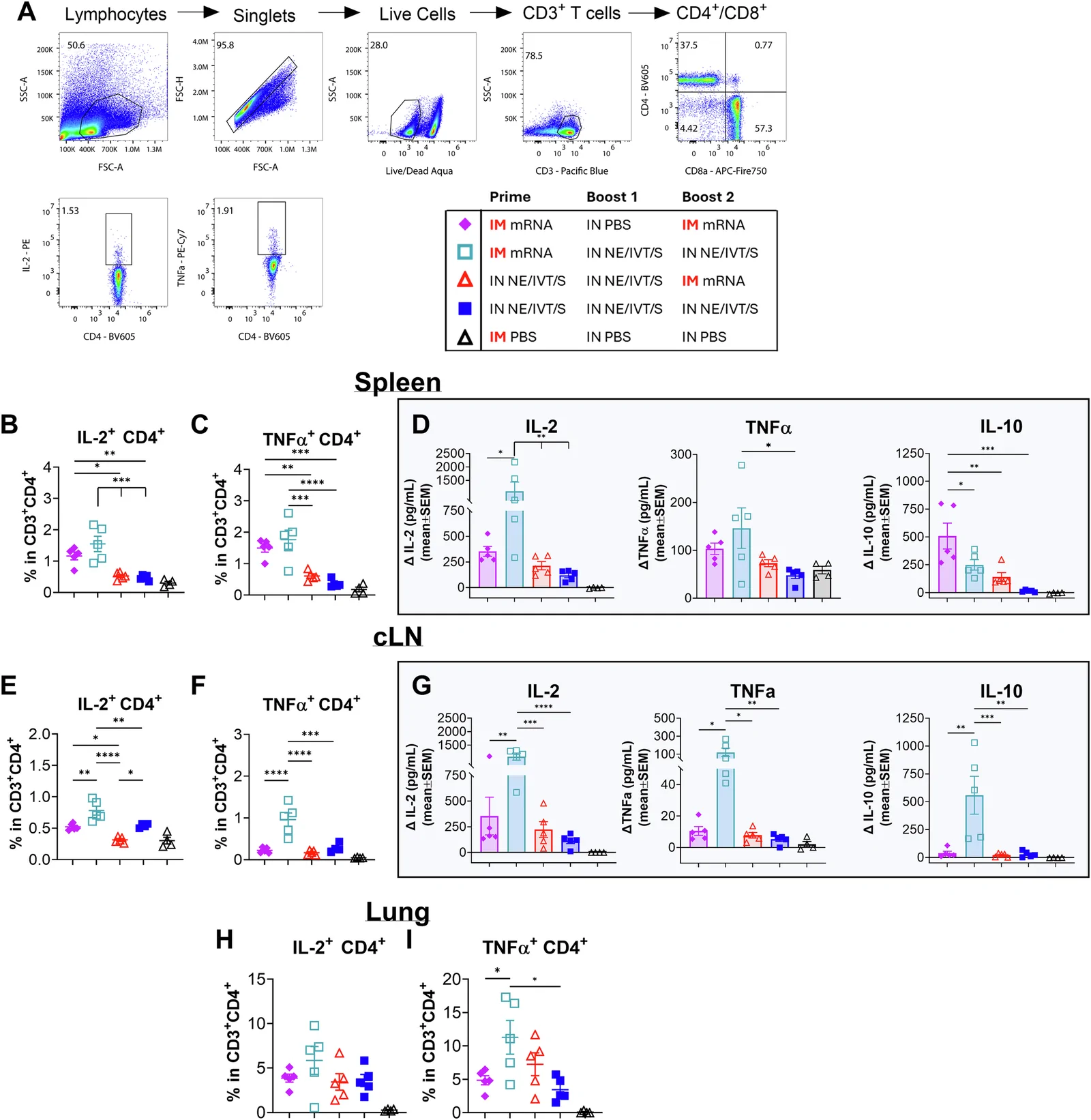
Heterologous immunization enhances Th1 response in spleen and cLN with high dependence on the sequence of immunization. Single cell suspensions from the spleens, cLN, and lungs of immunized C57BL/6J mice were isolated 2 weeks post prime/boost/boost immunization and stimulated with 25 μg/mL SARS-CoV2 WT S protein. (**A**) Cells were stained for viability, surface markers, and intracellular cytokines and gated accordingly. Frequency of IL-2 and TNFα expressing CD4^+^ T cells 24 h after stimulation in (**B**, **C**) spleen, (**E**, **F**) cLN, and (**H**, **I**) lung assessed by flow cytometry. Cytokine production in supernatants from S-stimulated (**D**) spleen and (**G**) cLN cells after 3 days was measured by bead-based Luminex assay and normalized to media controls. (*n* = 4–5/immunized groups, *n* = 4/PBS controls) Graphs representative of mean ± SEM with significance determined with one-way ANOVA with post-hoc Tukey’s multiple comparison test. *P* < 0.05, **P < 0.01, ***P < 0.001, ****P < 0.0001 shown for select groups (full statistical analysis with exact *P* values provided in Dataset EV1).

While it is clear S-specific T cells are optimally enhanced by the IM mRNA→IN NE/IVT/S heterologous vaccination regimen both systemically and in mucosal tissues, tetramer staining does not give a comprehensive picture of T-cell functionality. As IFN-γ^+^ and polyfunctional CD4^+^ and CD8^+^ T cells are strongly associated with viral control, cytokine recall responses were profiled in CD4^+^ and CD8^+^ T cells within these same tissues from vaccinated mice after the final boost immunization (Fig. 3A and parent gating in Fig. EV2A). Within the spleen, homologous IM mRNA and heterologous IM mRNA→IN NE/IVT/S generated similar frequencies of IFN-γ^+^-(Fig. 3B), IL-2^+^- (Fig. EV2B), TNFα^+^− (Fig. EV2C), and polyfunctional- (IFN-γ^+^IL-2^+^TNFα^+^) (Fig. 3C) CD4^+^ T cells, with the heterologous group inducing higher frequencies overall.

Consistent with our previous findings (Bielinska et al, 2014; Wong et al, 2021, 2015), all immunization regimens incorporating IN NE/IVT/S induced IL-17A^+^ CD4^+^ T cells within the spleen (Fig. 3D), further supporting that the Th17 response is uniquely associated with IN immunization with NE/IVT. Intriguingly, however, IM mRNA→IN NE/IVT/S was far superior to the reverse sequence and homologous IN NE/IVT/S at inducing IL-17A^+^CD4^+^ T cells during this short stimulation time period. Importantly, there was negligible production of IL-17A^+^ cells within the CD4^-^ population. High frequencies of IFN-γ^+^CD8^+^ T cells in the spleen were also induced in mice immunized with homologous IM mRNA or heterologous IM mRNA→IN NE/IVT/S, while levels were undetectable for the heterologous IN NE/IVT/S→IM mRNA group (Fig. 3E). We further profiled the Th polarization by examining cytokine secretion in the supernatants of S-stimulated splenocytes in comparison to unstimulated controls. Unstimulated controls showed minimal cytokine secretion. Significant S-specific Th1 responses were generated in the heterologous IM mRNA→IN NE/IVT/S and the homologous IM mRNA groups, as evidenced by markedly increased IFN-γ production (Fig. 3F, top). However, heterologous immunization showed greater enhancement in IL-2 and TNFα compared to the homologous IM mRNA group (Fig. EV2D). In contrast, IN NE/IVT/S→IM mRNA showed dramatically reduced IFN-γ production in the spleen compared to these groups. Splenocytes from the homologous IM mRNA group also showed marked Th2 polarized responses as evidenced by increased IL-5 (Fig. 3F, bottom) and IL-13 (Fig. 3F, bottom). In contrast, the homologous IN NE/IVT/S group showed minimal induction of these cytokines. Interestingly, while IL-5 in the spleen was substantially reduced in the heterologous IM mRNA→IN NE/IVT/S group compared to the homologous IM mRNA group, the reverse heterologous IN NE/IVT/S→IM mRNA sequence showed high levels of IL-5. These findings suggest that IM mRNA boosting of the mucosally NE/IVT/S primed responses results in a more Th2-biased response compared to NE/IVT/S mucosal boosting of the systemically IM mRNA primed responses, even though these mice received the same overall vaccinations. Consistent with our flow cytometry results, all groups receiving IN immunization with NE/IVT/S showed significant induction of S-specific IL-17A within the spleen (Fig. 3F, top). IL-10 was also induced by these heterologous groups; however, the highest IL-10 was induced by the homologous IM mRNA regimen (Fig. EV2D). Overall, these results suggest that the sequence of heterologous immunization affects the polarization of the cytokine response systemically. While homologous IM mRNA generates a systemic antigen-specific Th1/Th2 profile and homologous IN NE/IVT/S generates a systemic Th1/Th17 profile, heterologous IM mRNA→IN NE/IVT/S shifts the profile heavily towards magnified Th1/Th17 responses, and heterologous IN NE/IVT/S→IM mRNA shifts the profile to a more heavily Th2/Th17 skewed profile.

Within the cLNs, mucosal boosting was critical to driving CD4^+^ T cell cytokine responses in heterologous immunization. Heterologous IM mRNA→IN NE/IVT/S immunization markedly enhanced IFN-γ^+^- (Fig. 3G), IL-2^+^- (Fig. EV2E), TNFα^+^- (Fig. EV2F), and polyfunctional IFN-γ^+^IL-2^+^TNFα^+^- (Fig. 3H) CD4^+^ T cells. Intriguingly, only immunization regimens with IN NE/IVT/S as a boost (heterologous IM mRNA→IN NE/IVT/S and homologous IN NE/IVT/S) induced robust IL-17A^+^ CD4^+^ T cells within the cLN (Fig. 3I). We also noted distinctly enhanced frequencies of IFN-γ^+^− (Fig. 3J) CD8^+^ T cells in the cLNs from IM mRNA→IN NE/IVT/S mice compared to all other immunization regimens. Analogous to our flow cytometry data, heterologous IM mRNA→IN NE/IVT/S immunization generated a significant IFN-γ (Fig. 3K, top), IL-2, and TNFα (Fig. EV2G) response to S-protein within the cLNs, which was not observed in heterologous IN NE/IVT/S→IM mRNA mice. We noted overall minimal generation of the Th2 cytokines, IL-5 and IL-13 (Fig. 3K, bottom) within the cLN in response to antigen stimulation, with minor differences between immunization regimens. As with spleen, an S-specific IL-17A response (Fig. 3K, top) was induced in the cLNs of all mice given an IN NE/IVT/S immunization. Heterologous IM mRNA→IN NE/IVT/S prime-pull immunization gave enhanced IL-17A compared to homologous IN NE/IVT/S immunization and heterologous immunization with mucosal priming (IN NE/IVT/S→IM mRNA). Furthermore, IL-10 (Fig. EV2G) was also significantly produced in the cLNs of mice given heterologous IM mRNA→IN NE/IVT/S compared to all other regimens. These results further confirm that the chronological sequence of adjuvanted mucosal boosting of systemically primed immune responses optimally drives localization of antigen-specific responses to the respiratory draining lymph nodes and promotes the most effective Th1/Th17 polarized cytokine profile. In contrast, systemic boosting of mucosally primed immune responses not only less effectively promotes T cell responses in the mucosa but also significantly shifts the polarization of these responses towards a less ideal Th2/Th17 profile. Taken together, these results demonstrate how simply altering the sequence of immunization with the same overall vaccination regimen has drastic effects on both immune response polarization and localization.

Similar to cLN, mucosal boosting and the presence of the IN NE/IVT adjuvant drove T cell cytokine responses within the lungs. IM mRNA→IN NE/IVT/S induced remarkably high frequencies of IFN-γ^+^− (Fig. 3L), IL-2^+^- (Fig. EV2H), TNFα^+^- (Fig. EV2I), and polyfunctional IFN-γ^+^IL-2^+^TNFα^+^-(Fig. 3M) CD4^+^ T cells than other immunization regimens. While IL-2^+^-, TNFα^+^, and IL-17A^+^- expressing CD4^+^ T cells were generated in heterologous (IM mRNA→IN NE/IVT/S and IN NE/IVT/S→IM mRNA) and homologous IN NE/IVT/S immunizations (Fig. 3N), priming with IM mRNA was necessary for generating a significant IFN-γ response within the lungs (Fig. 3L). Interestingly, while IM mRNA→IN NE/IVT/S clearly induced significantly higher IL-17A^+^ CD4^+^ T cells in the mucosal dLNs than the reverse sequence, levels were equivalent between these groups within the lungs (Fig. 3I,N). This may reflect the strong Th17-inducing properties of the NE/IVT adjuvant, as observed with the homologous IN NE/IVT/S regimen in the lungs, which may establish resident Th17 cells that can be boosted through systemic mediators induced by IM mRNA vaccination that can reach the lungs. In addition, IM mRNA→IN NE/IVT/S also generated the highest frequencies of IFN-γ^+^ (Fig. 3O) CD8^+^ T cells in the lungs compared to other immunization regimens. T cell cytokine profiling within these immune compartments strongly demonstrates the effectiveness of the IN NE/IVT adjuvant in both redirecting systemically primed immune responses to the respiratory mucosa and optimally enhancing these responses. These results firmly underscore the importance of chronological sequence used in heterologous prime/boost immunization regimens in shaping the induced T-cell responses and dictating their localization.

### Heterologous immunization regimens with IN NE/IVT/S afford sterilizing immunity to lethal challenge, with major distinctions in post-challenge cytokine profiles based on chronological order of vaccination

**Figure 4:**
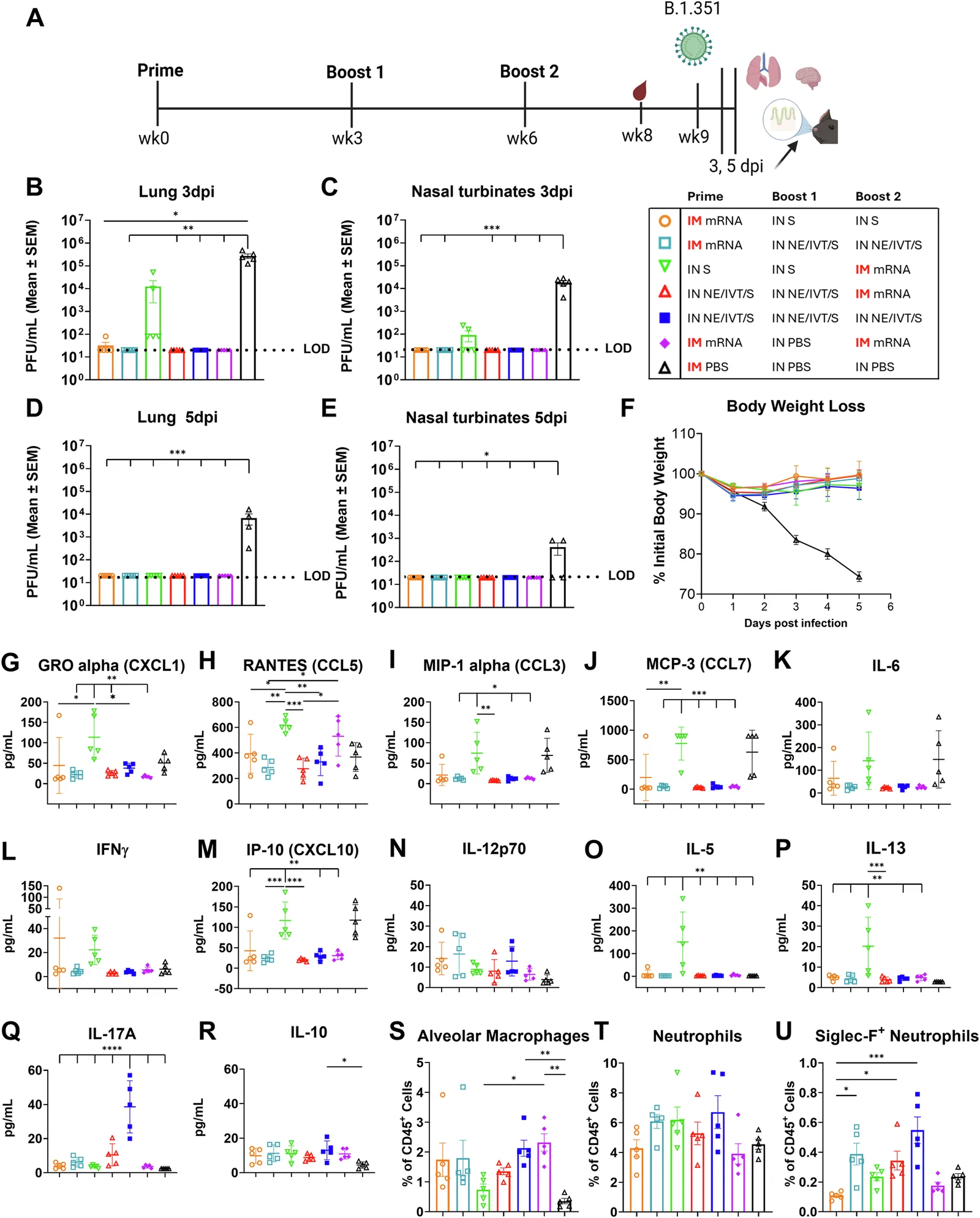
Heterologous immunization with NE/IVT adjuvanted spike is protective in a lethal infection model of SARS-CoV-2. (**A**) K18-hACE2 mice were immunized according to a prime/boost/boost schedule and then challenged IN with 10^4^ PFU of B.1351 SARS-CoV-2, 3 weeks post-final boost. Viral titers were quantified in homogenates from the (**B**, **D**) lungs and (**C**, **E**) nasal turbinates by plaque assay at the indicated time points post-challenge. (**F**) Weight loss in mice was monitored daily post-infection. (**G**–**R**) Chemokine and cytokine levels within the lungs of mice at 3 dpi were detected by bead-based Luminex. (**S**) Alveolar macrophages, (**T**) total neutrophils, and (**U**) Siglec-F^+^ neutrophils as a percentage of CD45^+^ cells in dissociated lungs of infected mice collected at 3 dpi, assessed by flow cytometry. Graphs representative of mean ± SEM with significance determined with Kruskal–Wallis test (viral titers, flow cytometry) with post-hoc Dunn’s multiple comparison test or one-way ANOVA (Luminex) or two-way ANOVA (Weight Loss) with post-hoc Tukey’s multiple comparison test. (*n* = 5/immunized groups, *n* = 4–5/group PBS controls) *P < 0.05, **P < 0.01, ***P < 0.001, ****P < 0.0001 for select groups (full statistical analysis with exact *P* values provided in Dataset EV1). Source data are available online for this figure.

As the immunization regimens shaped vastly differing humoral and cellular profiles at mucosal and systemic sites, we assessed how this impacted responses to infection. To this end, we utilized a lethal challenge model with heterologous B.1.351 SARS-CoV-2 in K18-hACE2 mice (Fig. 4A) (Radvak et al, 2021). This model has the potential to induce lung and brain pathology along with chemokine/cytokine storm (Oladunni et al, 2020). Quantification of serum WT S-specific IgG (Appendix Fig. S2A) and nAbs to WT (Appendix Fig. S2B) and B.1.351 (Appendix Fig. S2C) PSVs reflected the results obtained in WT C57Bl/6 mice, with lower titers induced by heterologous regimens with IM mRNA and unadjuvanted IN S. Three weeks post-final boost, mice were challenged IN with 1 × 10^4^ pfu B.1.351 SARS-CoV-2. In control PBS immunized mice, high viral titers were present in the lungs (Fig. 4B) and nasal turbinates (NTs) (Fig. 4C) 3 days post infection (dpi), which persisted to 5 dpi (Fig. 4D,E), with significant morbidity reflected by weight loss (Fig. 4F). Virus was also detected in the brain at 5 dpi, indicating viral dissemination (Appendix Fig. S2D,E). Additionally, viral loads were detected at 3 dpi in the lungs and NTs of select mice in groups given heterologous IM mRNA and unadjuvanted IN S, with IM mRNA→IN S providing better protection than IN S→IM mRNA. However, by 5 dpi, no virus was detected in the lungs or NTs, reflecting faster viral clearance than unimmunized controls. Notably, both IM mRNA→IN S and IN S→IM mRNA groups showed no weight loss, pointing to a reduction in morbidity even in the presence of viral infection. In contrast, homologous IM mRNA or IN NE/IVT/S, as well as heterologous regimens with IM mRNA and IN NE/IVT/S (regardless of sequence), imparted sterilizing immunity in both the upper and lower respiratory tracts, demonstrating more complete protection with these vaccination regimens.

**Figure EV3:**
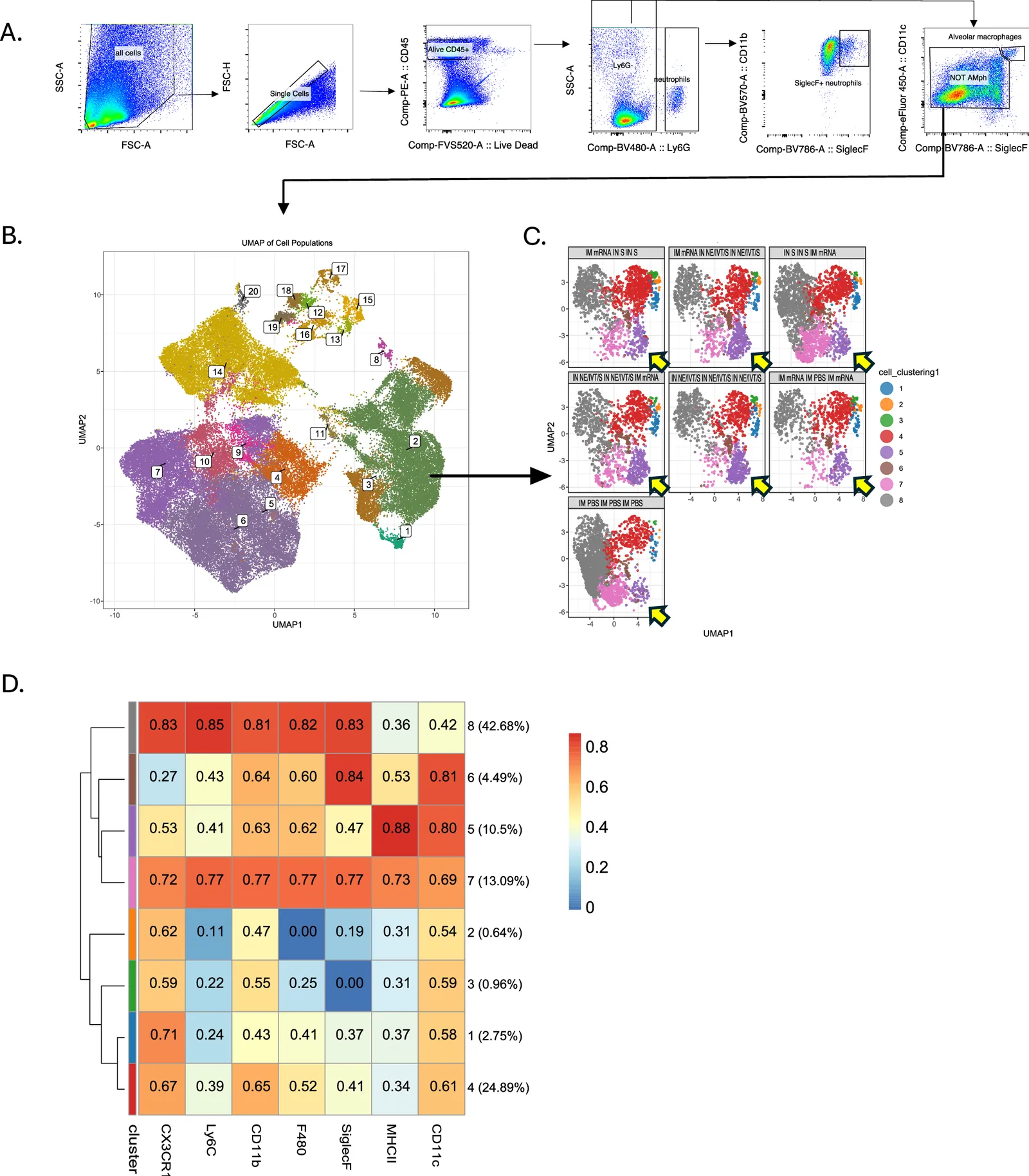
Vaccination regimen shapes the myeloid cell infiltration in the lungs of infected mice. (**A**) Gating strategy to identify total neutrophils (Ly6G^+^), activated neutrophils (SiglecF^+^), and alveolar macrophages (CD11c^+^SiglecF^+^). (**B**) Lung immune cells (CD45^+^) that were not alveolar macrophages or neutrophils were further subdivided into 20 clusters by unsupervised analysis using the following markers: CD11b, CD11c, SiglecF, MHCII, F4/80, Ly6c, Arginase, iNOS SIRPa, and CX3CR1 to investigate myeloid cell populations in lungs of infected K18-hACE2 mice. (**C**) Cluster 2 was further clustered into eight subclusters using the following markers: CD11b, CD11c, SiglecF, MHCII, F4/80, Ly6c, and CX3CR1, and revealed cell populations that were enriched depending on the vaccination regimen. Subcluster 5 is enriched for the vaccination groups that received NE/IVT/S (yellow arrows). (**D**) Heatmap representing expression levels for markers used for subclustering cluster 2. Cluster numbers are given on the right, followed by the percentage of cells represented by the specific cluster between parentheses (*n* = 5/group).

Chemokine and cytokine responses were profiled in clarified lung homogenates from vaccinated mice at 3 dpi to determine how the immunization regimens shaped early responses to infection. It is important to note that the cytokine response post-challenge is influenced both by the vaccination regimen and by the extent of productive viral infection. While both the heterologous IM mRNA→IN NE/IVT/S and IN NE/IVT/S→IM mRNA groups showed minimal cytokine production in the lung homogenate (with no inflammatory markers) due to robust blockage of viral infection, clear differences in cytokine profiles that were dependent upon chronological sequence of immunization could be observed in groups given the suboptimal heterologous regimens of IM mRNA and unadjuvanted IN S. For the IN S→IM mRNA group, increased inflammatory markers such as GROα (CXCL1) (Fig. 4G) and RANTES (CCL5) (Fig. 4H) were uniquely enhanced post-challenge beyond levels induced in unvaccinated-challenged mice. Other inflammatory markers, MIP-1α (CCL3) (Fig. 4I), MCP-3 (CCL7) (Fig. 4J), IL-6 (Fig. 4K), and IP-10 (Fig. 4M) were also elevated in this group to levels comparable to unvaccinated-challenged mice. Moreover, the Th2 polarized responses observed in in vitro T cell antigen recall profiling post-vaccination in this group were similarly reflected by high levels of IL-5 (Fig. 4O) and IL-13 (Fig. 4P) in the lungs post-challenge, with low levels of IFNγ also induced (Fig. 4L). Notably, these cytokines were not detected in unvaccinated-challenged mice, demonstrating the impact of vaccination on shaping the host response to infection. In contrast, the reverse IM mRNA→IN S group did not show enhanced Th2 cytokines nor enhancement in inflammatory markers, reflecting both more complete protection as well as the different T cell profile induced by this sequence. RANTES (CCL5) was also high in the lungs of challenged mice that received homologous IM mRNA (but none of the inflammatory markers). Finally, while no IL-17A was induced in unvaccinated-challenged mice, significant but low levels of IL-17A could be detected in the lungs of homologous IN NE/IVT/S vaccinated mice post-challenge as well as in the heterologous groups given IM mRNA and IN NE/IVT/S, albeit at even lower levels, reflecting the Th17 skewing induced by the IN NE/IVT adjuvant (Fig. 4Q). Low levels of IL-12p70 and IL-10 were also induced with all immunization regimens (Fig. 4N,R). Interestingly, even though significant levels of inflammatory markers and Th2 cytokines were observed in heterologous IN S→IM mRNA-immunized mice, no weight loss was observed, suggesting potentially significant differences in the cellular mediators contributing to protection between these immunization groups. Alveolar macrophages form the first line of innate immune cell defense in the lung during respiratory infection. Analysis of innate immune cell populations in the lungs revealed that alveolar macrophages were absent in PBS control mice (Fig. 4S and gating scheme in Fig.  EV3A), suggesting that vaccination was needed to preserve them by 3 dpi. After infection, all animals had similar levels of infiltrating neutrophils (Fig. 4T). However, mice that received IN NE/IVT/S vaccination as part of any vaccination regimen all showed higher levels of activated SiglecF^+^ neutrophils (Fig. 4U), which have been described as a protective effect of vaccination and damage repair associated with Th17 responses and the induction of CXCL1 (Borkner et al, 2021; Hall et al, 2024; Tavares et al, 2025). We further observed vaccine regimen-specific changes in the myeloid cell composition of the lungs of infected mice (Fig. EV3). Respiratory virus infection induces rapid remodeling of the pulmonary myeloid landscape, generating phenotypically and functionally heterogeneous populations of resident and recruited cells exhibiting intermediate maturation and activation states. Therefore, we took an unsupervised approach to address the complexity of the pulmonary myeloid compartment after exclusion of neutrophils (Ly6G^+^) and alveolar macrophages (CD11c^+^SiglecF^+^) (Gating scheme provided in Fig. EV3A). Unsupervised clustering identified 20 distinct cell cluster sets. We found that cluster 2 comprised the largest proportion of cells and was predominantly enriched in unvaccinated animals but also had a subcluster that was more enriched in IN NE/IVT/S homologous vaccinated animals, or IM mRNA→IN NE/IVT/S animals (Fig. EV3B). Further subclustering of cluster 2 revealed subcluster 5 (yellow arrows in Fig. EV3C) that represents dendritic cells (MHCII^+^CD11c^+^), suggesting early immune coordination between innate and adaptive immunity. Further future in-depth characterization will allow for improved understanding of these myeloid clusters observed in the lungs of infected mice. However, it is clearly evident by this cytokine and immune cell profiling of the lungs that although protection can be achieved with these different vaccination regimens, each differentially shapes the subsequent immune response to infection.

### Heterologous prime-pull immunization induces a more durable cellular response than homologous immunization regimens

**Figure 5:**
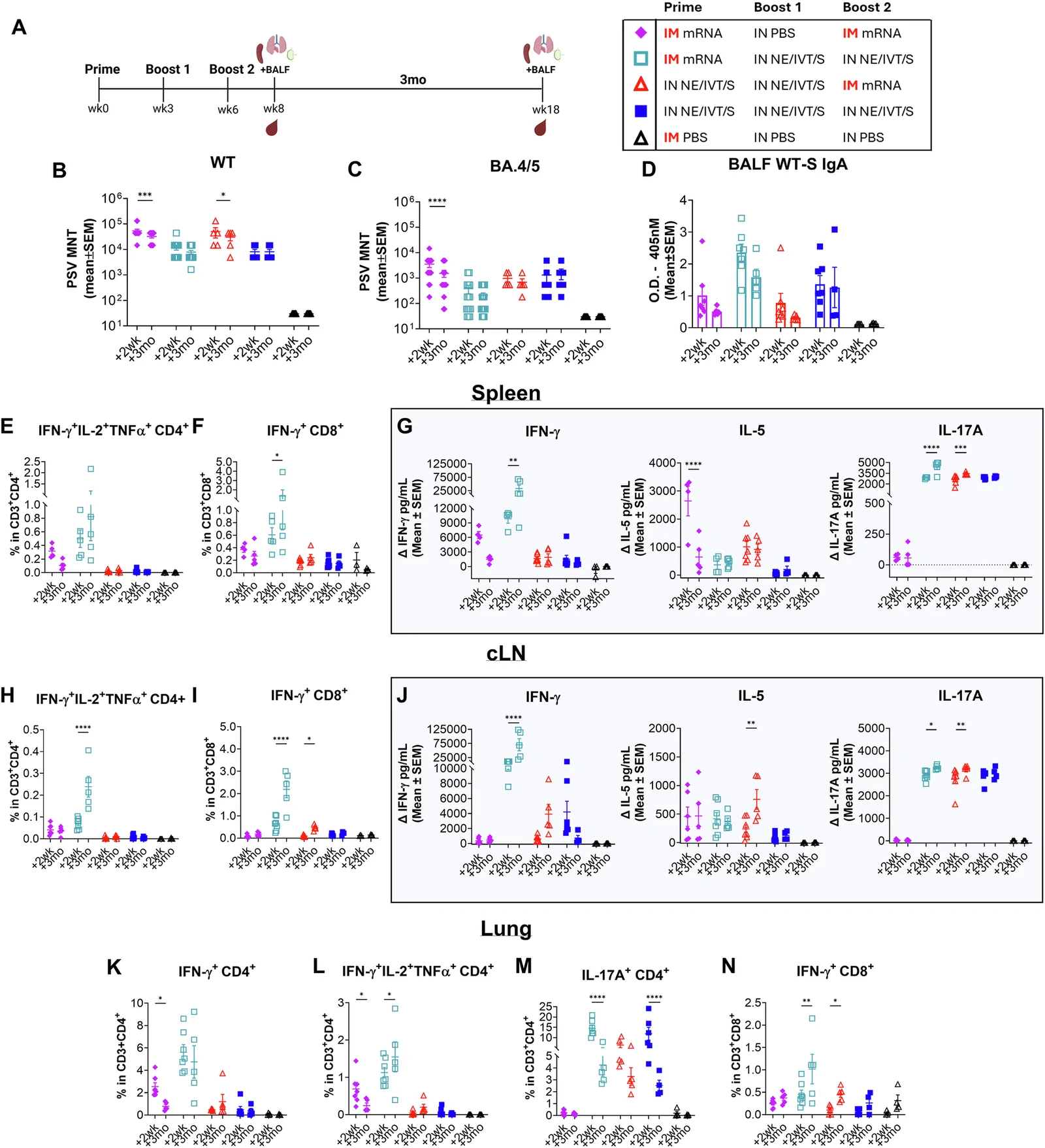
Heterologous immunization induces more durable immune responses than homologous immunization, with cellular localization and Th bias shaped by the sequence in the route of immunization. (**A**) C57BL/6J mice were immunized according to a prime/boost/boost schedule at 3-week intervals, and immune response profiles were compared between week 8 (+2 week post-final boost) and week 18 ( + 3 m post-final boost). Serum nAb titers to (**B**) WT and (**C**) BA.4/5 variants as measured by PSV microneutralization assay for mice given homologous IM mRNA, IM mRNA→IN NE/IVT/S (*n* = 15/grp), IN NE/IVT/S→IM mRNA (*n* = 5), or homologous IN NE/IVT/S (*n* = 6) compared to PBS controls (*n* = 12). Group sizes were selected as a proportion of mice that were given an additional booster in Fig. 7. (**D**) BALF IgA specific to WT-S assessed by ELISA (*n* = 4–7/immunized groups, *n* = 4/group PBS controls). Single cell suspensions of spleens, cLNs, and lungs harvested from vaccinated mice at each time point were stimulated with 25 μg/mL WT S protein, and cytokine expression was profiled. Frequencies of cytokine-expressing (**E**, **F**) CD4^+^ and CD8^+^ T cells in the spleen 24 h post stimulation assessed by flow cytometry. (**G**) Cytokine production in supernatants from S-stimulated splenocytes after 3 days was measured by bead-based Luminex assay and normalized to media controls. Frequencies of cytokine-expressing (**H**, **I**) CD4^+^ and CD8^+^ T cells in the cLN 24 h post-stimulation assessed by flow cytometry. (**J**) Cytokine production in supernatants from S-stimulated cLN cells after 3 days was measured by bead-based Luminex assay and normalized to media controls. Frequencies of cytokine-expressing (**K**–**N**) CD4^+^ and CD8^+^ T cells in the lungs 24 h post-stimulation assessed by flow cytometry. (*n* = 4–7/immunized groups, *n* = 3–4/PBS controls) Graphs representative of mean ± SEM with significance of durability effects within each treatment type determined by two-way ANOVA (MNT and Flow cytometry) or mixed-effects model (Serology and Luminex) with post-hoc Tukey’s multiple comparison test. *P < 0.05, **P < 0.01, ***P < 0.001, ****P < 0.0001 shown for select groups (full statistical analysis with exact *P* values provided in Dataset EV1). Source data are available online for this figure.

As durability is a major limitation facing IM mRNA vaccines, we next sought to evaluate whether heterologous vaccination regimens with IN NE/IVT/S could improve the durability of induced immune responses. We thus compared humoral, mucosal and cellular immunity 2 weeks or 3 months post-final boost immunization (week 8 or 18, respectively) (Fig. 5A). Overall, all regimens evaluated for longevity (homologous IM mRNA, homologous IN NE/IVT/S, and heterologous IM mRNA→IN NE/IVT/S or IN NE/IVT/S→IM mRNA) showed largely maintained serum nAbs against both WT (Fig. 5B) and BA.4/5 (Fig. 5C) variants over the 3 month period from the final immunization, with only the homologous IM mRNA group showing a decrease in titers against BA.4/5. In BALF, WT S-IgA responses, which were strongly induced by heterologous IM mRNA→IN NE/IVT/S immunization, were highly durable at 3 months post-final boost. IgA in the IM mRNA→IN NE/IVT/S group at this late time point remained higher, in fact, than levels induced by the homologous IM mRNA and homologous IN NE/IVT/S groups proximal to the final boost immunization (week 8) (Fig. 5D). Homologous IN NE/IVT/S also imparted relatively durable IgA responses, albeit at lower levels than heterologous IM mRNA→IN NE/IVT/S, while both homologous IM mRNA and heterologous IN NE/IVT/S→IM mRNA induced low WT S-IgA, which diminished to baseline levels after 3 months.

Durability of the cellular response was then assessed in isolates from the spleens, cLNs, and lungs. Intracellular cytokine profiling of the spleen in homologous IM mRNA mice revealed a pronounced drop over the 3-month interval in frequencies of IFN-γ^+^- (Appendix Fig. S3A) and IFN-γ^+^IL-2^+^TNFα^+^ polyfunctional CD4^+^ T cells (Fig. 5E) in response to WT S stimulation. In contrast, the frequencies of these cytokine-expressing CD4^+^ T cells within the spleen for the heterologous IM mRNA→IN NE/IVT/S group showed remarkable durability, with similar, or in fact higher frequencies at 3 months post- compared to 2 weeks post-final boost. Frequencies of IL-17A^+^ CD4^+^ T cells were also maintained over this period for all groups given a mucosal immunization of IN NE/IVT/S regardless of regimen (Appendix Fig. S3B). Similarly, frequencies of IFN-γ^+^ CD8^+^ T cells slightly decreased within the spleen of homologous IM mRNA mice over the 3-month interval, whereas those for the heterologous IM mRNA→IN NE/IVT/S group were elevated and highly durable (Fig. 5F). Profiling of cytokine production during the antigen-recall response via multiplex immunoassay further confirmed that the Th1 cellular responses in the heterologous IM mRNA→IN NE/IVT/S group was maintained, and in fact, elevated over this time period, with increased IFN-γ (Fig. 5G), IL-2 and TNFα (Appendix Fig. S3C) between week 8 and week 18. In contrast, the Th1 and Th2 splenic cellular responses induced in homologous IM mRNA-immunized mice were more transient, with significant decreases in IFN-γ, IL-5 (Fig. 5G), IL-2, and IL-10 (Appendix Fig. S3C) observed after 3 months. Notably, IL-17A (Fig. 5G) in the spleen was maintained or increased over this interval in all regimens which included a mucosal immunization with NE/IVT/S. Interestingly, IL-5 production in the spleen for heterologous IN NE/IVT/S→IM mRNA mice was maintained over 3 months (Fig. 5G), further confirming prolonged cellular responses induced by heterologous immunization and the long-term maintenance of the distinct T cell polarization biases primed by the same vaccine regimens simply given in reverse chronological sequence.

Within cLNs, heterologous IM mRNA→IN NE/IVT/S immunization generated high frequencies of IFN-γ^+^- (Appendix Fig. S3D), IFN-γ^+^IL-2^+^TNFα^+^- (Fig. 5H), and IL-17A^+^- (Appendix Fig. S3E) CD4^+^ T cells that further increased over 3 months, suggesting a unique cellular programming for long-term maintenance of these populations within the mucosal dLNs. Given the low initial frequencies of these cytokine-expressing cells induced by the other immunization regimens, no significant changes were observed over this duration. Furthermore, heterologous IM mRNA→IN NE/IVT/S immunization fostered remarkably durable IFN-γ^+^ CD8^+^ T cells in the cLN (Fig. 5I), which increased over 3 mo. Profiling secreted cytokines after antigen stimulation confirmed heterologous IM mRNA→IN NE/IVT/S immunization programs a significantly more durable Th1 response in the cLN, revealing profound increases in IFN-γ (Fig. 5J) and IL-2, with maintained TNFα (Appendix Fig. S3F) production. The Th2 bias imparted by heterologous IN NE/IVT/S→IM mRNA was also durable, displaying a modest increase in IL-5 (Fig. 5J) production in the cLN over the 3 months. IL-10 (Appendix Fig. S3F) production within the cLN was also better maintained with IM mRNA→IN NE/IVT/S immunization compared to homologous IN NE/IVT/S. Despite these unique differences, IL-17A responses in the cLN (Fig. 5J), were equally resilient in mice given IN NE/IVT/S in either heterologous or homologous regimens.

Similar to the spleen, IFN-γ^+^- (Fig. 5K) and IFN-γ^+^IL-2^+^TNFα^+^- (Fig. 5L) CD4^+^ T cells significantly decreased in the lungs of homologous IM mRNA-immunized mice after 3 mo. In contrast, IFN-γ^+^- and polyfunctional IFN-γ^+^IL-2^+^TNFα^+^- CD4^+^ T cells were maintained with heterologous IM mRNA→IN NE/IVT/S immunization, suggesting induction of durable Th1 immune responses within the lungs. Despite maintained Th17 responses in the cLN and spleen with mucosal NE/IVT/S immunization, significant reductions in the frequencies of IL-17A^+^- (Fig. 5M) CD4^+^ T cells were observed in the lungs over the 3-month period in mice receiving IN NE/IVT/S immunization in either heterologous or homologous regimens. This suggests that the vaccine-induced long-term antigen-specific Th17 responses are maintained in the lymphoid organs rather than within the mucosal tissue. Similar to spleen and cLN, heterologous IM mRNA→IN NE/IVT/S induced a durable and enhanced frequency of IFN-γ^+^- (Fig. 5N) CD8^+^ T cells in the lungs over time.

These results clearly demonstrate that heterologous immunization induces a more durable humoral and cellular immune response than homologous immunization regimens. However, systemic priming followed by adjuvanted mucosal boosting establishes significantly more robust mucosal IgA responses and durable cellular immunity within the local mucosal lymphoid tissue. Induction of these local, durable, and optimally polarized Th1/17 responses poised within the mucosal draining lymph nodes is a major advantage in facilitating rapid viral clearance in the respiratory tract. Moreover, these durability studies demonstrate the long-lasting impact of the initial vaccination sequence in shaping the ultimate overall cellular response profiles.

### Intramuscular mRNA immunization induces local and distal IFN-I expression, while intranasal NE/IVT/S induces robust localized IFN-I expression

**Figure 6:**
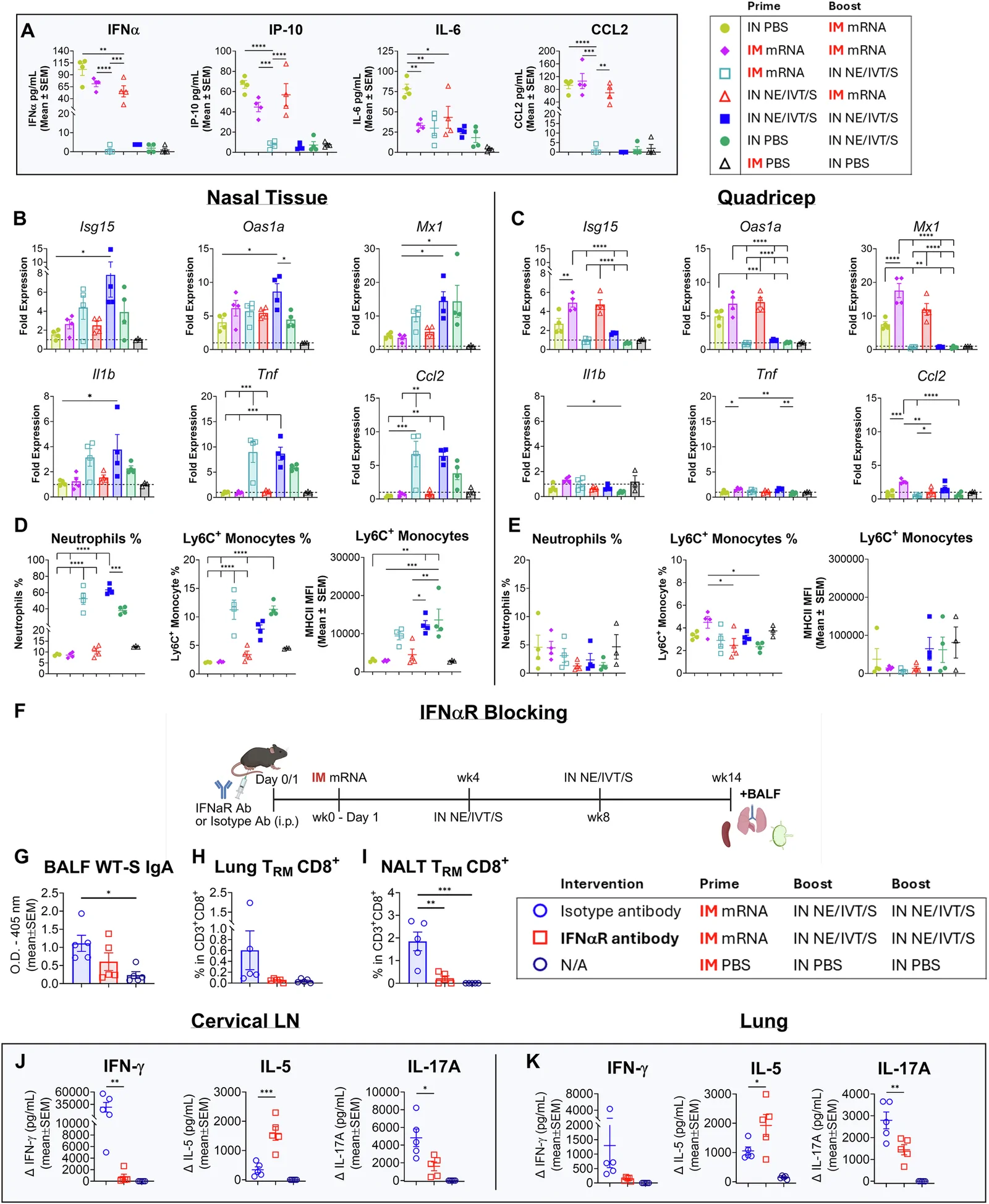
IM mRNA immunization induces a systemic IFN-I response priming subsequent heterologous route immunization efforts. C57BL/6 J mice were immunized in a prime/boost schedule at a 4-week interval. (**A**) Serum levels of IFNα, IP-10, IL-6, and CCL2 were assessed 8 h post-immunization at the booster timepoint by bead-based Luminex assay. (*n* = 4/group) Expression of genes of interest within the (**B**) nasal tissue and (**C**) quadriceps 24 h post-immunization at the booster timepoint assessed by RT-qPCR. Frequency of neutrophils and Ly6C^+^ monocytes in total live singlet cells and MHCII median fluorescent expression in Ly6C^+^ monocytes in (**D**) nasal tissue and (**E**) quadriceps 24 h post-immunization assessed by flow cytometry. (*n* = 3–4/group) (**F**) IFNαR surface cellular expression was transiently abrogated with IFNαR blocking antibody before the prime immunization. C57BL/6J mice were subsequently immunized in a prime/boost/boost schedule at 4-week intervals. (**G**) WT S-specific IgA in BALF after prime/boost/boost immunization assessed by ELISA. Frequencies of S-specific tissue-resident memory (CD3^+^CD8^+^IV^-^CD44^+^Class I Tet^+^CD69^+^CD103^+^) CD8^+^ T cells in (**H**) lung and (**I**) NALT assessed by flow cytometry. Cytokine production in supernatants from S-stimulated (**J**) cLN and (**K**) lung cells after 3 d was measured by bead-based Luminex assay and normalized to media controls (*n* = 5/group). Graphs representative of mean ± SEM with significance determined by one-way ANOVA with post-hoc Tukey’s multiple comparison test. *P < 0.05, **P < 0.01, ***P < 0.001, ****P < 0.0001 shown for select groups (full statistical analysis with exact *P* values provided in Dataset EV1). Source data are available online for this figure.

**Figure EV4:**
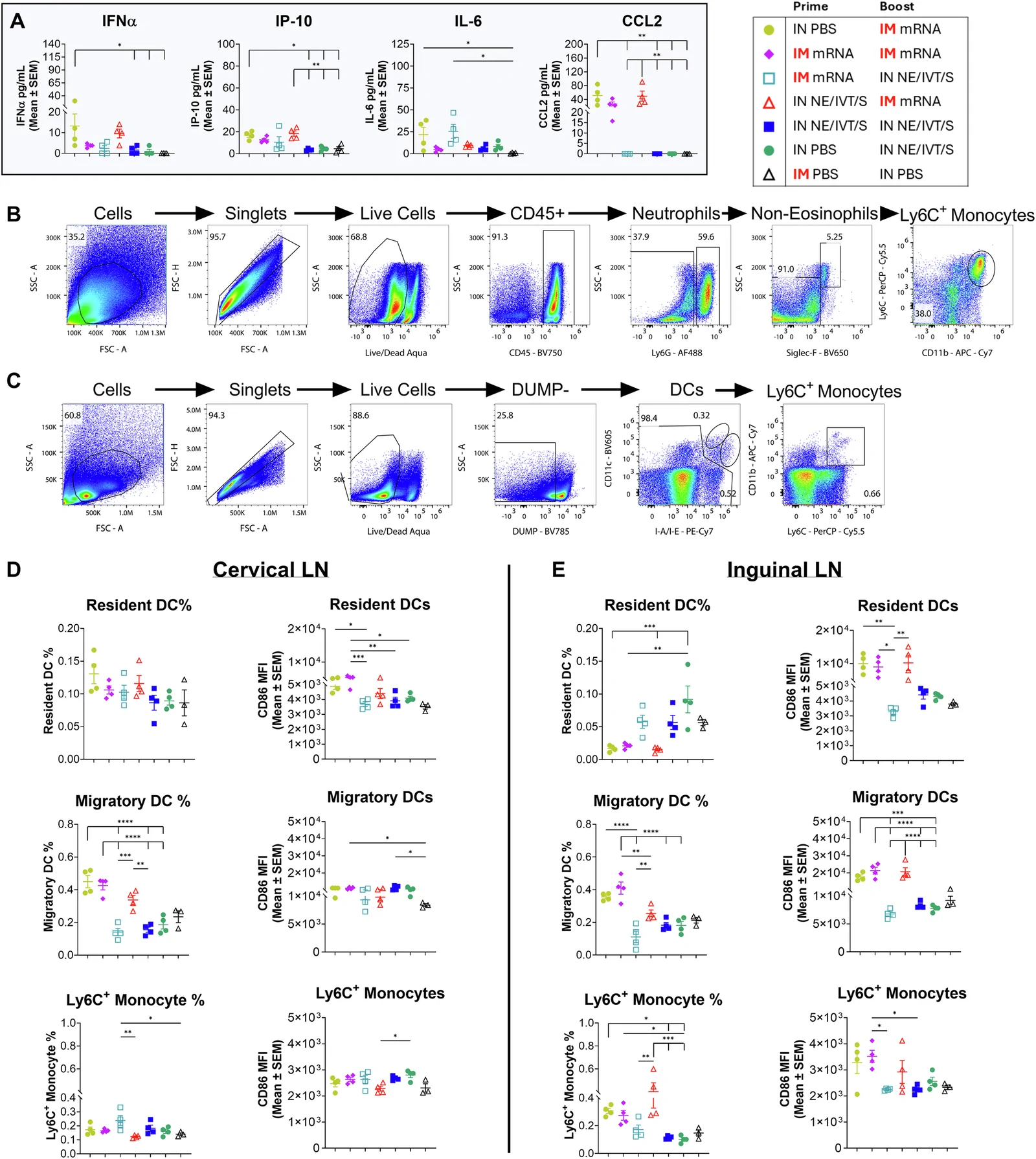
IM mRNA immunization induces enhanced inflammatory responses both locally and distally, while IN NE/IVT/S immunization induces only local inflammatory effects. C57BL/6J mice were immunized in a prime/boost schedule at 4-week intervals. (**A**) Serum levels of IFNα, IP-10, IL-6, and CCL2 24 h post-immunization assessed by bead-based Luminex. (*n* = 4/group) (**B**) Gating pathway for neutrophils (CD45^+^Ly6G^+^) and Ly6C^+^ monocytes (CD45^+^Ly6G^-^Siglec-F^-^Ly6C^+^CD11b^+^) in nasal tissue and quadriceps muscle. (**C**) Gating pathway for resident DCs (CD3/CD19^-^MHC-II^mid^CD11c^+^), migratory DCs (CD3/CD19^-^MHC-II^high^CD11c^+^) and Ly6C+ monocytes (CD3/CD19^-^MHC-II^mid^CD11c^-^Ly6C^+^CD11b^+^) in draining lymph nodes. Resident DCs, migratory DCs, and Ly6C^+^ monocytes frequencies in live singlet cells and CD86 median fluorescent expression in each subset in cervical lymph nodes (**D**) and inguinal lymph nodes (**E**) assessed by flow cytometry. (*n* = 4/immunized groups, *n* = 3/group PBS controls) Graphs representative of mean ± SEM with significance determined by one-way ANOVA with post-hoc Tukey’s multiple comparison test. *P < 0.05, **P < 0.01, ***P < 0.001, ****P < 0.0001 shown for select groups (full statistical analysis with exact *P* values provided in Dataset EV1).

To determine whether the long-lasting impacts of the prime immunization on shaping subsequent booster responses were due to differences in vaccine immunogenicity, and/or to innate response differences between previously primed hosts vs. naive hosts, we examined acute phase responses in the serum and tissue (at the sites of immunization). To best discern differences in innate responses, acute responses were assessed after only prime/boost (p/b) immunizations (4-week interval), and tissues were harvested 24 h post-boost. Mice were primed with IM mRNA and boosted with IM mRNA or IN NE/IVT/S or primed with IN NE/IVT/S and boosted with IM mRNA or IN NE/IVT/S. Responses were also compared to singular IM mRNA or IN NE/IVT/S priming only. IM mRNA as a singular prime alone (IN PBS; IM mRNA) or as a boost induced high levels of serum IFNα, IP-10, and IL-6 (Fig. 6A) 8 h post-immunization, which dropped at 24 h (Fig. EV4A) in agreement with previous reports (Li et al, 2022; Castaño et al, 2025). Intriguingly, the induced acute serum IFNα, IP-10, and IL-6 levels were higher in response to IM mRNA when given as a singular prime alone as compared to given as a booster dose to either IM mRNA or IN NE/IVT/S primed animals. Furthermore, IM mRNA (prime or p/b) immunization induced high levels of CCL2 (Fig. 6A), which remained elevated out to 24 h (Fig. EV4A), suggesting that IM mRNA immunization induced strong monocyte chemoattraction. While IN NE/IVT/S immunization induced much lower systemic IFNα, IP-10, and CCL2, it induced IL-6 to roughly equivalent levels across groups regardless of prime history.

Gene expression within the local tissue at the sites of immunization further demonstrated the systemic effects of IM mRNA vs. the local mucosal effects of IN NE/IVT/S. IN NE/IVT/S either as a singular prime alone or as a booster enhanced IFN-I responses within the nasal tissue as evidenced by upregulation of the interferon-stimulated genes (ISGs), *ISG15, Oas1a*, and *Mx1* (Fig. 6B). Intriguingly, while markedly lower than with IN NE/IVT/S immunization, IM mRNA immunization either as a singular prime or as a booster also resulted in detectable ISG induction within the nasal mucosal tissue in comparison to PBS control. Furthermore, only IN NE/IVT/S immunization (as a singular prime or as a boost) was able to induce nasal tissue expression of *Il1b* (in line with the Th17 inducing properties of IN NE/IVT), *Tnf* and *Ccl2* (Fig. 6B) resulting in increased frequencies of neutrophils and IFN-I responsive inflammatory Ly6C^+^ monocytes (Seo et al, 2011) (gating pathway in Fig. EV4B) recruited to the nasal mucosal tissue (Fig. 6D). Notably, heterologous IN NE/IVT/S boosting of IM mRNA experienced mice resulted in enhanced Ly6C^+^monocyte recruitment to the nasal mucosa than IN NE/IVT/S p/b, and markedly higher recruitment of Ly6C^+^monocytes to the nasal mucosa than the reverse IN NE/IVT/S→IM mRNA sequence. Intriguingly, IN NE/IVT/S immunization, regardless of whether in naive or immune-experienced mice, resulted in enhanced MHCII expression on Ly6C^+^ monocytes (Fig. 6D), which may reflect a heightened capacity for antigen presentation within the mucosal lymphoid tissue. In contrast, IN NE/IVT/S→IM mRNA did not induce increased MHCII expression on Ly6C^+^ monocytes in the nasal mucosa, highlighting the differences in the recruited cellular milieu depending on regimen sequence. While these results may be influenced to a degree by the different temporal proximity of the IN vaccination component in these regimens to the time of analysis for the IN prime-IM boost vs. IM prime-IN boost groups, comparing the IM mRNA→IN NE/IVT/S group to the IN NE/IVT/S p/b groups (which received the IN NE/IVT/S booster at the same time point) shows higher Ly6C^+^ monocyte recruitment to the nasal tissue in the IM mRNA→IN NE/IVT/S group, demonstrating that IM mRNA priming enhances recruitment to the mucosa in response to the IN booster compared to animals receiving IN NE/IVT/S priming. Similarly, the singular IN NE/IVT/S immunized group which also received the IN immunization at the same time as the IM mRNA→IN NE/IVT/S and IN NE/IVT/S→IN NE/IVT/S groups, showed lower levels of cytokines in the nasal tissue (Fig. 6B), demonstrating the impact of previously established immunity on the overall immune outcomes and that these effects are not simply due to the timing of assessment. Within the quadriceps muscle, we were also able to detect strong upregulation of *ISG15*, *Oas1a*, and *Mx1* with IM mRNA immunization (Fig. 6C). In contrast, IN NE/IVT/S immunization did not induce an IFN-I response within the quadriceps. These results support that IM mRNA is capable of altering the inflammatory signals both locally (quadriceps) and systemically (serum+nasal tissue), while IN NE/IVT/S primarily acts locally (nasal mucosal tissue). Notably, at this time point, IM mRNA (as well as IN NE/IVT/S) immunizations did not induce significant *Il1b* or *Tnf* in the muscle (Fig. 6C), consistent with the lack of neutrophil recruitment into the muscle for any of the immunization regimens (Fig. 6E). Similarly, only IM mRNA p/b showed any detectable enhancement of *Ccl2* expression in the muscle, but this enhancement was minimal compared to that observed with IN NE/IVT/S in the nasal tissue. Accordingly, there was little (only IM mRNA p/b) or no induction of Ly6C^+^ monocyte recruitment by these regimens into the muscle. MHCII expression on recruited Ly6C^+^ monocytes, while variable within groups due to the low number of monocytes present, was also minimally changed between immunization regimens in the muscle (Fig. 6E).

We next assessed the draining lymph nodes to characterize alterations between immunization regimens. Within the cLN (gating pathway in Fig. EV4C), while the frequency of resident DCs remained largely unchanged between immunization regimens, IM mRNA immunization increased the frequency of migratory DCs. Intriguingly, IM mRNA→IN NE/IVT/S had increased frequency of Ly6C^+^ monocytes, similar to the nasal mucosal tissue (Fig. EV4D). However, we noted that IM mRNA immunization substantially increased CD86 expression in resident DCs in the cLN, with the highest expression in IM mRNA primed and IM prime/boosted mice. Examination of CD86 expression on migratory DCs and Ly6C^+^ monocytes showed minimal differences between immunization regimens (Fig. EV4D). These results further support the IM mRNA immunization primes distal lymph nodes for responses to antigen re-exposure. Within the inguinal lymph node (iLN), the strong immunogenicity of IM mRNA immunization increased the frequency of migratory DCs and Ly6C^+^ monocytes compared with IN NE/IVT/S immunized mice and controls, whereas resident DC numbers had slight changes in frequency between immunization regimens (Fig. EV4E). In addition, IM mRNA immunization increased CD86 expression in resident DCs, migratory DCs, and Ly6C^+^ monocytes in comparison to IN NE/IVT/S and controls, irrespective of prior antigen exposure (Fig. EV4E). Taken together, these results suggest that the reason sequence of immunization plays such a key role in shaping the immune outcomes is that IM mRNA has the ability to enhance ISG gene expression both locally and systemically which primes resident cells within the draining lymph nodes to respond to further boost immunizations within the mucosa, while IN NE/IVT/S primarily enhances local inflammatory gene expression and infiltrating cells within the mucosa and the associated network of draining lymph nodes.

**Figure EV5:**
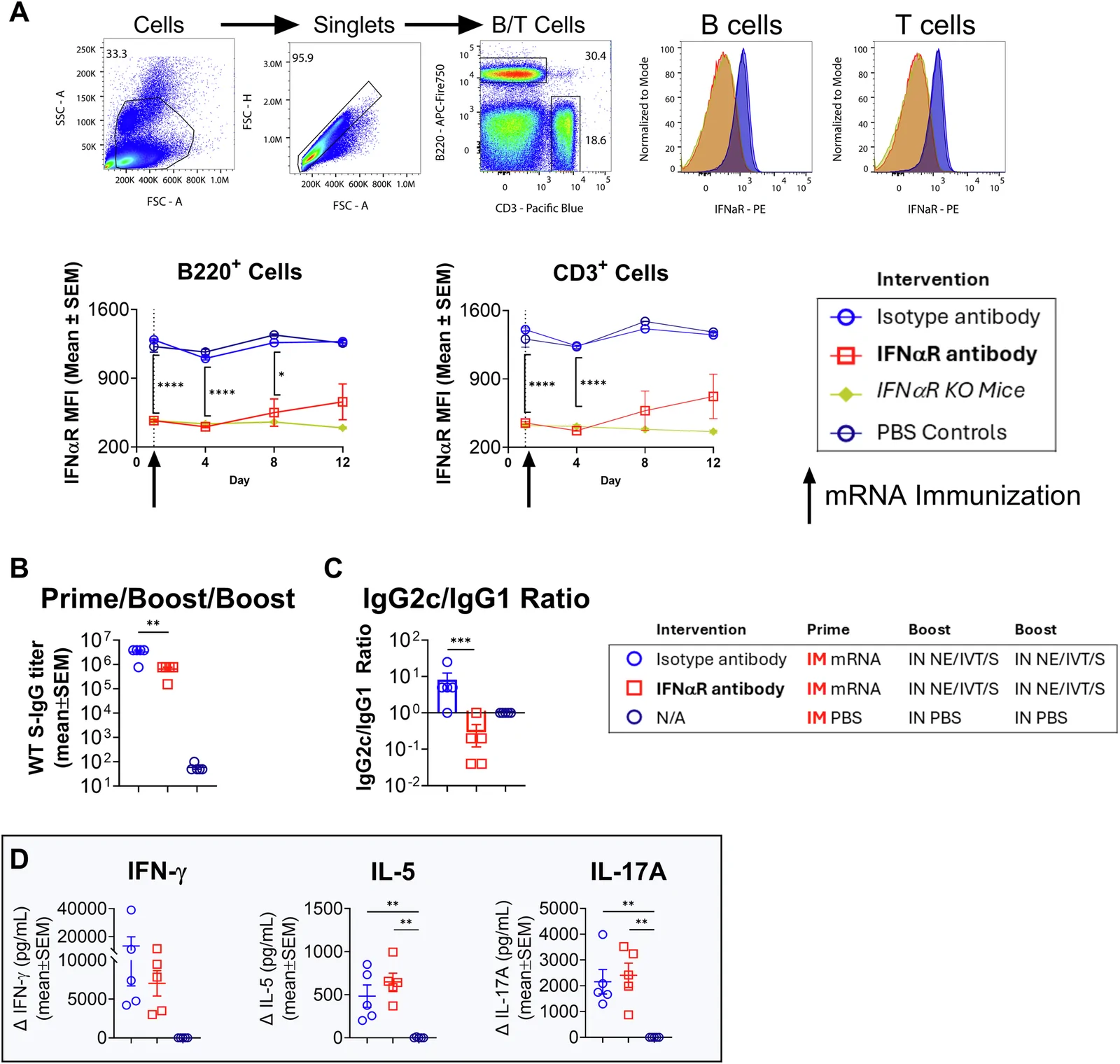
IFNαR blocking at IM mRNA prime immunization affects antibody class switching but does not affect cytokine response in the spleen. IFNαR expression was blocked with an antibody at prime immunization, and C57BL/6J mice were subsequently immunized in a prime/boost/boost schedule at 4-week intervals (Scheme in Fig. 6F). (**A**) Flow cytometry gating for determination of IFNαR surface cellular expression and mean fluorescent surface expression on B and T cells over time, assessed by flow cytometry. (*n* = 4–10/groups at day 1, *n* = 3-4/groups at days 4–12) (**B**) Serum WT-S IgG levels 6 weeks post prime/boost/boost assessed by ELISA. (**C**) Serum WT-S IgG2c/IgG1 ratios 6 weeks post-prime/boost/boost assessed by ELISA. Cytokine production in supernatants from S-stimulated spleen cells (**D**) after 3 days was measured by bead-based Luminex assay and normalized to media controls. (*n* = 5/group) Graphs representative of mean ± SEM with titers log10-transformed prior to statistical analysis. Statistical differences assessed by mixed-effects analysis with post-hoc Tukey’s multiple comparison test (IFNαR expression) or one-way ANOVA with post-hoc Tukey’s multiple comparison test. *P < 0.05, **P < 0.01, ***P < 0.001, ****P < 0.0001 shown for select groups (full statistical analysis with exact *P* values provided in Dataset EV1).

Due to the ability of IM mRNA immunization to enhance ISG expression and upregulate CD86 expression both locally and systemically, we examined whether the initial IFN-I signaling during the priming IM mRNA immunization contributed to the synergistic enhancement of mucosal and systemic immunity observed with heterologous IM mRNA→IN NE/IVT/S immunization. Accordingly, transient antibody blockade of IFNαR signaling was performed during only the IM mRNA prime in the heterologous IM mRNA; IN NE/IVT/S; IN NE/IVT/S p/b/b regimen (Fig. 6F). Complete transient blockade of IFNαR was confirmed out to 8 days, after which point blockage was reversed on B and T cells (Fig. EV5A). The lack of IFN-I signaling during IM mRNA priming slightly reduced overall S-specific IgG serum levels measured after the final-boost immunization (Fig. EV5B) but substantially altered the IgG2c/IgG1 ratio, with the lack of IFN-I signaling during priming resulting in greater IgG1 skewing (Fig. EV5C). This suggests that IFN-I induced by IM mRNA priming significantly shapes antibody class switching, which is then reinforced by subsequent boosting. Furthermore, blocking IFN-I signaling during IM mRNA priming reduced BALF S-specific IgA responses (Fig. 6G), and prevented the induction of S-specific CD8^+^ T_RM_s cells within the lungs (Fig. 6H) and NALT (Fig. 6I), further confirming the necessity of initial systemic IFN-I signaling during priming for optimal enhancement of mucosal immune responses in heterologous route immunization. Furthermore, IFNαR transient blockade abrogated S-specific IFN-γ and IL-17A responses within the cLN with the IM mRNA→IN NE/IVT/S regimen, while dramatically enhancing IL-5 expression, highlighting the dominant role IFN-I plays during IM mRNA priming in shaping downstream Th polarization in the mucosa (Fig. 6J). In the lungs, similar trends observed in the cLN remained with IFNαR blocking, shifting the Th polarization towards Th2 (Fig. 6K). Intriguingly, blocking IFNαR during IM mRNA priming had minimal effects on the heterologous prime-pull Th polarization profile within the spleen as seen in the S-specific IFN-γ, IL-5, and IL-17A cytokine response (Fig. EV5D). These results demonstrate the critical importance of strong systemic IFN-I induction during priming in heterologous prime-pull immunization regimens for establishing an immune environment that fosters enhanced responses to subsequent mucosal boosters. In other words, strong IFN-I induction has to precede a mucosal booster in order to result in “remodeling” which facilitates later enhanced downstream responses to mucosal adjuvanted vaccination. In contrast, in the reverse heterologous sequence, IN NE/IVT/S priming does not induce a robust systemic IFN-I response, which results in reduced inflammatory myeloid cell recruitment to the mucosa upon systemic mRNA boosting, leading to altered downstream immune profiles and, most notably, suboptimal mucosal immune response induction.

### Robust mucosal immunity established through initial heterologous prime-pull vaccination is amenable to delayed boosting through parenteral or mucosal routes

**Figure 7:**
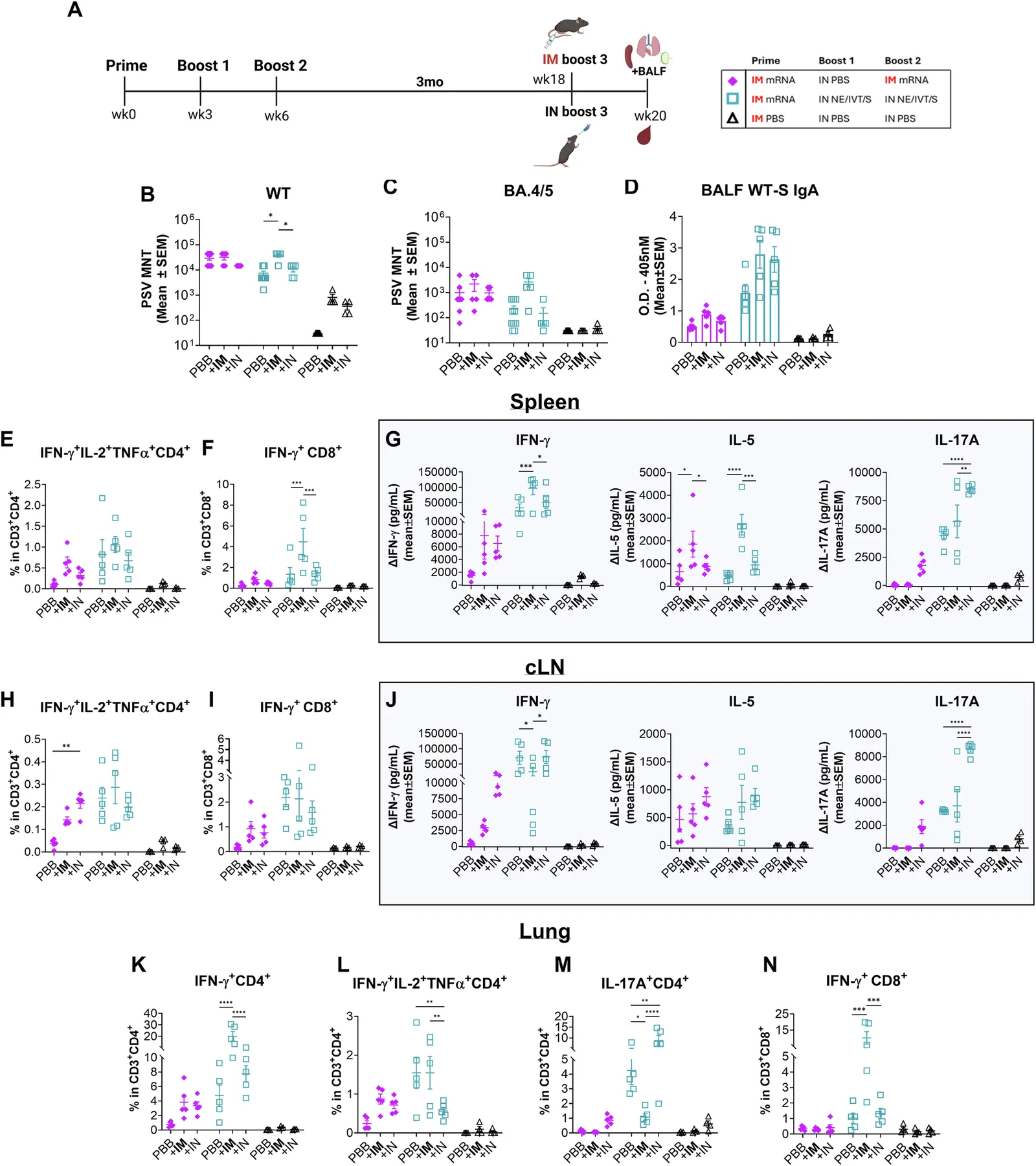
Heterologous prime/pull immunization is more amenable to delayed boosting regardless of route, while delayed mucosal boosting is limited in prior homologous IM mRNA immunization. (**A**) C57BL/6 J mice were immunized with a prime/boost/boost schedule at 3-week intervals. Immune responses were evaluated 3 months post-final boost of the initial prime/boost/boost regimen with no further treatment (PBB). Other mice receiving this same initial regimen were given a fourth immunization 3 months (week 18) after the last immunization of the initial regimen consisting of either IM mRNA (+**IM**) or IN NE/IVT/S (+IN) to evaluate the boost effect at this late time point. Immune responses were profiled from non-boosted PBB mice at week 18 and from further boosted mice at week 20. Serum nAb titers to (**B**) WT and (**C**) BA.4/BA.5 variants measured by PSV microneutralization assays (*n* = 5–10/immunized groups, *n* = 4–8/group PBS controls). (**D**) BALF WT S-specific IgA assessed by ELISA (*n* = 5/immunized groups, *n *= 4/group PBS controls). Single cell suspensions of spleens, cLNs, and lungs harvested from vaccinated mice were stimulated with 25 μg/mL SARS-CoV-2 WT S protein and profiled for cytokine expression. Frequency of cytokine-expressing (**E**) CD4^+^ T cells and (**F**) CD8^+^ T cells in spleen after 24 h stimulation assessed by flow cytometry. (**G**) Cytokine production in supernatants from S-stimulated spleen cells after 3 days was measured by bead-based Luminex assay and normalized to media controls. Frequency of cytokine-expressing (**H**) CD4^+^ T cells and (**I**) CD8^+^ T cells in cLN after 24 h stimulation assessed by flow cytometry. (**J**) Cytokine production in supernatants from S-stimulated cLN cells after 3 days was measured by bead-based Luminex assay and normalized to media controls. Frequency of cytokine-expressing (**K**–**M**) CD4^+^ T cells and (**N**) CD8^+^ T cells in lungs after 24 h stimulation assessed by flow cytometry. (*n* = 5 immunized groups, *n* = 4 PBS controls). Graphs representative of mean ± SEM with significance of boosting effect determined by two-way ANOVA or mixed-effects model with post-hoc Tukey’s multiple comparison test. *P < 0.05, **P < 0.01, ***P < 0.001, ****P < 0.0001 shown for select groups (Full statistical analysis with exact *P* values provided in Dataset EV1). Source data are available online for this figure.

We next sought to determine whether mucosal immunity could still be enhanced by heterologous mucosal boosting after a long interval. To this end, homologous IM mRNA and heterologous IM mRNA→IN NE/IVT/S immunized mice were further boosted at week 18 (3 months since the most proximal immunization), and immune responses were assessed at week 20 (Fig. 7A). Further IM mRNA or IN NE/IVT/S boosting had minimal effect on serum nAb titers to WT (Fig. 7B) or BA.4/5 (Fig. 7C) variants, with only a slight increase seen with IM mRNA boosting of the IM mRNA→IN NE/IVT/S group, suggesting an upper limit had already been reached. Importantly, for IM mRNA→IN NE/IVT/S immunized mice, additional boosting with either IM mRNA or IN NE/IVT/S both resulted in an equivalent degree of further enhancement in BALF WT-S specific-IgA (Fig. 7D), suggesting that initial establishment of strong mucosal B-cell responses enables further boosting of these responses through either parenteral or mucosal routes. In contrast, boosting the homologous IM mRNA group with IN NE/IVT/S 3 months later had no impact on BALF antigen-specific IgA, demonstrating the necessity of performing mucosal boosting more proximally in time to the systemic prime for optimal mucosal antibody induction.

As homologous IM mRNA and IN NE/IVT/S induce distinct T cell polarizations and localizations of these responses, we assessed how delayed additional boosting would bias pre-established T cell responses and alter their localization. We focused on the homologous IM mRNA and heterologous IM mRNA→IN NE/IVT/S groups. In the spleen, further IM mRNA boosting enhanced the frequencies of IFN-γ^+^− (Appendix Fig. S4A) and polyfunctional IFN-γ^+^IL-2^+^TNFα^+^- (Fig. 7E) CD4^+^ T cells in both homologous and heterologous groups; however, IFN-γ^+^ CD4^+^ T cells were substantially more enhanced in heterologous prime-pulled mice. Similarly, IL-17A^+^- (Appendix Fig. S4B) CD4^+^ T cells in the spleen were enhanced only with IN NE/IVT/S boosting, with significant enhancement found especially in the heterologous IM mRNA→IN NE/IVT/S group. Furthermore, additional IM mRNA boosting enhanced the frequency of IFN-γ^+^- (Fig. 7F) CD8^+^ T cells in the spleen, with the largest increases observed in the heterologous group. T cell response biases characterizing each boost immunization were further confirmed upon profiling secreted cytokines after antigen stimulation. Elevated IFN-γ (Fig. 7G) production observed in splenocytes from the IM mRNA→IN NE/IVT/S group was even further enhanced by additional delayed boosting, especially with IM mRNA. Moreover, boosting homologous IM mRNA-immunized mice at this late time point through either IN or IM routes restored significant IFN-γ (Fig. 7G) production in the spleen. IL-2 and TNFα (Appendix Fig. S4C) remained high but were largely unchanged with further boosting in previously immunized mice; however, IL-10 (Appendix Fig. S4C) production in the spleen was further enhanced regardless of immunization type and route. While the additional IM mRNA boost enhanced Th1 cytokines, especially for the heterologous prime-pull regimen, it also altered the pre-established cellular cytokine profile, resulting in increased production of the Th2 cytokine, IL-5 (Fig. 7G) in both homologous IM mRNA and heterologous prime-pull groups. Similarly, in addition to enhancing Th1 responses, delayed IN NE/IVT/S boosting enhanced IL-17A (Fig. 7G) in the spleen regardless of the initial immunization regimen, although the heterologous group showed the largest enhancement after the delayed boost. Thus, while foundational responses established by initial heterologous prime-pull immunization regimens in the spleen are most amenable to late boosting, boost immunizations can alter previously established Th polarization profiles dependent upon boost immunization modality and route.

Despite mucosal boosting after a prolonged interval generating minimal mucosal IgA in homologous IM mRNA mice, delayed IN NE/IVT/S boosting of homologous IM mRNA or heterologous IM mRNA→IN NE/IVT/S groups increased the frequencies of IFN-γ^+^- (Appendix Fig. S4D), IFN-γ^+^IL-2^+^TNFα^+^- (Fig. 7H), and IL-17A^+^- (Appendix Fig. S4E) CD4^+^ T cells in the cLNs, demonstrating successful establishment of mucosal Th1/Th17 responses in the homologous IM mRNA group. While cytokine-expressing CD4^+^ T cells in the cLNs were minimally impacted by delayed IM mRNA boosting, both delayed IM and IN boosts enhanced IFN-γ^+^- (Fig. 7I) CD8^+^ T cells in homologous IM mRNA mice, but minimally altered the already high levels of IFN-γ^+^ CD8^+^ T cells (Fig. 7I) in heterologous prime-pulled mice. Profiling antigen-specific cytokine secretion confirmed the significant effect each boost had on pre-established cLN immunity. In homologous IM mRNA mice, IN NE/IVT/S boosting, even after the prolonged interval, enhanced IFN-γ production within the cLNs (Fig. 7J), while IL-2 and TNFα were largely unchanged (Appendix Fig. S4F). Furthermore, while delayed IN NE/IVT/S boosting of the homologous IM mRNA group did not enhance IL-5 in the cLNs (Fig. 7J), it substantially enhanced IL-10 (Appendix Fig. S4F) and drove enhanced Th17 responses (Fig.7J). In contrast, delayed IM mRNA boosting had a minimal effect in IM mRNA-immunized mice. Within heterologous prime-pulled mice, delayed IM mRNA boosting diminished IFN-γ (Fig. 7J), IL-2, and TNFα (Appendix Fig. S4F) production in the cLNs, while IN NE/IVT/S boosting had minimal effect on these cytokines, which remained at high levels, except for a reduction in IL-2, which may be due to the recency in antigen exposure (in delayed boost groups), leading to less need for the proliferative cytokine. As in homologous IM mRNA mice, IN NE/IVT/S boosting of the heterologous prime-pull group did not affect IL-5 (Fig. 7J); however, IN NE/IVT/S boosting enhanced IL-10 (Appendix Fig. S4F) and IL-17A (Fig. 7J) in the cLN. Thus, delayed mucosal boosting establishes Th1/Th17 immunity in the mucosal dLNs of homologous parenterally immunized mice and enhances these responses in heterologous prime-pulled mice.

Within the lungs, additional IM mRNA boosting further magnified the enhanced Th1 response induced by the heterologous prime-pull immunization regimen, but decreased the Th17 profile, as evidenced by increased IFN-γ^+^- (Fig. 7K), maintained IFN-γ^+^IL-2^+^TNFα^+^- (Fig. 7L), and decreased IL-17A^+^- (Fig. 7M) CD4^+^ T cell frequencies. IN NE/IVT/S boosting also enhanced IFN-γ^+^- CD4^+^ T cell frequencies in the lungs for the heterologous group. Notably, additional delayed IM mRNA boosting markedly enhanced the frequency of IFN-γ^+^- (Fig. 7N) CD8^+^ T cells within the lungs of prime-pull immunized mice, but not in the homologous IM mRNA group. IN NE/IVT/S boosting had less impact. These results are in line with the observations above that upon establishment of strong mucosal immune responses initially, optimal further boosting of these responses within the mucosa can subsequently occur through either parenteral or mucosal routes. Overall, these results show that both homologous and heterologous immunization regimens are responsive to further boosting after an extended period, with heterologous immunization regimens being most amenable to further boosting. Additionally, varying the route of booster administration can refine both the distribution and skewing of the induced immune responses.

## Discussion

We and others have striven to emulate and enhance the mucosal immune profiles imparted by hybrid immunity using heterologous parenteral prime-mucosally delivered pull immunization regimens (Laghlali et al, 2024; Mao et al, 2022; Shin and Iwasaki, 2012). While the concept of heterologous immunization has been explored across various vaccine platforms, much mechanistic information regarding how previously established immune experience impacts downstream responses to subsequent heterologous vaccinations remains poorly understood. Moreover, while a very limited number of studies have examined the influence of the chronological sequence of exposure to antigen based on vaccine modality (Park et al, 2003) and heterologous routes (Dong et al, 2024a; McCluskie et al, 2002) on the overall immune outcomes, mechanistic insight into how vaccines synergize is lacking (Lu, 2009). Here, we have systematically investigated how the modality of initial antigen priming-including vaccine type and route of administration (IN or IM)-establishes the host immune baseline and influences the ability of subsequent booster immunizations to shape the mucosal and systemic cellular and humoral immune profile. Utilizing combinations of the IM-administered mRNA vaccine with our IN-administered NE/IVT adjuvanted subunit vaccine, we demonstrated that heterologous immunization strategies induce enhanced and more multifaceted immunity with markedly improved durability compared to homologous regimens. Importantly, optimal mucosal responses and Th bias were strictly dependent upon the sequence of immunization, with the initial antigen exposure route and vaccine type having a profound effect on subsequent boost vaccination immunogenicity. By profiling local and systemic acute innate responses induced by each vaccine, major differences between the responses to IM mRNA and IN NE/IVT/S vaccines were identified in naive hosts. Moreover, profiling acute responses to vaccination in the context of a booster in immune-experienced hosts demonstrated differential responses to the boost depending on the mode of initial immune priming, with key innate responses induced specifically during priming being necessary for promoting enhanced responses to later mucosal vaccinations. These findings underscore the fundamental distinctions between vaccination in naive vs. immune-experienced hosts, with important implications both for vaccine regimen design and for understanding how mucosal infection shapes subsequent vaccine responses.

Serum antigen-specific IgG titers and nAb breadth were less affected by the chronological sequence of antigen exposure in heterologous regimens with IM mRNA and IN NE/IVT/S than T cell responses, with the most notable change being a shift in IgG2c skewing with mucosal priming vs. systemic priming. This may be partially explained by the absence of systemic IFN-I induction with IN NE/IVT/S, as IFN-I amplifies IgG2c germinal center B cell responses (Dahlgren et al, 2022). However, the chronological sequence of antigen exposure had a profound impact on mucosal IgA induction, with parenteral priming followed by mucosal boosting resulting in significant enhancements compared to the homologous IM mRNA regimen, whereas mucosal priming followed by parenteral boost gave minimal/no enhancement. The importance of the administration sequence was further confirmed upon measuring antigen-specific T cell responses and profiling localization within various immune compartments. Both heterologous (IM mRNA with IN NE/IVT/S) and homologous IM mRNA regimens induced robust CD4^+^ and CD8^+^ T cell responses systemically; however, the major benefit of the heterologous regimen was within the mucosal compartment. Within homologous regimens, vaccine route and modality shaped T cell responses and localization: IM mRNA mainly induced splenic CD8^+^-dominant responses, while IN NE/IVT/S predominantly induced cLN-localized CD4^+^-biased responses. Heterologous regimens with IM mRNA and IN NE/IVT/S generated equally robust S-specific CD8^+^ T cell responses in the spleen and cLN irrespective of sequence. However, frequencies were not significantly enhanced relative to homologous IM mRNA. Similarly, heterologous IM mRNA→IN NE/IVT/S and homologous IM mRNA both induced strong antigen-specific CD4^+^ T cells in the spleen. In contrast, only heterologous IM mRNA→IN NE/IVT/S dramatically enhanced S-specific CD4^+^ T cells within the cLN, while no detectable levels were induced by homologous IM mRNA immunization. Importantly, we observed a deficiency in generating S-specific CD4^+^ T cells within the spleen and cLN with the reverse IN NE/IVT/S→IM mRNA heterologous sequence. Mucosal tissue profiling further demonstrated the importance of administration sequence, as IM mRNA→IN NE/IVT/S was much more effective in generating antigen-specific CD8^+^ T_RM_s within the lungs and NALT than homologous IM mRNA, whereas strikingly, no enhancement was observed with the reverse mucosal prime; systemic boost regimen. Thus, optimal vaccine-induced mucosal responses are initiated systemically and boosted mucosally. The WT S used for these studies was the less immunogenic S-2P version, such that differences between regimens could be better observed, and the S protein version encoded by the mRNA vaccine would match (Hsieh et al, 2020). Antigen doses are accordingly generally higher for IN administration with this version compared to the significantly more immunogenic Hexapro version of WT S used in some studies with other vaccine platforms (Yang et al, 2024; Lu et al, 2022; Mao et al, 2022). Additionally, direct comparisons across these studies are difficult due to the use of larger administration volumes, which do not limit vaccine delivery to the nasal cavity as we have herein (Pan et al, 2026; Mao et al, 2022).

Vaccine efficacy relies on achieving optimal T helper cell biases and is most often pursued through tailoring innate activation signals by adjuvant selection (Waris et al, 1996; Lavelle and McEntee, 2024). However, less focus is placed on how the initial antigen exposure route and inflammatory signals shape subsequent booster immunogenicity. Responses established during priming alter the immune environment present during subsequent antigen exposures, which can synergize or interfere with secondary responses to the same or even unrelated antigen (Dong et al, 2024b; Chen et al, 2025). We therefore assessed how the chronological sequence of antigen exposure could be strategically leveraged to induce optimal antiviral polarization profiles systemically and within the mucosa. Homologous IM mRNA or IN NE/IVT/S immunization induced distinct T cell polarization profiles and localization. While homologous IM mRNA imparted a Th1/Th2 response in the spleen, there was a notably absent IFN-γ response with concomitantly elevated Th2 cytokines in the cLN. In contrast, homologous IN NE/IVT/S imparted a Th1/Th17 response in the spleen and a Th17 response in the cLN. In heterologous immunization regimens, the Th bias and localization established during the priming encounter with antigen had a considerable impact on subsequent boost immunizations. IN NE/IVT/S boosting of IM mRNA-established immunity significantly enhanced the Th1 cytokine profile and imparted Th17 responses in both spleen and cLN, whereas IM mRNA boosting of IN NE/IVT/S-established immunity yielded notably elevated Th2 and depressed Th1/Th17 responses in the spleen and absent Th1 responses in the cLN. Furthermore, heterologous IM mRNA→IN NE/IVT/S uniquely induced a marked enhancement in polyfunctional CD4^+^ T cells both systemically and mucosally. This is highly significant given that such polyfunctional CD4^+^T cells as well as Th1/Th17 responses have been consistently identified as strong predictors of protection across multiple respiratory viral pathogens (Seder et al, 2008; Kandasamy et al, 2016; Pizzolla et al, 2018).

The importance of the sequence of antigen exposure was further confirmed in a cross-variant lethal challenge with B.1.351 in vaccinated K18-hACE2 mice. Despite robust protection afforded by all heterologous vaccination groups tested as well as homologous IM mRNA, cytokine profiling within the lungs post-challenge, even in protected mice, revealed significant distinctions in the type of response to infection between different immunization sequences with the same vaccines. Thus, it is likely that distinct cellular mediators contribute to the protection imparted by the different sequences of heterologous immunization. While distinctions in the host response to viral challenge between vaccination regimens are clear, clinical differences may be more pronounced in the absence of such high nAbs, as when antibodies wane or as escape variants emerge. Further, as nAbs have been shown to afford disproportionately more protection against challenge in mouse models than in humans, future studies evaluating responses in B-cell-deficient models will provide valuable information.

To define the mechanisms underlying the marked differences in T cell polarization and localization resulting from simply reversing the immunization order, we characterized acute innate responses elicited by each vaccine type locally within the tissue at the sites of immunization (muscle vs. nasal mucosa) and systemically. IM mRNA induced a systemic IFN-I response, enhancing ISG expression both locally within the muscle and distally in the nasal tissue, while IN NE/IVT/S induced only a local, but robust ISG response within the nasal mucosal tissue. Accordingly, marked differences in the immune cell infiltrates were observed within the tissue, with IN NE/IVT/S driving neutrophil and MHCII^+^Ly6C^+^monocyte infiltration into the nasal mucosa, while IM mRNA induced minimal infiltration of these cell types in the muscle. Most notably, prior IM mRNA vaccination promoted greater accumulation of MHCII^+^ expressing Ly6C^+^monocytes within the nasal mucosa upon IN NE/IVT/S boosting compared with IN NE/IVT/S immunization in naive animals. In contrast, MHCII^+^ expressing Ly6C^+^monocytes were markedly reduced with the reverse IN NE/IVT/S→IM mRNA. These results support greater antigen-presentation potential in the mucosa and suggest that prior IM mRNA vaccination conditions the mucosal environment to promote innate cell recruitment and activation to foster the enhanced local CD4^+^T cell responses observed. Further illustrating the local and systemic effects of IM mRNA immunization, IM mRNA enhanced DC responses within the cLN and iLN, while IN NE/IVT/S enhanced responses primarily within the cLN. Notably, priming with IN NE/IVT/S before IM mRNA boosting diminished the serum IFN-I response to IM mRNA, resulting in lower DC responses within the cLN. Thus, IM mRNA immunization establishes both local and systemic conditioning that enables synergy with subsequent mucosal NE/IVT/S boosting, whereas mucosal priming fails to induce comparable conditioning when the sequence is reversed.

The importance of IFN-I in generating mucosal immune responses in heterologous immunization was confirmed by transiently blocking IFNαR only during IM mRNA priming in heterologous prime-pull immunization sequences. The lack of IFN-I signaling during the prime resulted in significantly diminished mucosal IgA, CD8^+^ T_RM_ T cells within the lung and NALT, and differentially polarized responses within the lung and cLN with diminished Th1/Th17 responses and upregulated Th2 responses. Notably, transient IFNαR blockade during IM mRNA priming resulted in minimal changes to T cell responses within the spleen, suggesting IFN-I at IM mRNA prime plays a significant role in priming the mucosa to further exposure to antigen at mucosal surfaces. Given the critical role of IFN-I induction we found during priming, it would be valuable to examine the impact of co-parenteral administration of IFN-I during priming with currently licensed injectable vaccines to determine whether this similarly conditions enhanced mucosal responses to intranasal vaccinations. IFN-I induction by both the BNT162b2 mRNA vaccine as well as an AS03 adjuvanted H5N1 vaccine have been shown in human vaccinees to induce an innate memory-like state through epigenetic reprogramming which leads to heightened responses to booster parenteral vaccinations (Arunachalam et al, 2021; Cortese et al, 2025). It is likely that such training induced by IFN-I also plays a major role in shaping responses in the mucosa that we observe here as well. Further investigation is currently underway to investigate differences in epigenetic signatures induced by the regimens in this study both systemically and within the mucosa. Other related factors besides IFN-I likely also influence the optimal mucosal immune outcomes observed with the IM mRNA→IN NE/IVT/S, including the strong induction of IFN-γ by the IM mRNA which is markedly amplified by NE/IVT/S. IFN-Is and IFN-γ are tightly intertwined, with IFN-Is strongly upregulating production of IFN-γ. IFN-I induction can also epigenetically reprogram T cells, priming them to be more capable of secreting larger amounts of IFN-γ in response to a booster. Given that rapid local IFN-γ production by Th1 memory cells during a boost can facilitate recruitment of circulating immune cells into the mucosal tissue, fortify the residency of T_RM_s in the mucosa, as well as promote formation of additional CD4⁺ T_RM_s and B_RM_s, future studies dissecting the role of IFN-I-induced IFN-γ in mediating the mucosal responses to these regimens will be valuable. Thus, fine-tuning of immune responses can be achieved through heterologous immunization regimens by selecting appropriate initial administration routes and priming vaccine platforms that will establish foundational immune environments (Th polarization, cytokine milieu) that most effectively foster tailored immune responses--especially mucosally-targeted responses—induced by later booster immunizations. The superior immune profile induced by the systemic prime, mucosal boost regimen suggests CD4^+^ T cell responses established by the prime result in a cytokine environment that promotes mucosal immune responses induced by subsequent mucosal exposure, as observed by others (Kwon et al, 2025). These results are analogous to studies demonstrating that manipulating pre-existing antigen-specific Th responses established by prior infection has a profound impact on shaping later vaccine responses (Moran et al, 1999). Furthermore, IN boosting has been shown to induce recruitment of B and T cells primed in the periphery via systemic immunization to the mucosa, leading to differentiation into IgA-secreting plasma cells and T_RM_s, respectively (Kwon et al, 2025). Thus, the IM mRNA prime→IN NE/IVT/S boost sequence optimally leverages the strong CD4^+^ and CD8^+^ T cell-inducing properties of the IM mRNA vaccine and harnesses the tailored mucosal immune-activating properties of the IN NE/IVT adjuvant to redirect them to the respiratory tract, while enhancing and further shaping these responses within the mucosa.

A major hurdle facing mRNA vaccines has been the rapid waning of immunity (Notarte et al, 2022). Assessment of longevity (3-month post-final boost) showed a clear benefit of heterologous immunization, especially IM mRNA prime→IN NE/IVT/S boost. Heterologous IM mRNA→IN NE/IVT/S resulted in highly durable serum cross-variant nAbs and antigen-specific mucosal IgA, as well as remarkably durable systemic *and* mucosal CD4^+^/CD8^+^ cellular immunity. Most notably, polyfunctional IFNγ^+^IL2^+^TNFα^+^CD4^+^ T cells, which wane quickly in human mRNA vaccine studies (Pieren et al, 2023), were further increased in both systemic and mucosal compartments in IM mRNA→IN NE/IVT/S vaccinated mice. This was in stark contrast to homologous IM mRNA vaccination, which showed rapid drops in CD4^+^/CD8^+^ responses to near baseline levels and contraction in serum nAbs during this interval. Furthermore, the enhanced Th1/Th17 polarization induced by IM mRNA→IN NE/IVT/S was maintained over time, as well as the Th2/Th17 polarization of the reverse IN NE/IVT/S→IM mRNA regimen, demonstrating the far-reaching impact of the initial immunization sequence. Thus, heterologous immunization with different vaccine platforms through different routes generates more durable immune responses than homologous immunization with significant potential for tailoring immunity through strategic design of vaccination sequences. Future characterization of distinctions in the T cells induced by heterologous immunization such as profiling the PD-1 metabolic axis (Kalia et al, 2021; Corrado and Pearce, 2022) and examining CD4-CD8 crosstalk within the lymphoid organs may yield key information that can be applied towards improving current immunization regimens.

We further assessed how delayed boosting (3 months later) of homologous IM mRNA or heterologous IM mRNA→IN NE/IVT/S vaccinated groups would reinforce or alter the immune profile. Further boosting with either an IM mRNA or IN NE/IVT/S dose had minimal effect on the already high levels of nAbs induced by these regimens, likely due to clearance of vaccine antigens by high levels of pre-existing antibodies, which was found to make boosting B (but not T) cell responses increasingly more difficult (Dangi et al, 2023). Importantly, BALF IgA in the homologous IM mRNA group was not enhanced by delayed IN NE/IVT/S boosting, demonstrating the necessity of proximal mucosal boosting for optimal induction of mucosal secretory antibody responses. In contrast, the robust mucosal IgA response generated by IM mRNA priming, IN NE/IVT/S boosting was equally enhanced by additional boosting through either IN or IM routes, suggesting that early establishment of strong mucosal responses subsequently allows for boosting mucosal responses even by IM immunization. Notably, T cell responses established through heterologous prime-pull immunization were significantly more amenable to further boosting through either IM or IN routes after this interval in comparison to homologous IM mRNA immunization. The Th bias and localization properties of the delayed boosting vaccine were imparted on the established immunity, yielding enhanced Th1/Th17 responses emphasized within the mucosal draining lymph nodes upon additional IN NE/IVT/S boosting and enhanced Th1/Th2 responses emphasized in the spleen with additional IM mRNA boosting. Interestingly, while BAL IgA was not induced by delayed mucosal boosting of IM mRNA-immunized mice, “pulling” of cellular immune responses with significant Th1/Th17 polarization to the cLNs and lungs could be detected, demonstrating that substantial mucosal cellular immunity can be achieved by mucosal boosting even with a prolonged delay and despite waning systemic immunity. Due to the extended durability and enhanced boosting response imparted by heterologous immunization regimens, further molecular characterization of the CD4^+^ T cells will likely yield important insights, especially since hybrid immunity imparts differential molecular signatures on CD4^+^ T cells than homologous mRNA immunization (Neidleman et al, 2021). These results have significant implications for the development and selection of booster vaccines against respiratory pathogens.

In summary, we show that in heterologous immunization regimens, the prime immunization plays a pivotal role in driving the formation of systemic and mucosal immunity shaped by further booster immunizations. These findings underscore the importance of assessing vaccine responses in the context of diverse types of pre-existing immunity, given that the mode and route of initial antigen exposure can result in significant differences in programming the immune system for downstream vaccine responses. We also demonstrate the superior durability of cellular and humoral responses in adjuvanted heterologous immunization regimens and that the benefits of heterologous immunization are still achievable even after long periods of delay in boosting. Thus, together, these results highlight an effective strategy for driving a more multifaceted and tailored immune profile optimized for protection against SARS-CoV-2. As the NE adjuvant has already demonstrated safety in multiple clinical trials, as have several RIG-I agonists, mucosal NE/IVT-adjuvanted subunit boosters present a highly promising platform for harnessing and redirecting systemic immune responses primed through parenteral vaccination to the mucosa (NCT01354379, NCT04148118, NCT05397119) (Stanberry et al, 2012). Overall, these findings have broad implications for improving the immune profiles of parenteral vaccines against other respiratory viruses, and demonstrate that in heterologous vaccination, vaccine type, administration route, as well as chronological sequence of vaccination are all important factors that should be leveraged advantageously in the future development of effective vaccination strategies.

## Methods

**Table:** Reagents and tools table

Reagent/resource	Reference or source	Identifier or catalog number
**Experimental models**		
C57BL/6J	The Jackson Laboratory	IMSR_JAX:000664
B6.Cg-Tg(K18-ACE2)2Prlmn/J	The Jackson Laboratory	IMSR_JAX:034860
IFNaR1^−/−^ KO mice	James Moon, PhD	IMSR_JAX:028288
**Cell models**		
HEK293T-hACE2	BEI Resources	NR-52511
Vero TMPRSS2	NIBSC	100978
Vero E6	ATCC	CRL-1586
**Virus**		
SARS-CoV-2 B.1.351	Andy Pekosz, PhD	N/A
**Antibodies**		
Flow Cytometry Antibodies	This Study	Table EV1
Goat Anti-Mouse IgG - AP	Jackson ImmunoResearch	AB_2338538
Goat Anti-Mouse IgG1 - AP	Jackson ImmunoResearch	AB_2338542
Goat Anti-Mouse IgG2c - AP	Jackson ImmunoResearch	AB_2338545
Goat Anti-Mouse IgA - AP	ThermoFisher Scientific	AB_2533952
Mouse Anti-Mouse IFNaR-1 (Clone MAR1-5A3)	Leinco Technologies	AB_2830518
Mouse IgG1 Isotype Control (Clone HKSP)	Leinco Technologies	AB_2831344
**Oligonucleotides and other sequence-based reagents**		
qPCR Primers	This Study	Table EV2
**Chemicals, enzymes, and other reagents**		
W805EC Nanoemulsion	In House	N/A
IVT	In House	N/A
BNT162b2 mRNA	Pfizer	
WT S Protein	University of Michigan Center for Structural Biology	(SARS-CoV-2 WA1/2020)
Omicron S Protein	University of Michigan Center for Structural Biology	(SARS-CoV-2 B.1.1.529)
Collagenase A	Roche	Cat #11088793001
Collagenase D	Roche	Cat #11088866001
DNase I	Roche	Cat #11284932001
Lung Dissociation Kit	Miltenyi Biotec	130-095-927
Skeletal Muscle Dissociation Kit	Miltenyi Biotec	130-098-305
Dasatinib	Sigma-Aldrich	Cat #SML2589
VTWFHAIHVSGTNGT-PE Tetramer	NIH Tetramer Core	
VNFNFNGL-PE Tetramer	NIH Tetramer Core	
eBioscience Fixable Viability Dye eFlour 520	ThermoFisher Scientific	65-0867-18
Live/Dead Fixable Aqua Dead Cell stain	ThermoFisher Scientific	L34966
Brilliant Stain Buffer	BD Biosciences	Cat #566349
RBC Lysis/Fixation Solution 10x	Biolegend	Cat #422401
eBioscience Intracellular Fixation and Permeabilization Buffer Set	ThermoFisher Scientific	Cat #88-8824-00
ProcartaPlex Mouse Cytokine and Chemokine Panel 1, 26 plex	ThermoFisher Scientific	EPX260-26088-901
ProcartaPlex Mouse custom kit	ThermoFisher Scientific	
Milliplex MAP Magnetic Mouse Cytokine/Chemokine Multiplex custom kit	EMD Millipore	
Bright-Glo Luciferase Assay System	Promega	Cat #E2650
pNPP Substrate	ThermoFisher Scientific	Cat #34045
TRIzol Reagent	ThermoFisher Scientific	Cat #15596026
PureLink RNA Mini kit	ThermoFisher Scientific	Cat #12183018 A
Maxima H Minus First Strand cDNA Synthesis kit	ThermoFisher Scientific	Cat #K1652
PowerUp SYBR Green	ThermoFisher Scientific	Cat #A25742
**Software**		
Graphpad Software Version v11	Graphpad Software LLC	
FlowJo Software V10	BD Biosciences	
R Statistical Software	https://www.R-project.org/.	
**Other**		
Novocyte 3000	Agilent Technologies	
Cytek 2 Northern Lights	Cytek Biosciences	
QuantStudio 7	ThermoFisher Scientific	

### In vivo mouse studies

For immunization and challenge studies, 6–8-week-old female C57BL/6 J or B6.Cg-Tg(K18-ACE2)2Prlmn/J mice (Jackson Laboratories) were housed in specific pathogen-free conditions within individually ventilated cages, 2–5 animals per cage. For challenge studies, mice were transferred to ABSL3 facilities 1 week prior to challenge. Food and water were provided ad libitum, and mice were housed in a 12:12 light–dark cycle. Sample size was determined based on power analysis to achieve statistical significance while minimizing animal usage. Housed mouse cages were randomly allocated for immunization regimens. Animal procedures were approved by the Institutional Animal Care and Use Committees (IACUC) at the University of Michigan (Protocol: PRO00012015) and Icahn School of Medicine at Mount Sinai (Protocol: IPROTO202200000097) and were carried out in accordance with these guidelines.

### Adjuvants and antigen

NE was produced by emulsification of cetylpyridinium chloride (CPC) and Tween80 surfactants, ethanol (200 proof), super-refined soybean oil (Croda), and purified water using a high-speed homogenizer (Wong et al, 2015). Surfactants were mixed at a 1:6 ratio of CPC/Tween80. NE formulation homogeneity of particle size (*d* = 450–550 nm) and charge (zeta potential = 50–55 mV in 1 mM HEPES pH 7.0) were confirmed through measurements using a zetasizer (Malvern Nano ZSP). Stability was evaluated >1 year. The sequence of IVT RNA has previously been described in detail (Patel et al, 2013). Briefly, IVT was in vitro transcribed from a pUC19 plasmid containing DNA encoding the IVT sequence using a HiScribe T7 High Yield RNA synthesis kit (New England Biolabs), followed by DNAseI digestion and purification using a TURBO DNA-free kit (ThermoFisher) and RNeasy purification kit (Qiagen), respectively. Recombinant WT SARS-CoV-2 (derived from Wuhan Hu-1) and OMI SARS-CoV-2 (BA.1) full-length S protein (prefusion-stabilized two-proline “S2P” spike with C-terminal His tags) were produced in ExpiCHO cells and purified by the University of Michigan Center for Structural Biology as previously described (Laghlali et al, 2024). The BNT162b2 mRNA vaccine was obtained through the NIH SAVE program. The S2P version of the S protein was selected as it matches the S protein encoded by the BNT162b2 mRNA vaccine used.

### Immunization

Immunization regimens and schedules specific to each study are described in each figure legend. Briefly, mice were anesthetized with isoflurane using an IMPAC6 precision vaporizer. For IN immunization, mice were given 12 μL (6 μL/nare) of each vaccine formulation, a volume which restricts delivery to the nasal cavity. For IM immunization, mice were administered 50 μL of vaccine in the right hind limb. For immune profiling in C57Bl/6 mice, IM vaccine doses consisted of 1.7 μg BNT162b2 mRNA, and IN vaccine doses consisted of 20 μg of WT S protein in PBS or 20% NE with 0.5 μg IVT DI (NE/IVT/S). For immunization-challenge studies in K18-hACE2 mice, IM vaccine doses were 0.4 μg BNT162b2 mRNA, and IN vaccine doses were 15 μg of WT S protein in PBS or 20% NE with 0.5 μg IVT DI (NE/IVT/S) to better observe differences in protection. Serum was obtained by saphenous vein bleeding and by cardiac puncture at the end of the experiment. Bronchial alveolar lavage fluid (BALF) was obtained by lung lavage with 0.8 mL PBS containing protease inhibitors (Roche) at the end of the experiment.

### Viral challenge and lung cytokine and immune cell analysis

SARS-CoV-2 clinical isolate B.1.351 virus (a kind gift from Dr. Andy Pekosz) was propagated in Vero E6 cells or Vero-TMPRSS2 and verified by deep sequencing. All SARS-CoV-2 viral work was performed in certified BSL3 or ABSL3 facilities in accordance with institutional safety and biosecurity procedures. Immunized K18-hACE2 mice were challenged intranasally 3 weeks post-boost (week 9) with 10^4^ PFU B.1.351 delivered in 30 μL PBS. Mice were sacrificed 3- and 5-days post infection (dpi). Lungs, nasal turbinates, and brain were harvested in 500 μL of PBS. Homogenates were prepared for virus titration by plaque assay as previously described (Rathnasinghe et al, 2020). Cytokine profiling post-challenge for both 3 and 5 dpi was performed using 25 μL of lung homogenate from challenged mice using a ProcartaPlex™ Mouse Cytokine & Chemokine Panel 1, 26 plex (Invitrogen) following the manufacturer’s instructions. Pulmonary infiltrates upon infection were characterized by dissociation of the left lung lobe with the Lung dissociation kit (Miltenyi Biotec). Single-cell suspensions were filtered through a 70-μm cell strainer, incubated with FC block in FACS buffer for 15 min at RT, prior to staining for viability with Fixable Viability Dye eFlour520 (ThermoFisher) and surface markers (Table EV1). Cells were washed in FACS buffer, fixed in IC fixation buffer for 30 m at 4 °C, permeabilized for 15 m, and then stained for intracellular expression markers (Table EV1). Data was acquired using a Cytek 2 Northern Lights spectral flow cytometer (Cytek Biosciences). Flow cytometry data were analyzed using FlowJo (BD Biosciences) or R statistical software (R Core Team, 2022) with the packages readxl, flowCore, pheatmap, RColorBrewer, matrixStats, ggplot2, reshape2, dplyr, limma, FlowSOM, ConsensusClusterPlus, and Rtsne.

### ELISA

ELISA was performed as previously described (Laghlali et al, 2024; Jangra et al, 2023). Immunograde plates (Midsci) were coated with 100 ng S protein and blocked in 5% non-fat dry milk/PBS. Serially diluted sera or BALF (diluted 1:4) samples were subsequently transferred to the plates and incubated 2 h at 37 °C followed by overnight incubation at 4 °C. Plates were washed with PBST (0.05% Tween20), and appropriate alkaline phosphatase-conjugated secondary antibodies were added and incubated 1 h at 37 °C. Plates were washed with PBST and developed with p-nitrophenyl phosphate (pNPP) substrate in diethanolamine with absorbance was measured at 405 nm. Titers were determined using a cutoff defined as the mean absorbance of naive serum at the lowest dilution plus two (for total IgG) or four (for IgG1, IgG2c) times the standard deviation. Samples with absorbance values below the cutoff were assigned a value equal to half the lowest dilution.

### Cell lines

Vero E6 and Vero-TMPRSS2 cells (ATCC) were maintained in DMEM supplemented with 10% heat inactivated fetal bovine serum (HI FBS) (Gibco) and 1× non-essential amino acids (NEAA). HEK293T cells expressing hACE2 (293T-hACE2 from BEI Resources) were maintained in HEK293T medium: (DMEM with 4 mM L-glutamine, 4500 mg/L L-glucose, 1 mM sodium pyruvate and 1.5 g/L sodium bicarbonate, 10% HI FBS, and 100 IU penicillin, and 100 μg/mL streptomycin). Cell lines were cultured in a tissue culture incubator at 37 °C with 5% CO_2_. All cell lines were authenticated and tested for mycoplasma by ATCC or BEI Resources prior to purchase and were strictly used at low passages.

### Lentivirus pseudotyped virus

Generation of pseudotyped lentiviruses (PSVs) expressing the SARS-CoV-2 S proteins from WT, B.1.351, B.1.617.2, and B.1.1.529 (BA.1), and BA.4/5 variants harboring GFP and firefly luciferase reporter genes was performed as previously described for the WT PSV (Ou et al, 2020). Plasmids carrying the full-length SARS-CoV-2 spike protein from each variant containing a C-terminal 19 amino acid deletion to remove the ER retention signal were used for pseudotyping (Invivogen). Viral titers (TU/mL) across variants were determined by measuring PSV transduction of GFP in 293T-hACE2 cells.

### Pseudovirus (PSV) microneutralization (MNT) assays

HEK293T-hACE2 cells were seeded at 1.25 × 10^4^ cells/well overnight on black tissue culture plates. In total, 50 μL of serially diluted sera from immunized mice was added to 50 μL of the respective lentiviral-based PSVs (40,000 TU/mL) in HEK293T medium and incubated for 1 h at 37 °C. The virus/serum mixture was then added to the cells and incubated for 3 d at 37 °C, after which the infection media was removed, and luminescence was measured by the addition of 25 μL Bright-Glo substrate (Promega) in 25 μL PBS. Neutralizing antibody titers were determined as the dilution at which the luminescence remained below the luminescence of the (virus only control-uninfected control)/2. Samples with no detectable neutralization activity were designated as having a titer of half the lowest dilution of the assay.

### Antigen-specific T-cell characterization

Mice were injected with 3 μg of CD45- AF488 (Clone: 30-F11; Biolegend) diluted in 100 μL of sterile PBS to discriminate circulating cells. Mice were then euthanized 3 m post injection. For T cell responses, spleens and cLNs isolated from immunized mice were mechanically dissociated and processed as previously described into single cell suspensions (Wong et al, 2015). Collagenase A (Roche) was dissolved in HBSS with Ca^2+^/Mg^2+^ at 10 mg/mL and then diluted in digestion media (RPMI-1640, 10% HI FBS, 2 mM L-glutamine, 1% NEAA, 1 mM sodium pyruvate, 100 IU penicillin, 100 μg/mL streptomycin). Lungs and NALT were minced with surgical scissors and placed in digestion media containing 1 mg/mL collagenase A with 20 U/mL of DNase-I (Roche) and incubated for 1 h at 37 °C. Suspensions were passed repeatedly through an 18-gauge needle and filtered through a 70-μm cell strainer. RBCs were removed by addition of ACK lysis buffer for 5 m at RT. All cell stimulations were performed in T cell media (DMEM, 5% HI FBS, 2 mM L-glutamine, 1% NEAA, 1 mM sodium pyruvate, 10 mM MOPS, 50 μM 2-mercaptoethanol, 100 IU penicillin, 100 μg/mL streptomycin). For Class I tetramer staining, 1 × 10^6^ cells from lungs, spleen or cLN, or the entire NALT were washed in FACS buffer (PBS, 1% FBS, 2 mM EDTA, 0.05% sodium azide), blocked with Fc block for 30 min at 4 °C, and stained for surface markers and Class I tetramer (VNFNFNGL-PE; NIH Tetramer Core; 1:250 dilution) for 1 h at 4 °C. For Class II tetramer staining, 2 × 10^6^ cells from spleen or cLN were blocked with Fc block for 30 m in T cell media containing 50 nM Dasatinib (Sigma-Aldrich) at 37 °C. Cells were then stained with Class II Tetramer (VTWFHAIHVSGTNGT-PE; NIH Tetramer Core; 1:250 dilution) for 1 h at 37 °C, washed with FACS buffer, and subsequently stained for surface markers at 4 °C for 30 min. Cells in both Class I and II tetramer staining were extensively washed in PBS/2 mM EDTA before staining with LIVE/DEAD Fixable Aqua Dead Cell Stain in PBS/2 mM EDTA for 20 m at 4 °C. Cells were washed with PBS/2 mM EDTA and fixed in IC fixation buffer. For intracellular cytokine analysis, 1 × 10^6^ cells isolated from the lungs, spleen, or cLNs were stimulated with 25 μg/mL S protein in T cell media for 24 h at 37 °C, with brefeldin A added during the last 6 h. Cells were washed and then blocked with Fc block for 10 m at 4 °C prior to staining for surface T cell markers for 30 m at 4 °C, with all steps done in FACS buffer. Cells were extensively washed in PBS/2 mM EDTA before staining with LIVE/DEAD Fixable Aqua Dead Cell Stain in PBS/2 mM EDTA for 20 m at 4 °C. Cells were washed with PBS/2 mM EDTA and fixed in IC fixation buffer. Cells were then permeabilized and stained with antibodies for intracellular cytokines (Table EV1) in permeabilization buffer for 30 m at 4 °C. Cells were washed with permeabilization buffer and resuspended in PBS/2 mM EDTA. FACS was performed on a Novocyte3000 flow cytometer and analyzed using FlowJo software. Isolated spleen and cLN cells were plated at 8 × 10^5^cells/well and stimulated with 25 μg/mL S protein in T cell media for 72 h at 37 °C. Supernatants were assessed for secreted cytokines using a Milliplex MAP Magnetic Mouse Cytokine/Chemokine multiplex immunoassay (EMD Millipore).

### Acute serum and local tissue and draining lymph node characterization

Eight hours post-immunization, mice were bled, and serum was collected. Serum was subsequently assessed for IFNα, IL-6, IP-10, and MCP-1 with a ProcartaPlex™ Mouse multiplex immunoassay kit (ThermoFisher). 24 h post-immunization, the draining lymph nodes (cervical lymph nodes for IN, right inguinal lymph node for IM), quadriceps muscle, and nasal tissue, including the NALT, were removed from mice. Draining lymph nodes were dissociated and digested in media (RPMI1640/10%FBS/100 IU penicillin/100 μg/mL streptomycin /10 mM HEPES) containing 400 Mandl U/mL collagenase D (Roche) for 1 h. Quadriceps muscles were digested with the skeletal muscle dissociation kit (Miltenyi Biotec) according to the manufacturer’s protocol. Nasal tissue was digested in a similar manner as described above for lung/NALT tissue. Cells were passed through a 70 μm cell strainer and resuspended in T cell media. For draining lymph node analysis, 3 × 10^6^ cells from cLN or 0.8 × 10^6^ cells from ilN were stained. For tissue analysis, 1 × 10^6^ cells from quadriceps or nasal tissue were stained. Cells were washed in FACS buffer (PBS, 1% FBS, 2 mM EDTA, 0.05% sodium azide) and blocked with Fc block for 30 m at 4 °C. Surface antibodies (Table EV1) were diluted in Brilliant Stain Buffer (BD Biosciences), and cells were stained for 1 h. Cells were extensively washed in PBS/2 mM EDTA before staining with LIVE/DEAD Fixable Aqua Dead Cell Stain in PBS/2 mM EDTA for 20 m at 4 °C. Cells were washed with PBS/2 mM EDTA and fixed in IC fixation buffer. FACS was performed on a Novocyte3000 flow cytometer and analyzed using FlowJo software. Remaining cells from tissues were placed in TRIzol reagent (ThermoFisher), and RNA was extracted and isolated utilizing a PureLink RNA kit (ThermoFisher) columns according to the manufacturer’s protocol. cDNA was synthesized from the isolated RNA utilizing the Maxima H Minus First Strand cDNA synthesis kit (ThermoFisher) according to the manufacturer’s protocol. 10 ng of cDNA was subsequently amplified and quantified with PowerUp SYBR Green (ThermoFisher) according to the manufacturer’s fast cycling mode with Prime Time primers (Table EV2) utilizing a QuantStudio 7 (ThermoFisher). Reactions were 50 °C for 2 m, 95 °C for 2 m, followed by cycles of 95 °C for 1 s and 60 °C for 1 m. Results were analyzed using the 2-∆∆ Ct method and normalized to *β-actin* levels.

### Characterization of IFN-I signaling at priming in heterologous immunization

Mice were injected intraperitoneally with 200 μg of IFNαR (MAR1-5A3; Leinco Technologies) blocking or isotype (HKSP; Leinco Technologies) antibody diluted in sterile PBS the day before and day of prime immunization with IM mRNA. Efficacy of treatment was established by saphenous blood collection in EDTA-coated collection tubes and staining for surface IFNαR expression on B cells and T cells with comparison to IFNαR knockout mice on a C57/BL6 background (kind gift from James Moon, University of Michigan). Staining was performed in FACS buffer for 20 m at 4 °C, followed by RBC removal with fixation with RBC lysis/fixation solution (Biolegend) according to the manufacturer’s instructions. Mice were subsequently primed immunized with 1.25 μg of mRNA and boosted twice IN with 15 μg of WT-S adjuvanted with NE/IVT every 4 weeks. The dose of S protein was reduced in order to better observe differences between groups. Mice were characterized 3 weeks post final boost immunization.

### Statistical analysis

Data were analyzed and visualized with Prism V11 (GraphPad). Statistical significance was determined with the Mann–Whitney *U* test, one-way ANOVA, Kruskal–Wallis test, two-way ANOVA or mixed-effects modeling as indicated in figure legends with post-hoc Tukey or Dunn’s multiple comparison test as indicated. Data are represented as means ± SEM as indicated in figure legends. Investigators were not blinded during experiments and outcome assessment. No samples were excluded from the analysis.

## Supplementary information

Table EV1

Table EV2

Appendix

Peer Review File

Dataset EV1

Source data Fig. 1

Source data Fig. 2

Source data Fig. 3

Source data Fig. 4

Source data Fig. 5

Source data Fig. 6

Source data Fig. 7

Expanded View Figures

## Data Availability

Source data available in the following database record: biostudies:S-BSST3402. The source data of this paper are collected in the following database record: biostudies:S-SCDT-10_1038-S44321-026-00539-w.
