# Supplementary material for: Spatiotemporal heterologous vaccination relies on type I interferons at priming to enhance mucosal immunity
Source: EMBO Mol Med. 2026 Oct 12;18(11):4585–617. doi: 10.1038/s44321-026-00539-w (PMC13748547; doi:10.1038/s44321-026-00539-w)
Supplement: Supplementary file 1 [file 44321_2026_539_MOESM13_ESM.pdf]

Expanded View Figures

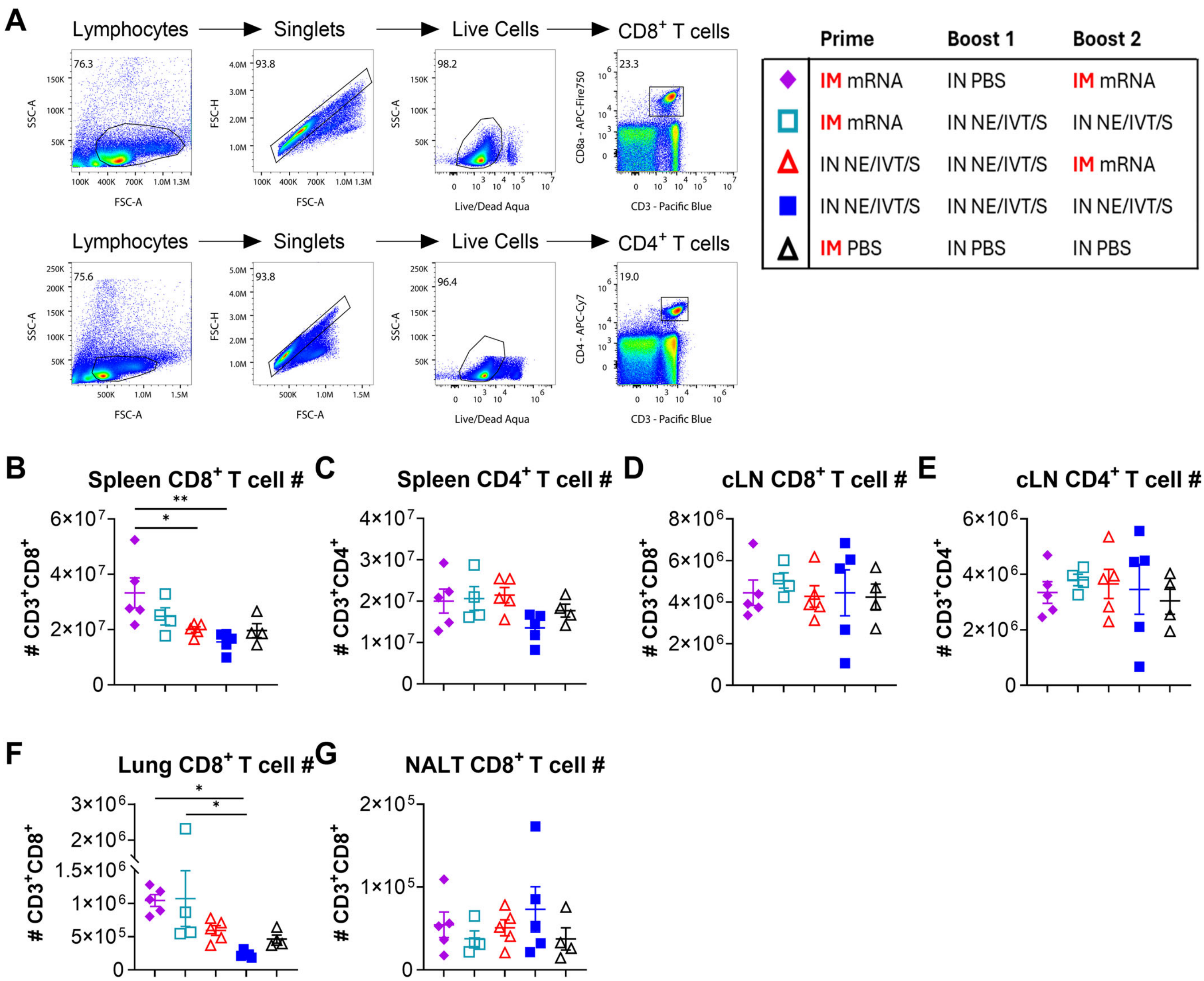

Figure EV1. Homologous IM mRNA-LNP vaccination enhances the frequency of total CD8⁺ T cells within the circulation.

Single cell suspensions from the spleens, cervical lymph nodes, lungs, and NALT of immunized C57BL/6 J mice were isolated 2 weeks post prime/boost/boost immunization. Cells were stained for viability, surface markers, and tetramer. (**A**) Parent gating for analysis. CD8⁺ T cell numbers within the (**B**) spleen, (**D**) cLN, (**F**) lungs, and (**G**) NALT. CD4⁺ T cell numbers within the (**C**) spleen and (**E**) cLN. Graphs representative of mean ± SEM with significance determined with one-way ANOVA with post-hoc Tukey's multiple comparison test. ($n = 4\_5$/immunized groups, $n = 4$/group PBS controls) $^{**}P < 0.01$, $^{***}P < 0.001$, $^{****}P < 0.0001$ shown for select groups (full statistical analysis with exact P values provided in Dataset EV1).

© The Author(s)

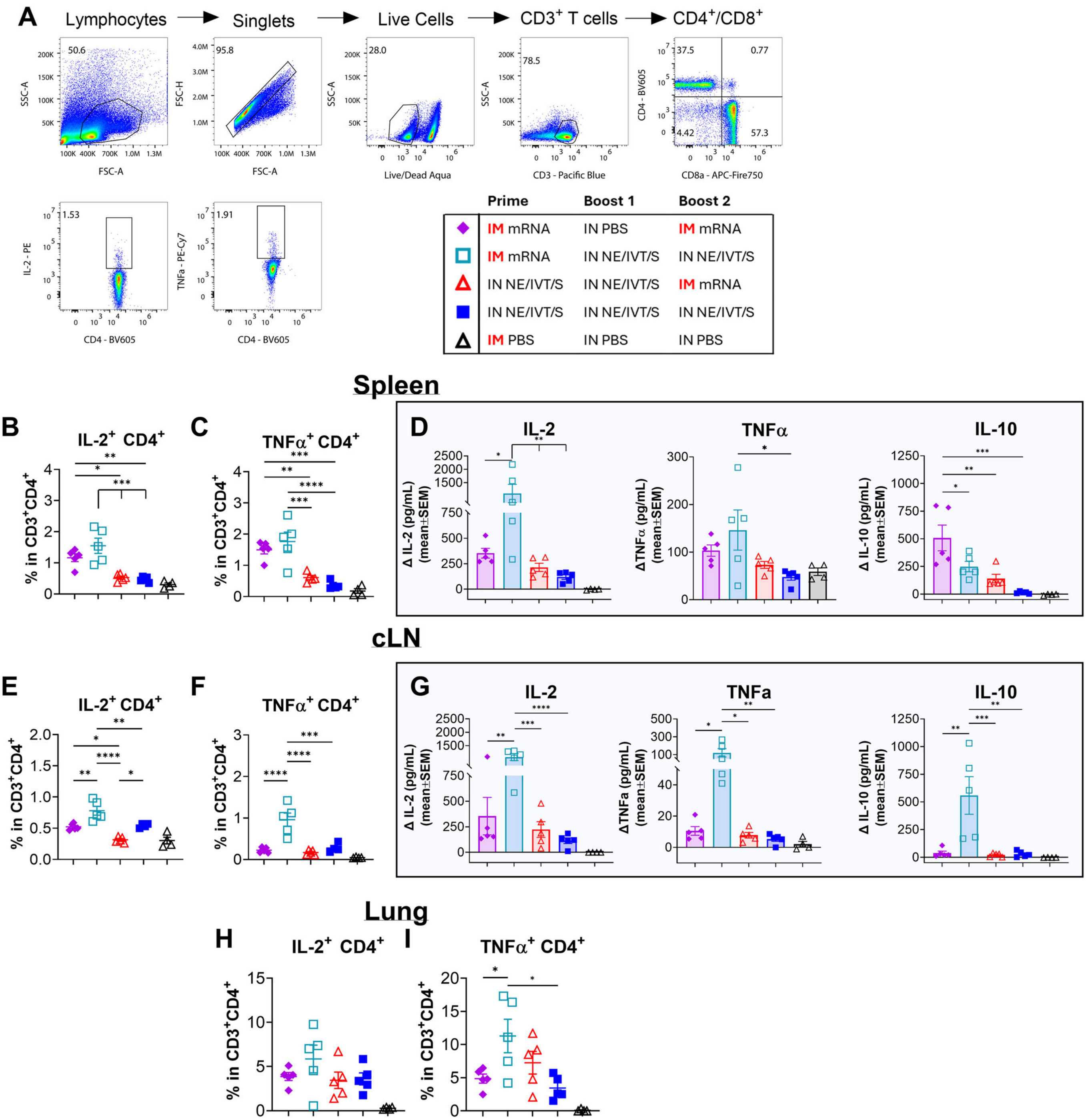

Figure EV2. Heterologous immunization enhances Th1 response in spleen and cLN with high dependence on the sequence of immunization.

Single cell suspensions from the spleens, cLN, and lungs of immunized C57BL/6J mice were isolated 2 weeks post prime/boost/boost immunization and stimulated with 25 μg/mL SARS-CoV2 WT S protein. (**A**) Cells were stained for viability, surface markers, and intracellular cytokines and gated accordingly. Frequency of IL-2 and TNFα expressing CD4+ T cells 24 h after stimulation in (**B**, **C**) spleen, (**E**, **F**) cLN, and (**H**, **I**) lung assessed by flow cytometry. Cytokine production in supernatants from S-stimulated (**D**) spleen and (**G**) cLN cells after 3 days was measured by bead-based Luminex assay and normalized to media controls. ($n = 4$–5/immunized groups, $n = 4$/PBS controls) Graphs representative of mean ± SEM with significance determined with one-way ANOVA with post-hoc Tukey's multiple comparison test. $P < 0.05$, $^{**}P < 0.01$, $^{***}P < 0.001$, $^{****}P < 0.0001$ shown for select groups (full statistical analysis with exact *P* values provided in Dataset EV1).

 © The Author(s)

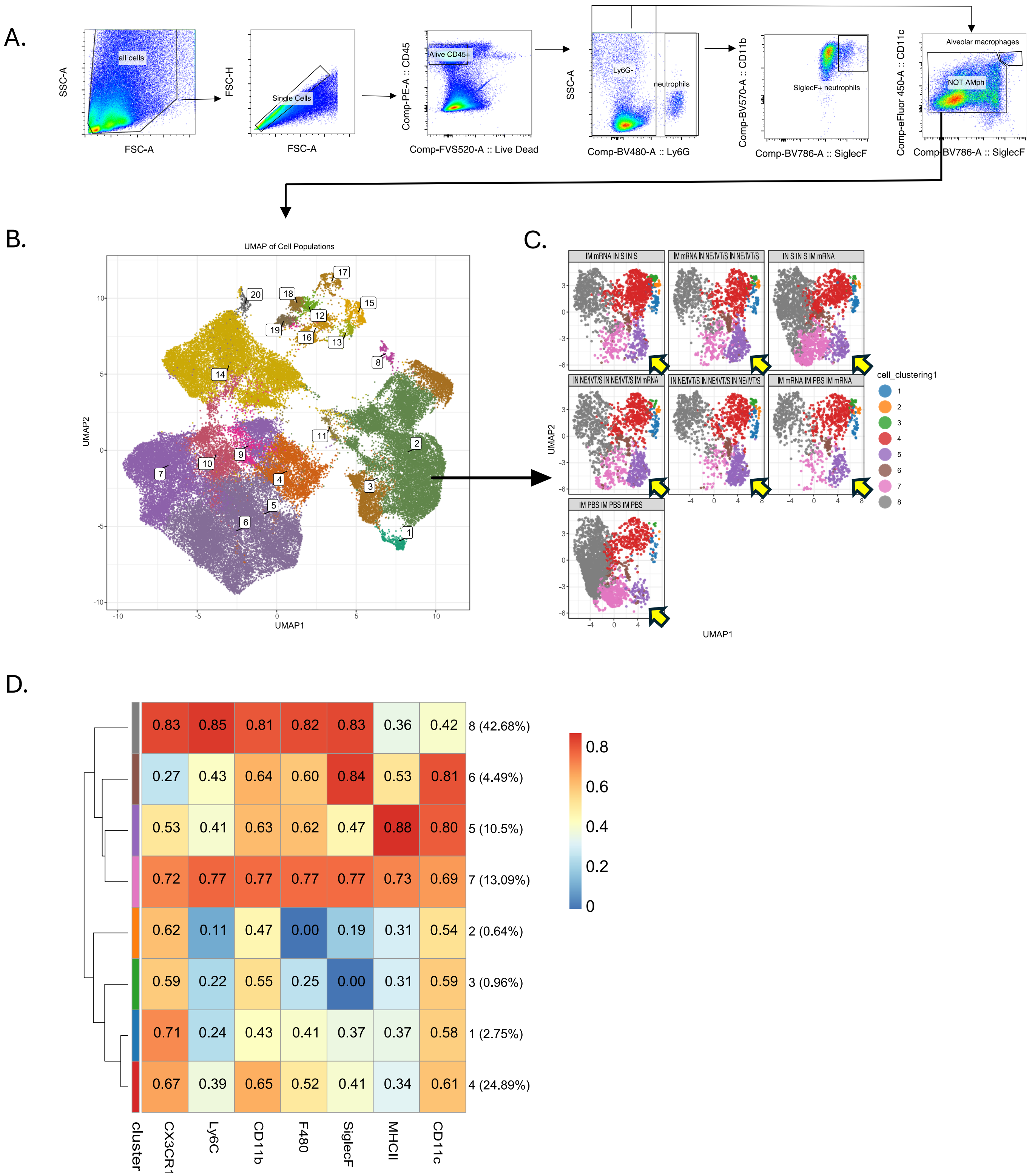
A.
all cells
SSC-A
FSC-A
Single Cells
FSC-H
Alive CD45+
Comp-PE-A :: CD45
Comp-FVS520-A :: Live Dead
Ly6G-
neutrophils
SSC-A
Comp-BV480-A :: Ly6G
SiglecF+ neutrophils
Comp-BV570-A :: CD11b
Comp-BV786-A :: SiglecF
Alveolar macrophages
NOT AMph
Comp-eFluor 450-A :: CD11c
Comp-BV786-A :: SiglecF
B.
UMAP of Cell Populations
UMAP2
UMAP1
C.
IM mRNA IN S IN S
IM mRNA IN NE/NT/S IN NE/NT/S
IN S IN S IM mRNA
IN NE/NT/S IN NE/NT/S IM mRNA
IN NE/NT/S IN NE/NT/S IN NE/NT/S
IM mRNA IM PBS IM mRNA
IM PBS IM PBS IM PBS
cell_clustering1
UMAP1
D.
0.83 0.85 0.81 0.82 0.83 0.36 0.42 8 (42.68%)
0.27 0.43 0.64 0.60 0.84 0.53 0.81 6 (4.49%)
0.53 0.41 0.63 0.62 0.47 0.88 0.80 5 (10.5%)
0.72 0.77 0.77 0.77 0.77 0.73 0.69 7 (13.09%)
0.62 0.11 0.47 0.00 0.19 0.31 0.54 2 (0.64%)
0.59 0.22 0.55 0.25 0.00 0.31 0.59 3 (0.96%)
0.71 0.24 0.43 0.41 0.37 0.37 0.58 1 (2.75%)
0.67 0.39 0.65 0.52 0.41 0.34 0.61 4 (24.89%)
cluster
CX3CR1
Ly6C
CD11b
F480
SiglecF
MHCII
CD11c

© The Author(s)

Figure EV3. Vaccination regimen shapes the myeloid cell infiltration in the lungs of infected mice.

(**A**) Gating strategy to identify total neutrophils (Ly6G$^+$), activated neutrophils (SiglecF$^+$), and alveolar macrophages (CD11c$^+$SiglecF$^+$). (**B**) Lung immune cells (CD45$^+$) that were not alveolar macrophages or neutrophils were further subdivided into 20 clusters by unsupervised analysis using the following markers: CD11b, CD11c, SiglecF, MHCII, F4/80, Ly6c, Arginase, iNOS SIRPa, and CX3CR1 to investigate myeloid cell populations in lungs of infected K18-hACE2 mice. (**C**) Cluster 2 was further clustered into eight subclusters using the following markers: CD11b, CD11c, SiglecF, MHCII, F4/80, Ly6c, and CX3CR1, and revealed cell populations that were enriched depending on the vaccination regimen. Subcluster 5 is enriched for the vaccination groups that received NE/IVT/S (yellow arrows). (**D**) Heatmap representing expression levels for markers used for subclustering cluster 2. Cluster numbers are given on the right, followed by the percentage of cells represented by the specific cluster between parentheses ($n = 5$/group).

 © The Author(s)

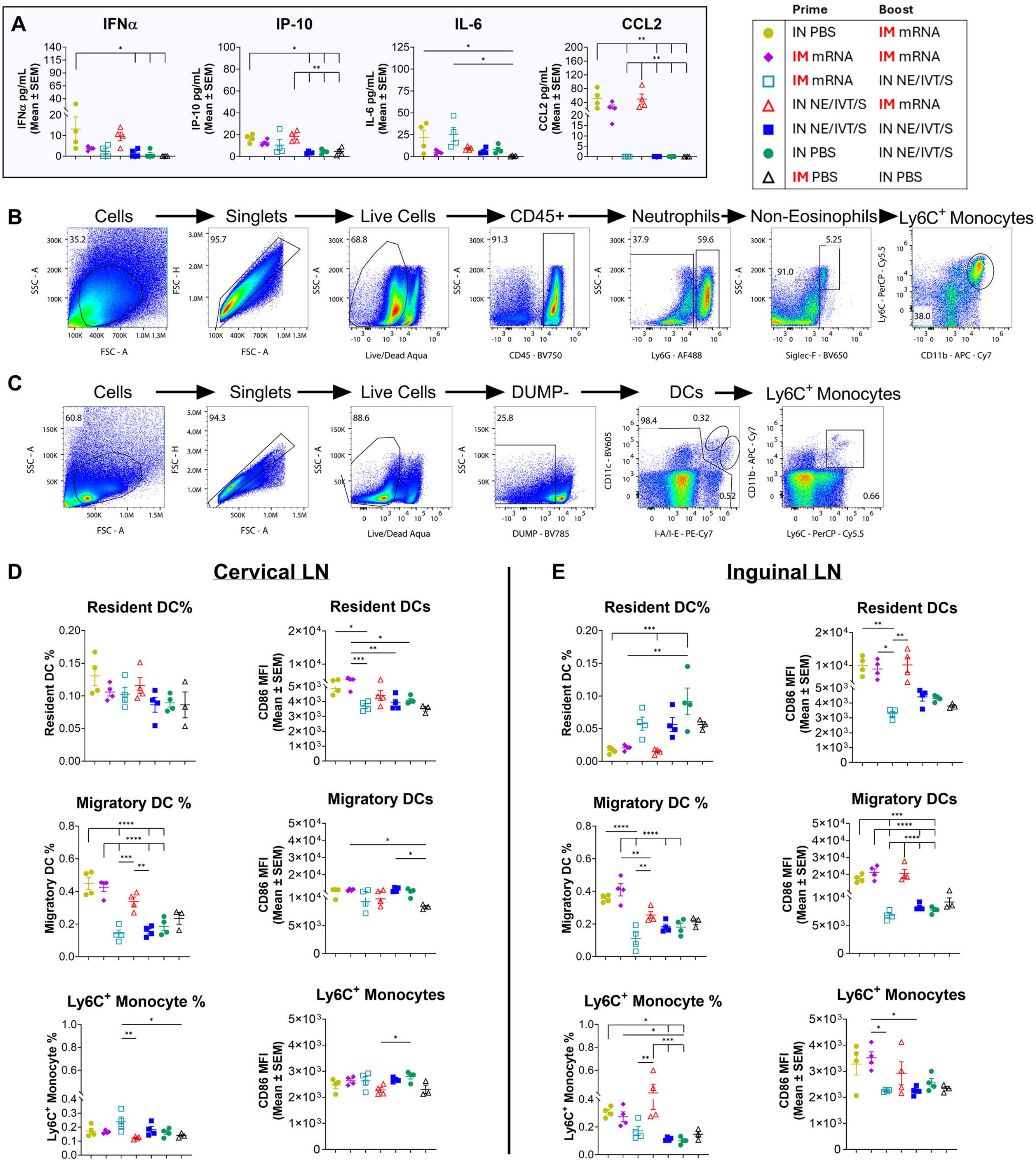
A
IFNα
IP-10
IL-6
CCL2
Prime Boost
IN PBS IM mRNA
IM mRNA IM mRNA
IM mRNA IN NE/IVT/S
IN NE/IVT/S IM mRNA
IN NE/IVT/S IN NE/IVT/S
IN PBS IN NE/IVT/S
IM PBS IN PBS
B
Cells
Singlets
Live Cells
CD45+
Neutrophils
Non-Eosinophils
Ly6C+ Monocytes
C
Cells
Singlets
Live Cells
DUMP-
DCs
Ly6C+ Monocytes
D
Cervical LN
E
Inguinal LN
Resident DC%
Resident DCs
Migratory DC %
Migratory DCs
Ly6C+ Monocyte %
Ly6C+ Monocytes

Figure EV4. IM mRNA immunization induces enhanced inflammatory responses both locally and distally, while IN NE/IVT/S immunization induces only local inflammatory effects.

C57BL/6J mice were immunized in a prime/boost schedule at 4-week intervals. (**A**) Serum levels of IFNα, IP-10, IL-6, and CCL2 24 h post-immunization assessed by bead-based Luminex. ($n = 4$/group) (**B**) Gating pathway for neutrophils ($CD45^+Ly6G^+$) and $Ly6C^+$ monocytes ($CD45^+Ly6G^-Siglec\text{-}F^-Ly6C^+CD11b^+$) in nasal tissue and quadriceps muscle. (**C**) Gating pathway for resident DCs ($CD3/CD19^-MHC\text{-}II^{mid}CD11c^+$), migratory DCs ($CD3/CD19^-MHC\text{-}II^{high}CD11c^+$) and Ly6C+ monocytes ($CD3/CD19^-MHC\text{-}II^{mid}CD11c^-Ly6C^+CD11b^+$) in draining lymph nodes. Resident DCs, migratory DCs, and $Ly6C^+$ monocytes frequencies in live singlet cells and CD86 median fluorescent expression in each subset in cervical lymph nodes (**D**) and inguinal lymph nodes (**E**) assessed by flow cytometry. ($n = 4$/immunized groups, $n = 3$/group PBS controls) Graphs representative of mean ± SEM with significance determined by one-way ANOVA with post-hoc Tukey's multiple comparison test. *$P < 0.05$, **$P < 0.01$, ***$P < 0.001$, ****$P < 0.0001$ shown for select groups (full statistical analysis with exact P values provided in Dataset EV1).

 © The Author(s)

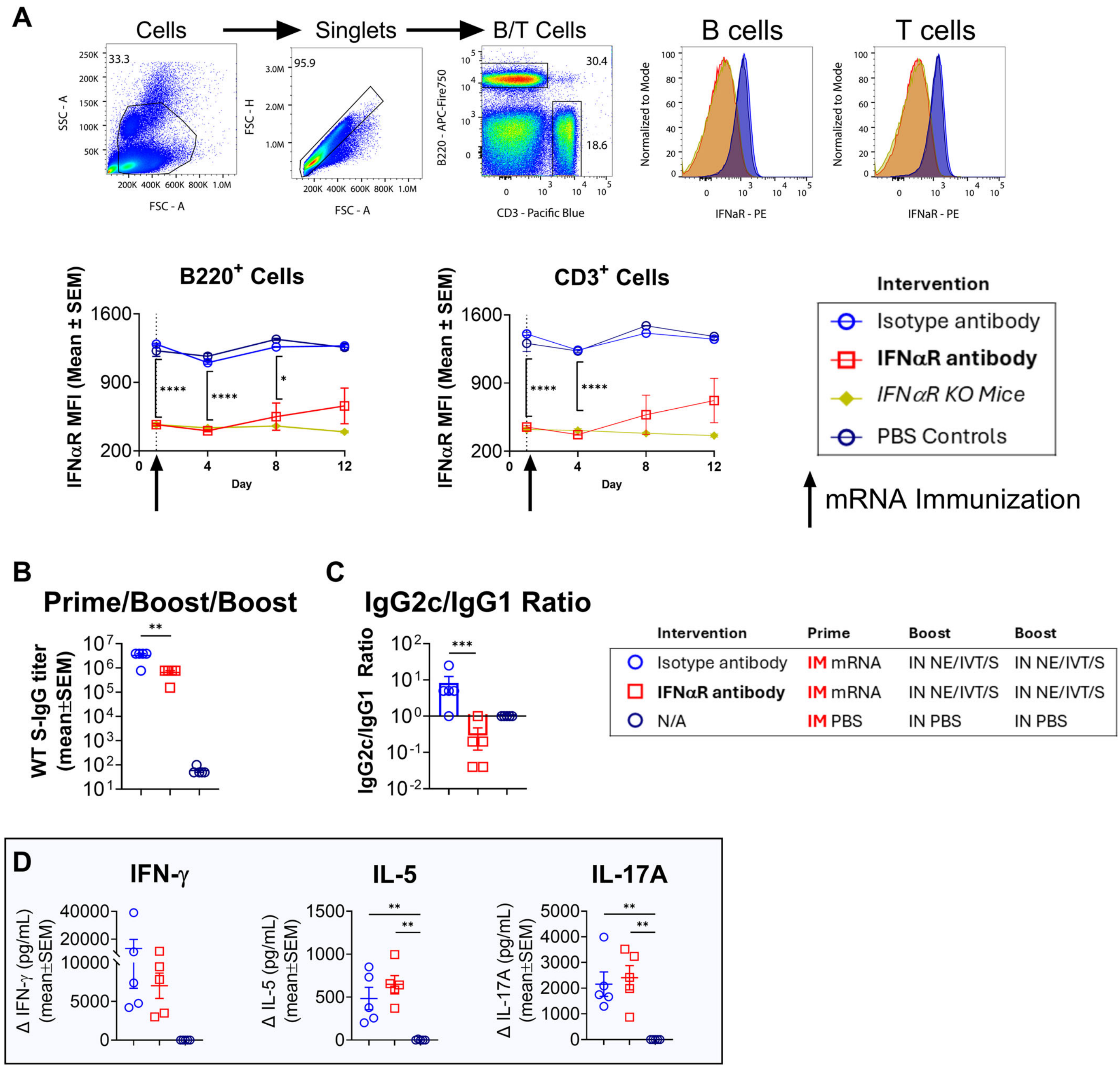

Figure EV5. IFNαR blocking at IM mRNA prime immunization affects antibody class switching but does not affect cytokine response in the spleen.

IFNαR expression was blocked with an antibody at prime immunization, and C57BL/6J mice were subsequently immunized in a prime/boost/boost schedule at 4-week intervals (Scheme in Fig. 6F). (**A**) Flow cytometry gating for determination of IFNαR surface cellular expression and mean fluorescent surface expression on B and T cells over time, assessed by flow cytometry. (n = 4–10/groups at day 1, n = 3-4/groups at days 4–12) (**B**) Serum WT-S IgG levels 6 weeks post prime/boost/boost assessed by ELISA. (**C**) Serum WT-S IgG2c/IgG1 ratios 6 weeks post-prime/boost/boost assessed by ELISA. Cytokine production in supernatants from S-stimulated spleen cells (**D**) after 3 days was measured by bead-based Luminex assay and normalized to media controls. (n = 5/group) Graphs representative of mean ± SEM with titers log10-transformed prior to statistical analysis. Statistical differences assessed by mixed-effects analysis with post-hoc Tukey's multiple comparison test (IFNαR expression) or one-way ANOVA with post-hoc Tukey's multiple comparison test. *P < 0.05, **P < 0.01, ***P < 0.001, ****P < 0.0001 shown for select groups (full statistical analysis with exact P values provided in Dataset EV1).

© The Author(s)
