# Supplementary material for: Spatiotemporal heterologous vaccination relies on type I interferons at priming to enhance mucosal immunity
Source: EMBO Mol Med. 2026 Oct 12;18(11):4585–617. doi: 10.1038/s44321-026-00539-w (PMC13748547; doi:10.1038/s44321-026-00539-w)
Supplement: Supplementary file 2 [file 44321_2026_539_MOESM3_ESM.pdf]

Appendix

Spatiotemporal heterologous vaccination relies on type I interferons at priming to enhance mucosal immunity

Matthew J Wiest[1,2], Gabriel Laghlali[3-5], Julianne d'Acunzo[1], Chinghan Chung[1], Dilara Karadag[1,2], Vivian Yan[3,4,6], Seok-Chan Park[3,4], Moataz Noureddine[3,4,6], Katarzyna W Janczak[1,2], Chun Chen[1,2], Mohammad Farazuddin[1,2], Jessica J O'Konek[1,2], Adolfo García-Sastre[3,4,8-10], James R Baker Jr[1,2], Michael Schotsaert[3,4,7,8,11*], Pamela T Wong[1,2,12, 13*]

Table of Contents

Appendix Figure S1. Route of priming immunization in heterologous immunization drives antibody class switching 3

Appendix Figure S2. Heterologous immunization induces neutralizing antibodies in K18-hACE2 mice challenge study which restricts virus access to the brain 4

Appendix Figure S3. Heterologous immunization with NE/IVT/S boosting induces a more durable cellular response both systemically and in mucosal tissues...5

Appendix Figure S4. Delayed mucosal NE/IVT/S boosting of homologous IM mRNA can establish T cell immunity within the draining lymph nodes, while heterologous prime/boost regimens are more amenable to delayed boosting 6

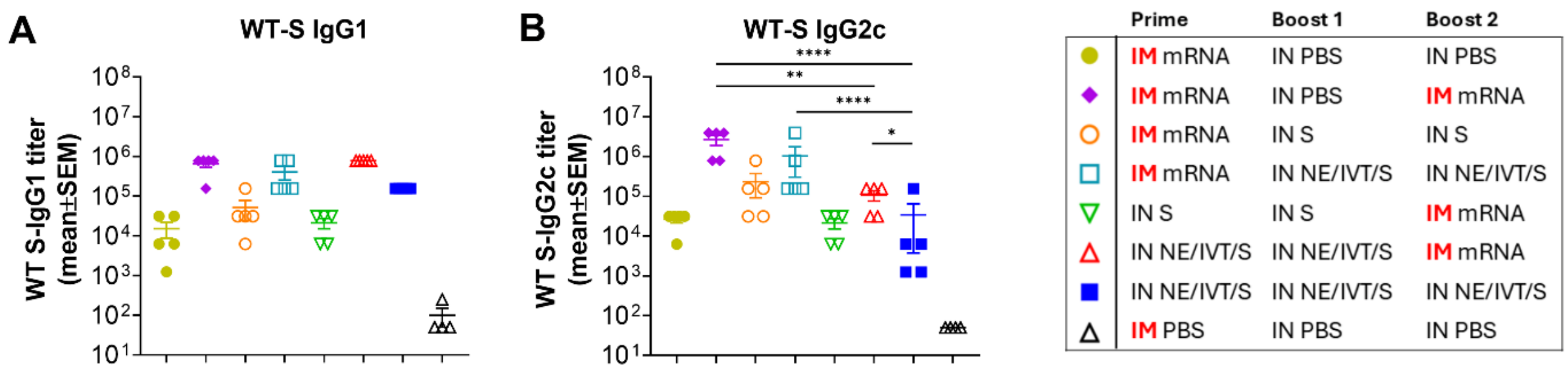

Appendix Figure. S1. Route of priming immunization in heterologous immunization drives antibody class switching. C57BL/6J mice were immunized according to a prime/boost/boost schedule at 3 wks interval (Scheme in **Figure 1A**). Serum levels of WT S-specific (**A**) IgG1 and (**B**) IgG2c 2 wks post prime/boost/boost immunization were determined by ELISA. Graphs representative of mean ± SEM with titers log10-transformed prior to statistical analysis with group differences assessed by one-way ANOVA with post-hoc Tukey's multiple comparison test. (n=5 immunized groups; n=4 PBS controls) $*p<0.05$, $**p<0.01$, $****p<0.0001$ for select groups (Full statistical analysis with exact p values provided in **Dataset EV1**).

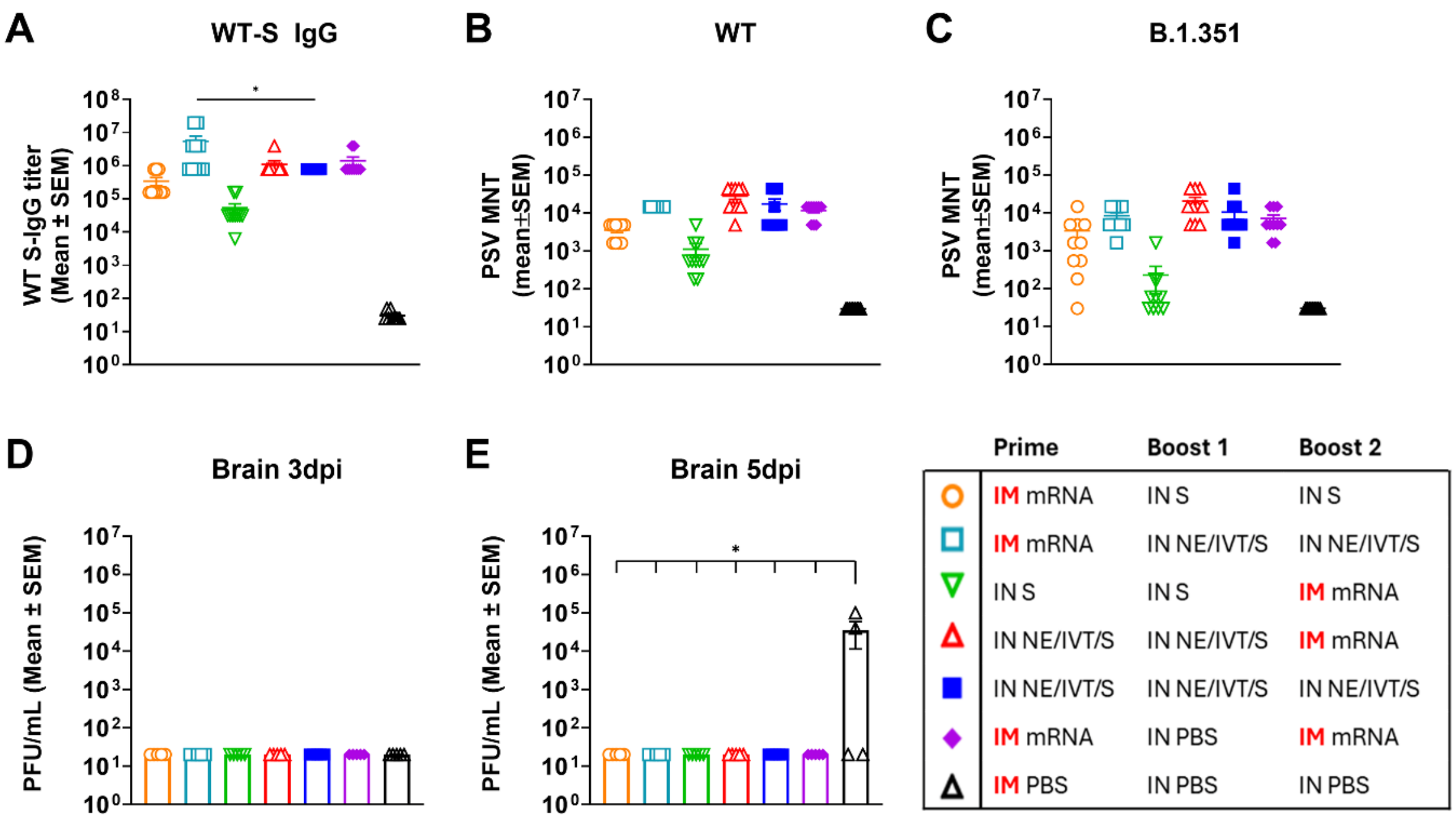

Appendix Figure S2. Heterologous immunization induces neutralizing antibodies in K18-hACE2 mice challenge study which restricts virus access to the brain. K18-hACE2 mice were immunized in a prime/boost/boost schedule at 3 wks interval followed by viral challenge with 10^4PFU of B.1351 SARS-CoV-2, 3 wks post-final boost as shown in **Fig. 4A**. Serum levels of (**A**) WT-S IgG 2 wks post prime/boost/boost immunization were determined by ELISA. Serum neutralization antibody titers to (**B**) WT PSV and (**C**) B.1.351 PSV variants 2 wks post prime/boost/boost immunization as measured by microneutralization assay (n=10 immunized groups; n=9 PBS controls). Virus was quantified in the brain homogenates at (**D**) 3 dpi and (**E**) 5 dpi by plaque assay (n=5 immunized groups; n=4-5 PBS controls). Graphs representative of mean ± SEM with titers log10-transformed prior to statistical analysis with group differences assessed by one-way ANOVA with post-hoc Tukey's multiple comparison test (Serology) or Kruskal-Wallis test with post-hoc Dunn's multiple comparison test (PFU). *$p<0.05$, shown for select groups (Full statistical analysis with exact p values provided in **Dataset EV1**).

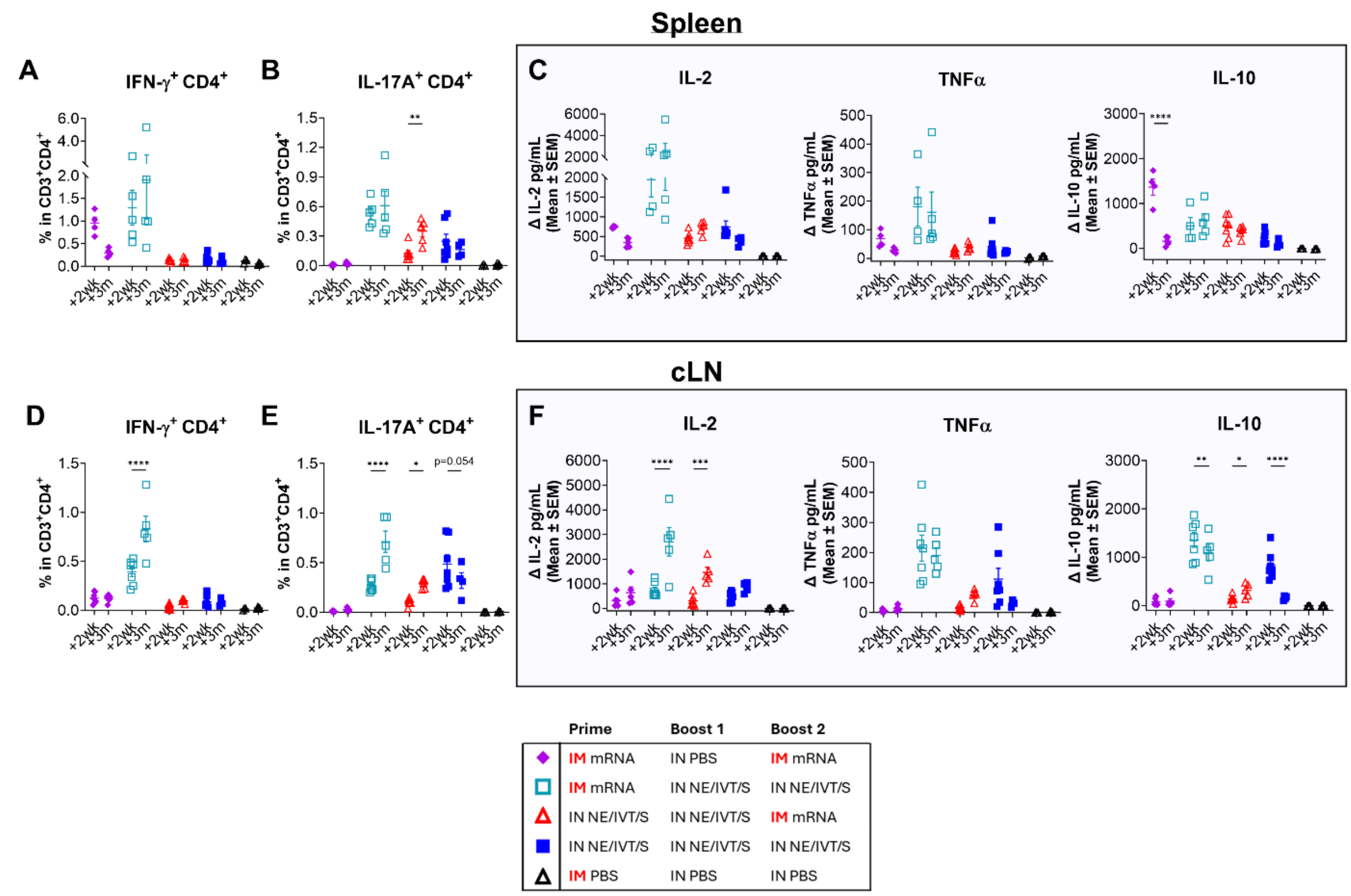

Appendix Figure S3. Heterologous immunization with NE/IVT/S boosting induces a more durable cellular response both systemically and in mucosal tissues. C57BL/6J mice were immunized in a prime/boost/boost schedule at 3 wk intervals with spleen, cervical lymph nodes, and lung harvested at 8 wks (+2 wks post final boost) and at 18 wks (+3m post final boost) (Scheme in **Figure 5A**). Single cell suspensions of spleens and cLNs were stimulated with 25µg/mL SARS-CoV2 WT S protein. (**A-B**) Frequency of cytokine expressing $CD4^+$ T cells within the spleen 24 h after stimulation assessed by flow cytometry. (**C**) Cytokine production in supernatants from S-stimulated spleen cells after 3 d was measured by bead-based Luminex assay and normalized to media controls. (**D-E**) Frequency of cytokine expressing $CD4^+$ T cells within the cLN 24 h after stimulation assessed by flow cytometry. (**F**) Cytokine production in supernatants from S-stimulated cLN cells after 3 d was measured by bead-based Luminex assay and normalized to media controls. Graphs representative of mean ± SEM with significance determined by mixed-effects model with post-hoc Tukey's multiple comparison test. (n=4-7 immunized groups; n=3-4 PBS controls) *$p<0.05$, **$p<0.01$, ***$p<0.001$, ****$p<0.0001$ shown for select groups (Full statistical analysis with exact p values provided in **Dataset EV1**).

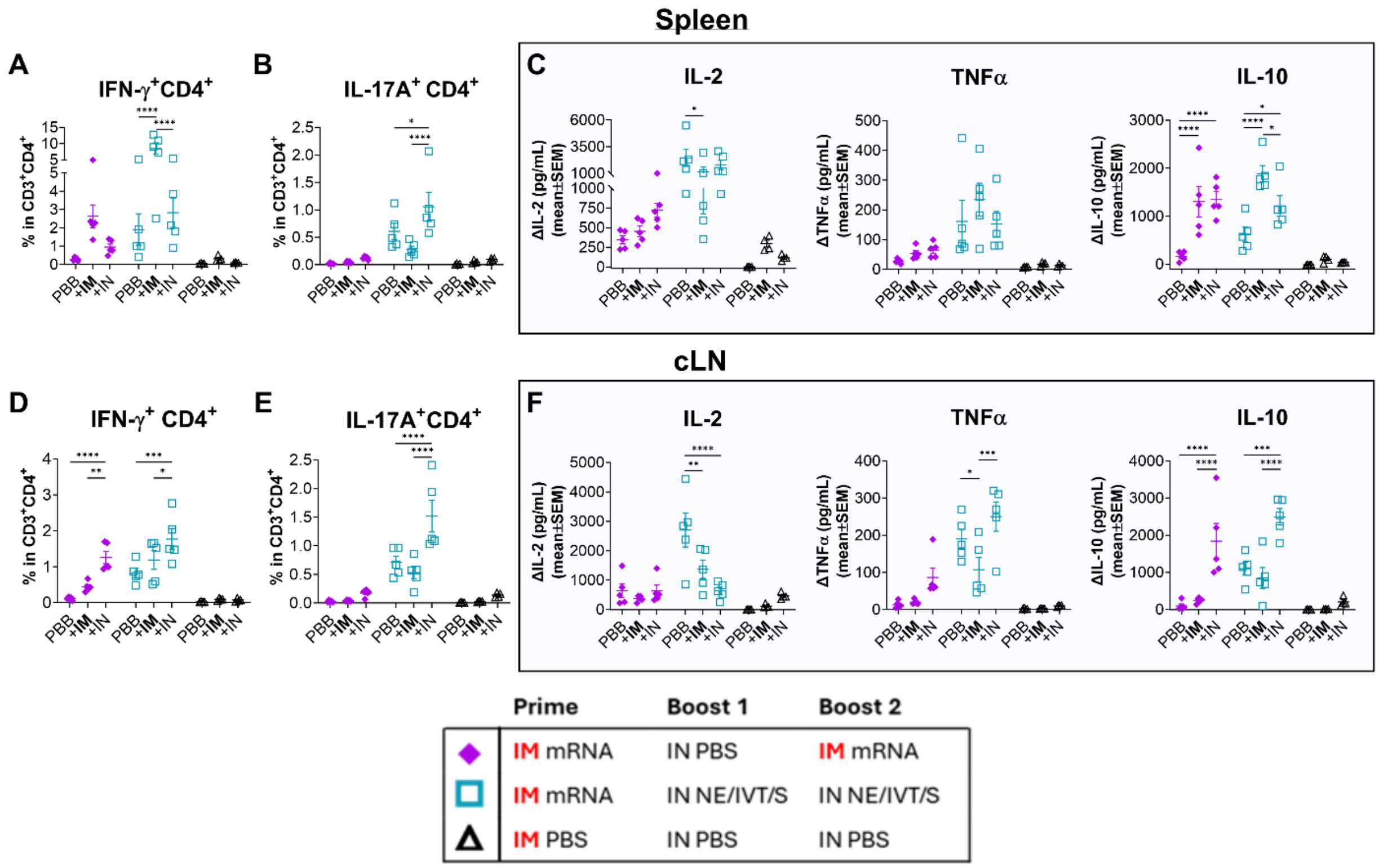

Appendix Figure S4. Delayed mucosal NE/IVT/S boosting of homologous IM mRNA can establish T cell immunity within the draining lymph nodes, while heterologous prime/boost regimens are more amenable to delayed boosting. C57BL/6J mice were immunized with a prime/boost/boost schedule at 3 wk intervals. Immune responses were evaluated 3 mo post-final boost of the initial prime/boost/boost regimen with no further treatment (PBB). Other mice receiving this same initial regimen were given a fourth immunization 3 mo (wk18) after the last immunization of the initial regimen consisting of either IM mRNA (+**IM**) or IN NE/IVT/S (+IN) to evaluate the boost effect at this late time point. (Scheme in **Figure 7A**). Single cell suspensions of spleens and cLNs were stimulated with 25μg/mL SARS-CoV2 WT S protein. Frequency of (**A**) IFN-γ and (**B**) IL-17A expressing CD4^{+} T cells in spleen after 24 h stimulation assessed by flow cytometry. (**C**) Cytokine production in supernatants from S-stimulated spleen cells after 3 d was measured by bead-based Luminex assay and normalized to media controls. Frequency of (**D**) IFN-γ and (**E**) IL-17A expressing CD4^{+} T cells in spleen after 24 h stimulation assessed by flow cytometry. (**F**) Cytokine production in supernatants from S-stimulated spleen cells after 3 d was measured by bead-based Luminex assay and normalized to media controls. Graphs representative of mean ± SEM with significance determined by two-way ANOVA with post-hoc Tukey's multiple comparison test. (n=5 immunized groups; n=4 PBS controls) *p<0.05, **p<0.01, ***p<0.001, ****p<0.0001 shown for select groups (Full statistical analysis with exact p values provided in **Dataset EV1**).
