# Supplementary material for: Spatiotemporal heterologous vaccination relies on type I interferons at priming to enhance mucosal immunity
Source: EMBO Mol Med. 2026 Oct 12;18(11):4585–617. doi: 10.1038/s44321-026-00539-w (PMC13748547; doi:10.1038/s44321-026-00539-w)
Supplement: Supplementary file 3 [file 44321_2026_539_MOESM4_ESM.pdf]

EMBO Molecular Medicine

Spatiotemporal heterologous vaccination relies on type I interferons at priming to enhance mucosal immunity

Matthew Wiest, Gabriel Laghlali, Julianne d'Acunzo, Chinghan Chung, Dilara Karadag, Vivian Yan, Seok-Chan Park, Moataz Noureddine, Katarzyna Janczak, Chun Chen, Mohammad Farazuddin, Jessica O'Konek, Adolfo Garcia-Sastre, James Baker, Jr., Michael Schotsaert, and Pamela Wong

Corresponding author(s): Pamela Wong (ptw@umich.edu) , Michael Schotsaert (michael.schotsaert@mssm.edu)

Review Timeline:

Submission Date:	15th Sep 25
Editorial Decision:	15th Oct 25
Appeal Received:	20th May 26
Editorial Decision:	3rd Jul 26
Revision Received:	2nd Aug 26
Editorial Decision:	16th Sep 26
Revision Received:	21st Sep 26
Accepted:	23rd Sep 26

Editor: Lise Roth

Transaction Report:

(Note: With the exception of the correction of typographical or spelling errors that could be a source of ambiguity, letters and reports are not edited. Depending on transfer agreements, referee reports obtained elsewhere may or may not be included in this compilation. Referee reports are anonymous unless the Referee chooses to sign their reports.)

15th Oct 2025

Dear Dr. Wong,

Thank you for the submission of your manuscript to EMBO Molecular Medicine, and please accept my apologies for the delay in getting back to you as I was waiting for the third referee's report. Unfortunately, we still haven't heard from this referee. Given that the two other referees provide similar recommendations, we prefer to make a decision now in order to avoid further delay in the process. Should referee #1 provide his/her feedback, we would forward it to you.

As you will see from the reports below, the referees acknowledge the potential interest of the study, however they also raise major concerns that question the robustness of the results and the conclusions drawn.

Based on the referees' feedback and given that at EMBO Press we encourage a single round of revisions in a limited time frame, I am afraid I see little choice but to return the manuscript to you at this point with the decision that we cannot offer to publish it.

I am very sorry to disappoint you in this occasion, and hope that the referees' comments are helpful in your continued work in this area.

Sincerely,

Lise Roth

Lise Roth
Senior Editor
EMBO Molecular Medicine

***** Reviewer's comments *****

Referee #2 (Comments on Novelty/Model System for Author):

Unstimulated controls were not shown for the data where spike antigen were used to stimulate the cells.

Referee #2 (Remarks for Author):

In this manuscript, the authors evaluated how different immunization protocols and sequence (homologous and heterologous vaccinations given in different sequences), immunization routes (intramuscular VS intranasal), vaccine preparations (mRNA VS subunit) and adjuvants (unadjuvanted VS nanoemulsion + RIG-I agonist [NE/IVT]) influence the vaccine-induced mucosal (lung and nasal associated lymphoid tissue) and systemic (peripheral blood and spleen; cervical lymph nodes) humoral and T cell immunity in mice vaccinated against SARS-CoV-2 Spike antigen.
By measuring the quantity of vaccine-induced Spike-specific antibodies, Spike-specific T cells and their cytokine secretion profile after activation, the authors observed that while NE/IVT adjuvant can enhance vaccine-induced immunity, the responses were highly influenced by the chronological sequence of vaccine delivery. Importantly, the authors claimed that the prime immunization route "dominantly influenced Th bias and localization" (line 48). This led to the authors to conclude that prime immunization can significantly dictate the responses induced by subsequent antigen exposures with implications in the development of vaccination strategies (line 51-54).
In general, the topic investigated in the manuscript is a timely addition to the field, especially with the growing interest in the study of mucosal immunity during natural infection and vaccination, and the development of mucosal vaccines to effectively induce mucosal immunity against respiratory pathogens. The analysis performed was extensive with many differently vaccinated mice groups leading to multiple comparisons. Unfortunately, there seems to be a lack of distillation of all the points raised, making the entire manuscript seem overly descriptive and hard to read. In addition, their primary conclusion with regards to the dominance of the prime immunization route does not seem to be supported by their data.
Detailed comments are below:
1. In line 47-48 and line 51-54, the authors claimed that the prime immunization route dominantly influenced Th bias and localization, leading to the conclusion that it can dictate responses induced by subsequent antigen exposures. However, the data in multiple figures do not support the conclusion. In figure 2, looking at the Tet+ CD8+ T cells detected in the spleen (Fig 2B) and cervical lymph nodes (Fig 2D; cLN), priming with IM mRNA (●) or with IN NE/IVT/S (●) in the heterologous vaccination groups resulted in the same quantity of Tet+ CD8+ T cells and does not suggest that the prime immunization was affecting

localization. In the lungs (Fig 2G) and NALT (Fig 2H), while priming with IM mRNA (●) or with IN NE/IVT/S (●) in the heterologous vaccination groups resulted in a clear difference in the amount of Tet+ CD8 T cells in the anatomical locations, this cannot be explained by a "dominant influence" of prime immunization as both homologous IM mRNA (●) and IN NE/IVT/S (●) vaccinations did not result in the localization of large quantities of Tet+ CD8+ T cells in both compartments. Instead, data from figure 2G and 2H showed that a strong systemic induction of SARS-CoV-2 cellular immunity by IM mRNA prime followed by intranasal vaccination (●/●) resulted in the dramatic accumulation of mucosal Tet+ CD8+ T cells, but not when the sequence was reversed (●), showing that the boost immunization was dominantly influencing localization instead.
Since the multimer data was obtained from the analysis of a single CD8 or CD4 T cell epitope, it does not fully detect all the vaccine-induced T cell responses and hence additional intracellular cytokine staining and cytokine secretion experiments using the Spike antigen was performed. It is clear from the homologous IN NE/IVT/S vaccination group (●) that the adjuvanted intranasal vaccine is a potent inducer of IL-17 producing cells in the compartments tested (Fig 4F and 4M) while homologous IM mRNA (●) vaccination resulted in only a small quantity of secreted IL-17. Despite being primed with IN NE/IVT/S (●), IL-17 production in the spleen and cLN was lower than the IM mRNA primed heterologous vaccination group (●). Conversely, IL-5 secretion is a cytokine produced mainly by IM mRNA vaccination (●; Fig 4D and 4K). In this case, the heterologous IN NE/IVT/S primed (●) group secreted higher amounts of IL-5 than the heterologous IM mRNA primed vaccination group (●). These results again indicate that the boost immunization was dominantly influencing localization and Th bias, contrary to the author's conclusion.
Even if you consider the intracellular cytokine staining data after Spike antigen stimulation, you can clearly see no difference in IL-17+ CD4+ T cells in the lungs of individuals who received heterologous IM mRNA primed (●) or heterologous IN NE/IVT/S (●) primed vaccinations (Fig. 3N).
Taken together, the final conclusion reached by the authors were not supported by the data shown and hence I cannot recommend the acceptance of this manuscript for publication.

Referee #3 (Comments on Novelty/Model System for Author):

The statistical analysis of the data presented in Figure 1 is not correct.
The novelty is limited as previous studies have shown the benefit of parenteral prime and mucosal booster in the context of SARS-CoV-2 and other respiratory viral infections. However, the authors have provided an extensive and detailed analysis of the immune response.
The medical impact is modest as it seems unlikely that vaccine companies will provide two different types of vaccines for priming and boosting.

Referee #3 (Remarks for Author):

The authors report on experiments investigating the effect of the sequence of immunization intramuscularly with an mRNA vaccine and intranasal with a nanoemulsion adjuvanted protein vaccine on the magnitude, quality and duration of the immune response. The overall conclusion is that intramuscular priming followed by intranasal booster generates the most effective and durable immune response.
The work aims to address a limitation of current vaccines against COVID-19 and other respiratory viruses, namely the lack of induction of an effective mucosal immune response. In addition, mRNA vaccines suffer from short duration of immunity and a boost with a protein subunit vaccine can potentially overcome this shortcoming. The idea that heterologous prime-boost (reviewed by S. Lu, Curr Opinion Immunol, 2009) and parental/mucosal administration strategies (e.g., Keren et al., Inf Immunity 1988) can enhance the immune response and provide better protection against mucosal pathogens is not new. This has also been shown in numerous studies for vaccines targeting SARS-CoV-2 only some of which are cited in the manuscript. It has also been shown that the sequence of parental/mucosal prime/booster affects the immune response (McCluskie et al., FEMS Immunology and Medical Microbiology, 2002). Thus, the novelty of the work lies in the combination of an mRNA vaccine with a mucosal nanoemulsion vaccine and the extensive and detailed analysis of the immune response. The authors are encouraged to perform a more thorough literature review and put their data in the context of published studies. The data support the overall conclusion that the sequence of priming and boosting has a significant effect on the immune response.
Other comments:
1. Figure 1 B - D and F - I. The legend states that antibody titers were compared by Mann-Whitney U-test. This is not appropriate for analysis of experiments with multiple groups. Instead, the authors should analyze log-transformed antibody titers by one-way ANOVA followed by an appropriate multiple comparison test. The Methods state that "titers were determined using a cutoff defined as the sum of the average absorbance at the lowest dilution of naïve serum and two times the standard deviation (line 1041-1043). It is not clear how this results in antibody titers of 1 for the PBS only and IN S only groups (same comment for Figure 6 B - D).
2. Figure 1 J, K. The graphs report OD readings instead of antibody titers as suggested in the Methods (lines 1035 - 1043). The dilution of the BAL fluid should be given.
3. Figure 1 (line 256). 3 - 20 mice/group. This is not reflected in the numbers depicted in the graphs.
4. All figure legends. Please add a sentence of what the bars show (mean/geometric mean, SD or SEM).
5. Line 307-309. "no notable differences in the overall frequencies of total CD8+ or CD4+ T cells in the cLN were observed between groups...". Presumably the lymph nodes of immunized mice were enlarged and contained more lymphocytes than

control lymph nodes. A more meaningful indication would be the total number (absolute instead of percentage) of antigen-specific CD4 and CD8 T cells.
6. Figure 3D and 4F. There is an increase of IL-17 secretion in certain groups of mice that was not seen by intracellular cytokine staining. This suggests that cells other than CD4 T cells contribute to the IL-17 secretion. This should be discussed.
7. Line 910. "Delays in vaccination is a significant problem with mRNA vaccines." The genesis of this statement is not clear.
8. Lines 960-961. The correct names of the mouse strains are C57BL/6J and B6.Cg-Tg(K18-ACE2)2Prlmn/J (which can be abbreviated to K18-hACE2 in other parts of the manuscript) and the source is presumably The Jackson Laboratory.

As a service to authors, EMBO provides authors with the possibility to transfer a manuscript that one journal cannot offer to publish to another EMBO publication. The full manuscript and if applicable, reviewers reports are automatically sent to the receiving journal to allow for fast handling and a prompt decision on your manuscript. For more details of this service, and to transfer your manuscript to another EMBO title please click on Link Not Available

Please do not share this URL as it will give anyone who clicks it access to your account.

Reviewer response for EMM-2025-22617:

We thank the reviewers and the editors for taking the time to carefully consider and review our manuscript and truly appreciate the helpful critical comments as well as the encouraging comments regarding the strengths and importance of our work. After reading the comments and re-examining our manuscript closely, we agree that the message was not most effectively conveyed and that the manuscript was too lengthy, making it difficult to follow. We believe that this likely contributed in part to some confusion in the interpretation of our overall message as reflected by some of the comments which indicate potential miscommunication of some of the data. Furthermore, we fully agreed with the reviewers that framing the conclusions as immunization outcomes are purely dominated by the prime immunization, in our efforts to streamline the conclusions, does not accurately capture the data. Rather, we had wanted to convey the point that immunization on an immune-experienced baseline is not the same as immunization on a naïve baseline. To further support this conclusion, we have since performed several additional experiments which identified important distinctions between the IM mRNA vs. the IN NE/IVT/S vaccines, as well as demonstrated the necessity of the IFN-I burst induced by IM mRNA priming to the promotion of enhanced mucosal responses to later subsequent intranasal vaccinations.

We have made significant effort since the previous submission to improve this manuscript accordingly by doing the following: 1. performed major overhaul revisions of the manuscript and figures with the goal of streamlining the data as well as the discussion of the data, 2. removed the prime-dominance discussions and instead more accurately discuss the data in terms of this revised overall conclusion mentioned above, 3. performed several additional studies including tissue-level and systemic-level profiling of the differences in responses to IN or IM immunizations with mRNA or NE/IVT/S on naïve vs. immune-experienced baselines (with experience imparted through different types of vaccinations), as well as mechanistic studies to identify why IM mRNA as a prime alters responses to subsequent mucosal vaccination most optimally, 3. performed additional studies to greatly expand our profiling of differences in the lung immune environment post-challenge between vaccination groups using unsupervised clustering analysis. This now included data revealed significant differences in myeloid cell influx as well as their activation states depending on the vaccination type received, even though these groups showed the same degree of protection, and finally, 4. addressed the reviewer concerns as detailed in the point-by-point responses below. We believe that all these changes have significantly improved the quality of the manuscript and hope that it is now suitable for publication.

As there have been significant revisions throughout the text, we tried to point these out through comments rather than completely highlighting the entire text for all the changes made for the sake of clarity.

***** Reviewer's comments *****

Referee #2 (Comments on Novelty/Model System for Author):

R2: Unstimulated controls were not shown for the data where spike antigen were used to stimulate the cells.
Response: We thank the reviewer for pointing this out. Initially we did not show unstimulated media controls for Luminex multiplex assays, as the levels of cytokine production were consistently at undetectable/insignificant levels for all of the cytokine measurement (for example, in the Luminex for the now Fig. 3, IFNg levels in unstimulated controls were <10pg/mL in the spleen and cLN). Instead, we

show the subtracted differences, which we have re-emphasized in each figure legend. We have also added a statement regarding the unstimulated controls on line 320.

Referee #2 (Remarks for Author):

R2: In this manuscript, the authors evaluated how different immunization protocols and sequence (homologous and heterologous vaccinations given in different sequences), immunization routes (intramuscular VS intranasal), vaccine preparations (mRNA VS subunit) and adjuvants (unadjuvanted VS nanoemulsion + RIG-I agonist [NE/IVT]) influence the vaccine-induced mucosal (lung and nasal associated lymphoid tissue) and systemic (peripheral blood and spleen; cervical lymph nodes) humoral and T cell immunity in mice vaccinated against SARS-CoV-2 Spike antigen.
By measuring the quantity of vaccine-induced Spike-specific antibodies, Spike-specific T cells and their cytokine secretion profile after activation, the authors observed that while NE/IVT adjuvant can enhance vaccine-induced immunity, the responses were highly influenced by the chronological sequence of vaccine delivery. Importantly, the authors claimed that the prime immunization route "dominantly influenced Th bias and localization" (line 48). This led to the authors to conclude that prime immunization can significantly dictate the responses induced by subsequent antigen exposures with implications in the development of vaccination strategies (line 51-54).
In general, the topic investigated in the manuscript is a timely addition to the field, especially with the growing interest in the study of mucosal immunity during natural infection and vaccination, and the development of mucosal vaccines to effectively induce mucosal immunity against respiratory pathogens. The analysis performed was extensive with many differently vaccinated mice groups leading to multiple comparisons. Unfortunately, there seems to be a lack of distillation of all the points raised, making the entire manuscript seem overly descriptive and hard to read. In addition, their primary conclusion with regards to the dominance of the prime immunization route does not seem to be supported by their data.

Response: We thank the reviewer for these constructive comments and appreciate the positive feedback regarding the importance of this research as well as their appreciation of the extensiveness of the analyses performed in our work. We have taken the criticisms pointed out by the reviewer 2 seriously, including the need for a clearer distillation of all the data sets and their overall implications. To address this, since the previous submission, we have performed major overhaul revisions of both the text and the data figures to streamline the data as well as the discussion of the results. We placed major effort into ensuring clearer distillation of the conclusions from each data set and towards improving the logical flow of the manuscript. We also found it to be too lengthy, and reduced unnecessary discussion and data such that the key points are better highlighted. Most importantly, we fully agree that the primary conclusion in the previous version of the manuscript with regards to the dominance of the prime immunization route on overall vaccine responses is not an ideal way to frame the message of the data. In the previous version we sought to simplify the messaging and thus, tried to emphasize the role of the prime immunization. While the importance of the priming immunization remains valid, the results and conclusions are better described as immunization on an immune-experienced baseline is not the same as immunization on a naïve baseline. We have now integrated and emphasized this theme throughout the results. Furthermore, we have since performed extensive additional experiments to characterize differences in the impact of IM mRNA vs. IN NE/IVT/S priming on subsequent vaccination responses (new Figure 6 and new Figure S7). We now show data which highlight the very different nature of the IM mRNA vaccine (systemic+distal

innate effects) vs. the IN NE/IVT/S subunit vaccine (primarily local mucosal innate effects), and demonstrate how the distinct innate activation events triggered by these vaccines at the different tissue locales in which they are delivered, lead to alterations in the innate immune environment in the mucosa as well as in the muscle in terms ISG expression, and the types of cellular infiltrates recruited. Lastly, we performed additional immunization experiments in the context of IFNAR blocking, which identified the critical role of IFN-Is induced by IM mRNA during priming in promoting the observed enhanced mucosal responses to later subsequent mucosal vaccinations. We believe that this new data greatly strengthens our conclusions, providing more mechanistic insights into the divergent immune outcomes observed when the chronological sequence of the heterologous IM mRNA and IN NE/IVT/S vaccines are altered. (IM mRNA→IN NE/IVT/S vs. IN NE/IVT/S→IM mRNA). Additionally, we have greatly expanded our profiling of the lung immune environment in response to viral challenge. Unsupervised clustering analysis of the lungs post-challenge revealed significant differences in the immune cell infiltrates as well as their activation states, particularly within the myeloid cell compartment between vaccination groups. This data is now added in new Figure 4S-U, as well as in new Figure S5.

Detailed comments are below:

We thank the reviewer for carefully considering our data and for these detailed comments below. As these comments by R2 are actually one interrelated comment regarding our former conclusions, we believe that we have now fully addressed these concerns by more accurately framing our conclusions from the data and by the addition of our new data as described above. Instead of "prime dominance", as mentioned above, we fully agree with R2 in that this is oversimplified and not a precise way to convey our interpretation of the data. Instead, the conclusion we were trying to convey is that immunization is influenced by host immune experience, and by how that experience was acquired (by which vaccine and through which immunization route). We believe that our revisions to the data and results now better represent this message more clearly.

We have broken down this single comment by the reviewer into subsections to better address them.

R2: 1. In line 47-48 and line 51-54, the authors claimed that the prime immunization route dominantly influenced Th bias and localization, leading to the conclusion that it can dictate responses induced by subsequent antigen exposures. However, the data in multiple figures do not support the conclusion. In figure 2, looking at the Tet+ CD8+ T cells detected in the spleen (Fig 2B) and cervical lymph nodes (Fig 2D; cLN), priming with IM mRNA (●) or with IN NE/IVT/S (●) in the heterologous vaccination groups resulted in the same quantity of Tet+ CD8+ T cells and does not suggest that the prime immunization was affecting localization. In the lungs (Fig 2G) and NALT (Fig 2H), while priming with IM mRNA (●) or with IN NE/IVT/S (●) in the heterologous vaccination groups resulted in a clear difference in the amount of Tet+ CD8 T cells in the anatomical locations, this cannot be explained by a "dominant influence" of prime immunization as both homologous IM mRNA (●) and IN NE/IVT/S (●) vaccinations did not result in the localization of large quantities of Tet+ CD8+ T cells in both compartments. Instead, data from figure 2G and 2H showed that a strong systemic induction of SARS-CoV-2 cellular immunity by IM mRNA prime followed by intranasal vaccination (●/●) resulted in the dramatic accumulation of mucosal Tet+ CD8+ T cells, but not when the sequence was reversed (●), showing that the boost immunization was dominantly influencing localization instead.

Response: We have now removed the statements indicated in these lines as well as more precisely adjusted our messaging throughout the manuscript to remove references to our former conclusions that prime immunization route dominates the overall vaccination outcomes. Instead, we show that host pre-existing immunity highly influences and shapes the immune responses to later subsequent vaccinations. Furthermore, we have performed more experiments and provide a significant amount of additional data strengthening this conclusion, demonstrating that the type of pre-existing immunity has a major impact on these later responses (Fig. 6, S7).

Please note that to simplify the data and to allow space for the new data sets added, we have now focused on the responses in only the IM mRNA and IN NE/IVT/S homologous and heterologous groups in our new version of Figure 2, as these are the most pertinent groups, as well as reformatted some of the figures for clearer visualization. The data itself in Fig. 2 remains the same.

With regards to the comments on the Tet^{+}CD8 T cell response profiling, we entirely agree with the reviewer's interpretation of this data. We believe we are, in fact, in full alignment and have tried to now better convey our messaging. The enhanced mucosal response is not due to prime dominance nor boost dominance, but rather, a synergistic result of *both* priming and booster vaccines. We also are in full alignment with the reviewer's statement regarding Figs. 2G and 2H that a strong systemic priming event (with the IM mRNA) is clearly needed in order to achieve such accumulation of Tet^{+}CD8 T cells in the mucosal compartment (lungs, NALT, cLN (some)) upon boosting with IN NE/IVT/S. In other words, the synergistically enhanced Tet^{+}CD8 T cell response in the lung/NALT required IM mRNA priming as well as the IN NE/IVT/S boost, as IN NE/IVT/S homologous immunization alone induced only low levels. Furthermore, the reverse sequence of IN NE/IVT/S priming with IM mRNA boosting did not induce these enhanced responses. Therefore, we would argue that the identities and routes of administration of both priming and boosting vaccines and the specific chronological order in which they are administered shape localization. In this case, IM mRNA primes an "immune baseline" which fosters greater responsiveness especially in the mucosa to IN NE/IVT/S boosting and altered innate responses. For example, we now show IM mRNA priming led to differences in immune cell recruitment (ex. $Ly6C^{hi}$ monocytes) to the nasal tissue upon IN NE/IVT boost compared to IN NE/IVT homologous vaccination (new Fig. 6D).

We have also performed more mechanistic studies shown in new Fig. 6F-K demonstrating that local and systemic IFN-I responses during priming is critical for promoting these enhanced mucosal responses to later IN NE/IVT/S vaccination. IM mRNA priming triggers IFN-I responses both within the muscle, systemically, and modestly in the mucosa. By using a blocking Ab to prevent IFN-I signaling during priming, we demonstrate this abolishes the enhanced mucosal responses seen with the IM mRNA→IN NE/IVT/S sequence. On the other hand, IN NE/IVT/S induces robust IFN-I responses primarily only locally within the mucosa, which does not promote enhanced mucosal T cell responses upon later systemic IM mRNA vaccination.

With regards to the spleen (Figs. 2B, C) and cLN (Figs. 2D, E), we also are in agreement with R2 in that the IM mRNA vaccine is a strong activator of systemic CD8 T cell responses, while the IN NE/IVT subunit vaccine is generally better at inducing CD4 T cell responses especially within the mucosal draining lymph nodes. Homologous IM mRNA induces the most optimal Tet^{+}CD8 T_{EM}s in the spleen. Indeed, as the reviewer noted, while the heterologous group with IM mRNA priming (IM mRNA→IN NE/IVT/S) does show trends towards higher Tet^{+}CD8 and CD4 T_{EM}s in the spleen (Figs. 2B, C) and Tet^{+}CD8 T_{EM}s in the cLN than the reverse sequence, the clearest distinction demonstrating the impact of

sequence is in the cLN Tet⁺CD4 T_{EM}s which are significantly enhanced only in the IM mRNA priming (IM mRNA→IN NE/IVT/S) group but not the reverse (Fig 2E). These results are consistent with the fact that the sequence of immunization has the most profound effect on the mucosal response as observed for the lungs and NALT (Figs. 2G, H). We have now made every effort to better emphasize these themes within the text and in the results for Fig. 2 and in the discussion, as well as throughout the other data in the manuscript to improve clarity of the message.

R2: Since the multimer data was obtained from the analysis of a single CD8 or CD4 T cell epitope, it does not fully detect all the vaccine-induced T cell responses and hence additional intracellular cytokine staining and cytokine secretion experiments using the Spike antigen was performed. It is clear from the homologous IN NE/IVT/S vaccination group (●) that the adjuvanted intranasal vaccine is a potent inducer of IL-17 producing cells in the compartments tested (Fig 4F and 4M) while homologous IM mRNA (●) vaccination resulted in only a small quantity of secreted IL-17. Despite being primed with IN NE/IVT/S (●), IL-17 production in the spleen and cLN was lower than the IM mRNA primed heterologous vaccination group (●). Conversely, IL-5 secretion is a cytokine produced mainly by IM mRNA vaccination (●; Fig 4D and 4K). In this case, the heterologous IN NE/IVT/S primed (●) group secreted higher amounts of IL-5 than the heterologous IM mRNA primed vaccination group (●). These results again indicate that the boost immunization was dominantly influencing localization and Th bias, contrary to the author's conclusion. Even if you consider the intracellular cytokine staining data after Spike antigen stimulation, you can clearly see no difference in IL-17+ CD4+ T cells in the lungs of individuals who received heterologous IM mRNA primed (●) or heterologous IN NE/IVT/S (●) primed vaccinations (Fig. 3N). Taken together, the final conclusion reached by the authors were not supported by the data shown and hence I cannot recommend the acceptance of this manuscript for publication.

Response: These comments regarding Fig. 4 (now new Fig. 3) are also related to the previous comment regarding interpretation of the impact of sequence. We are in full alignment with all of the points raised by the reviewer in this sub-comment, and this also again is a problem with the way our conclusions were previously conveyed to simplify the message as prime dominance. However, boost dominance as the reviewer suggests also does not fully explain the observed data. As stated above, the message we want to convey is that immunization on an immune experienced baseline is not the same as on a naïve baseline, and that both the prime and boost play important roles in shaping the overall immune response. Indeed, as R2 pointed out, IM mRNA→IN NE/IVT/S led to higher IL-17A production in the spleen and cLN compared to the reverse IN NE/IVT/S→IM mRNA sequence, especially in the mucosal dLNs, which again supports this overall conclusion that even though these groups of mice both received two IN NE/IVT/S immunizations, the immune baseline set up by IM mRNA priming is important for bolstering systemic and especially, mucosal responses to the IN NE/IVT/S immunization. Thus, both the prime and boost influence these outcomes. In other words, the immune "baseline" induced after IM mRNA priming is not the same as that induced by IN NE/IVT/S priming, thus resulting in divergent responses to the booster vaccines compared to had they been administered on a naïve background. Importantly, all ICS data in Fig. 3 support this conclusion, with IFNg+, IFNg+IL2+TNFa+, and IL17A+ CD4s following the same pattern that IM mRNA priming followed by IN NE/IVT/S boosting enhances these responses and localizes them in the mucosa better than the reverse heterologous sequence. The reviewer does, however, point out the interesting exception to this for IL17A+CD4+ responses in the lung (now Fig. 3N) which were relatively equivalent between the IM mRNA→IN NE/IVT/S and the reverse IN NE/IVT/S→IM mRNA (while IN NE/IVT/S→IM mRNA showed minimal induction in the spleen and cLN). This may be

the result of the strong Th17 inducing properties of IN NE/IVT/S in the lungs even as a stand-alone homologous regimen (Fig. 3N) compared to the other cytokines. IN NE/IVT/S priming may thus establish lung-resident Th17 that can be boosted through systemic mediators which can reach the lung upon IM mRNA immunization. This indeed will be the subject of future investigation. We have now added a discussion of this to the results (on lines 380-385)

Similar concepts apply to IL5 which we also agree with the reviewer's statement regarding the connotation of prime vs. boost dominance. As NE/IVT is itself a Th1 promoting adjuvant, it also influences the bias of the overall induced TH response in addition to localization. We in fact see a reduction in IL5 induced by IM mRNA immunization in subjects primed with IN NE/IVT/S (IN NE/IVT/S→IM mRNA) compared to IM mRNA immunization on a naïve baseline, as well as minimal induction of IL-5 by the IN NE/IVT in the mucosal dLNs.

We greatly appreciate these useful comments and believe that they have helped us significantly improve our manuscript through our additional studies as well as in revising the messaging to most accurately convey the results.

Referee #3 (Comments on Novelty/Model System for Author):

R3: The statistical analysis of the data presented in Figure 1 is not correct.
The novelty is limited as previous studies have shown the benefit of parenteral prime and mucosal booster in the context of SARS-CoV-2 and other respiratory viral infections. However, the authors have provided an extensive and detailed analysis of the immune response.
The medical impact is modest as it seems unlikely that vaccine companies will provide two different types of vaccines for priming and boosting.
Response:

We thank Reviewer 3 for their time and consideration in carefully reviewing our manuscript and for the very helpful comments. We also appreciate the reviewer's encouraging comment on the extensive and detailed nature of the data presented. In response to these comments, we have since significantly restructured the paper and performed extensive additional experiments in efforts to improve the novelty of the findings to the field. These include: 1. making major overhaul revisions of the manuscript and figures with the goal of streamlining the data as well as the discussion of the data, 2. performing additional studies including tissue-level and systemic-level profiling of the differences in responses to IN or IM immunizations with mRNA or NE/IVT/S on naïve vs. immune-experienced baselines as well as identifying differences in immune cell infiltrates post-vaccination with these different vaccination regimens, and 3. performing additional mechanistic studies to identify why IM mRNA as a prime alters responses to subsequent mucosal vaccination most optimally, and 3. greatly expanding our profiling of the lung immune environment post-viral challenge and comparing differences between the different vaccination groups. Using unsupervised clustering analysis we identified significant differences in the types of immune cell infiltrates recruited during infection and their activation states-particularly within the myeloid cell compartment-based on vaccination history.

We believe these changes and additions have greatly improved the manuscript, and that our work now provides not only an effective vaccination strategy, but most importantly, foundational mechanistic insights into how prior immune experience influences later immune responses to subsequent vaccinations.

These foundational insights are applicable to not only thinking about how prior vaccination experience shapes boost responses, but also potentially about how infection shapes vaccination responses and vice versa. The impact of pre-existing immunity on vaccination is a rapidly growing area of interest, and thus, we believe this work not only highlights a unique vaccination system but also contributes important novel mechanistic additions to the field. With regards to the medical impact, we agree that licensure of two different vaccines for priming and boosting may be less lucrative to a company. However, while our main purpose was to demonstrate the basic fundamental differences underlying how vaccination sequences alter immune responses, and to better understand differences in vaccine responses in naïve vs. immune-experienced hosts, we believe this work actually is highly medically relevant as we demonstrate that IN NE/IVT (and potentially other IN vaccines) can be used as a booster to improve the immune responses (especially mucosal responses) induced by already licensed parenteral vaccines. We hope that the revisions and additions to our manuscript have now greatly improved the novelty and impact of the work.

Referee #3 (Remarks for Author):

The authors report on experiments investigating the effect of the sequence of immunization intramuscularly with an mRNA vaccine and intranasal with a nanoemulsion adjuvanted protein vaccine on the magnitude, quality and duration of the immune response. The overall conclusion is that intramuscular priming followed by intranasal booster generates the most effective and durable immune response.
The work aims to address a limitation of current vaccines against COVID-19 and other respiratory viruses, namely the lack of induction of an effective mucosal immune response. In addition, mRNA vaccines suffer from short duration of immunity and a boost with a protein subunit vaccine can potentially overcome this shortcoming. The idea that heterologous prime-boost (reviewed by S. Lu, Curr Opinion Immunol, 2009) and parental/mucosal administration strategies (e.g., Keren et al., Inf Immunity 1988) can enhance the immune response and provide better protection against mucosal pathogens is not new. This has also been shown in numerous studies for vaccines targeting SARS-CoV-2 only some of which are cited in the manuscript. It has also been shown that the sequence of parental/mucosal prime/booster affects the immune response (McCluskie et al., FEMS Immunology and Medical Microbiology, 2002). Thus, the novelty of the work lies in the combination of an mRNA vaccine with a mucosal nanoemulsion vaccine and the extensive and detailed analysis of the immune response. The authors are encouraged to perform a more thorough literature review and put their data in the context of published studies. The data support the overall conclusion that the sequence of priming and boosting has a significant effect on the immune response.

Response:

We thank the reviewer for these useful references and comments regarding the prime/pull approach. While it is indeed true that heterologous prime/boost or prime/pull approaches have been investigated by others, including the references the reviewer pointed out, the interest in this modality of vaccination has greatly surged with the renewed interest in driving long-lasting mucosal immunity. We believe that our findings-especially with all the newly added experimental data--go significantly deeper into characterizing the impact of chronological sequence in shaping the overall immune outcomes in different immune compartments within the systemic AND mucosal immune systems. Moreover, these other studies do not show induction of as durable mucosal immune responses as we have demonstrated herein.

Furthermore, we have now performed additional studies which demonstrate distinctions in innate responses and immune cell infiltrate profiles in response to these vaccinations systemically as well as locally within the tissue, and identified the initial systemic IFN-I responses during priming as being a critical component of promoting later mucosal immune responses to IN vaccination. We have completely revised our discussion section to more extensively put our work in context of what is known in the current literature, as well as incorporated more discussion of prior work including the references mentioned in the discussion as requested.

Other comments:

R3: 1. Figure 1 B - D and F - I. The legend states that antibody titers were compared by Mann-Whitney U-test. This is not appropriate for analysis of experiments with multiple groups. Instead, the authors should analyze log-transformed antibody titers by one-way ANOVA followed by an appropriate multiple comparison test. The Methods state that "titers were determined using a cutoff defined as the sum of the average absorbance at the lowest dilution of naïve serum and two times the standard deviation (line 1041-1043). It is not clear how this results in antibody titers of 1 for the PBS only and IN S only groups (same comment for Figure 6 B - D).

Response: We thank the reviewer for pointing out this error. We have now correctly analyzed the significance of the antibody titers by log10 transforming these antibody titers and using one-way ANOVA followed by Tukey's multiple comparisons test in these figures as well as in the SI figures. Furthermore, as R3 suggested, we have now defined "undetectable" titers as "1:25" for binding antibody titers and "1:30" for neutralizing antibody titers as this represents half of the value of where our dilution began at 1:50 and 1:60 dilutions for these assays, respectively. This information is now added to the figure legends as well as in the methods.

R3: 2. Figure 1 J, K. The graphs report OD readings instead of antibody titers as suggested in the Methods (lines 1035 - 1043). The dilution of the BAL fluid should be given.

Response: We have now included the dilution factor of the BAL (1:4) in the new methods under ELISA methods on line 1092.

R3: 3. Figure 1 (line 256). 3 - 20 mice/group. This is not reflected in the numbers depicted in the graphs.

Response: We apologize for the confusion of this statement. Panels B and C in Figure 1 have larger sets of mice in their respective groups post prime and post prime/boost which were then randomized into different treatment groups as the immunization regimen progressed. We have corrected the Figure 1 legend to more accurately reflect the mice in each group as the regimen progresses.

R3: 4. All figure legends. Please add a sentence of what the bars show (mean/geometric mean, SD or SEM).

Response: We have added this information to the legends for all figures and/or the Y axis of the plots themselves.

R3: 5. Line 307-309. "no notable differences in the overall frequencies of total CD8+ or CD4+ T cells in the cLN were observed between groups...". Presumably the lymph nodes of immunized mice were enlarged and contained more lymphocytes than control lymph nodes. A more meaningful indication would be the total number (absolute instead of percentage) of antigen-specific CD4 and CD8 T cells.

Response: We have now switched these analyses to provide absolute numbers of CD4 and CD8 T cells in Fig. S2 as well as revised this in the indicated section in the results.

6. Figure 3D and 4F. There is an increase of IL-17 secretion in certain groups of mice that was not seen by intracellular cytokine staining. This suggests that cells other than CD4 T cells contribute to the IL-17 secretion. This should be discussed.
Response: We thank the reviewer for pointing this out. Indeed, the differences between the groups R3 is referring to (now new Figs. 3D, F) in the spleen for IL17A by ICS are much more pronounced than by Luminex. However, these assays are different in their nature, as ICS is a snapshot of cytokine production and was performed after 24h of stimulation, while the Luminex is a cumulative assessment of cytokine production over 72h of stimulation. While the heterologous IM mRNA→IN NE/IVT/S group induces significantly higher responses than the IN NE/IVT/S→IM mRNA and homologous IN NE/IVT/S groups, the IL17+CD4+T cells in the ICS for the groups R3 points out (IN NE/IVT/S→IM mRNA and homologous IN NE/IVT/S) are not zero, reflecting the presence of TH17 cells in these treatment groups. While other cell types including gamma delta T cells can also possibly be contributing IL17A, we found negligible IL17A+ CD4- cells in our ICS results. We have now added this statement to the results on lines 314-316.

R3: 7. Line 910. "Delays in vaccination is a significant problem with mRNA vaccines." The genesis of this statement is not clear.
8. Lines 960-961. The correct names of the mouse strains are C57BL/6J and B6.Cg-Tg(K18-ACE2)2Prlmn/J (which can be abbreviated to K18-hACE2 in other parts of the manuscript) and the source is presumably The Jackson Laboratory.

Response: We thank the reviewer for pointing this statement out and have removed this statement in our major revision of the text. We have also fixed the mouse strains (lines 1048-1049) to indicate the full name on first usage as suggested.

We greatly appreciate these comments from Reviewer 3 and hope that our revisions and new experimental data now included has increased the novelty of the work, distinguishing it from other studies. We believe that it offers important mechanistic insights into why sequence matters so much in heterologous vaccine regimens, and provides fundamental information regarding how pre-existing immune baselines can alter subsequent vaccination responses.

3rd Jul 2026

Dear Dr. Wong,

Thank you for submitting your revised manuscript. Following the rejection of your original submission after peer review, you appealed the decision and submitted a revised manuscript addressing the referees' initial comments. We carefully considered your appeal and concluded that the revised manuscript warranted re-review. As Referee #2 was unfortunately unavailable to assess the revised manuscript, we invited two additional reviewers.

Please accept my apologies for the delay in our response, as one referee required additional time to complete their evaluation.

As you will see from the reports below, Referee #3, who also reviewed the original submission, is now broadly supportive of the manuscript, subject to additional revisions. Referee #5 is also supportive of publication, provided that the remaining concerns raised in their report are satisfactorily addressed. In contrast, Referee #4 raises more substantial concerns, particularly regarding the study's novelty and its potential medical impact.

We have carefully considered the reviewers' reports and discussed the manuscript extensively within the editorial team and with the referees. While we recognize that the study may not have immediate clinical relevance, we believe it provides important mechanistic insights with potential translational implications. Moreover, the remaining concerns appear addressable within a reasonable timeframe. We would therefore like to invite you to submit a further revised version of the manuscript.

In preparing your revision, please address all remaining reviewer concerns carefully, including through additional experiments where necessary, and ensure that the claims and conclusions are appropriately balanced and not overstated.

Please note that, at EMBO Press, we generally allow only one round of major revisions. Accordingly, this will represent the final opportunity to address the outstanding issues raised during peer review.

If you would like to discuss the reviewers' comments or our editorial expectations before preparing your revision, I would be happy to do so by email or via a video call. Please let me know if this would be helpful.

When preparing your revised manuscript, please refer to our guidelines: https://link.springer.com/journal/44321/submission-guidelines#cms-Revised-submissions. We perform an initial quality control of all revised manuscripts before re-review; failure to include requested items will delay the evaluation of your revision.

We require:

1) A .docx formatted version of the manuscript text (including legends for main figures, EV figures and tables). Please make sure that the changes are highlighted to be clearly visible.

2) Individual production quality figure files as .eps, .tif, .jpg (one file per figure). For guidance, download the 'Figure Guide PDF': https://media.springernature.com/original/springer-cms/rest/v1/content/27825798/data/v1.

3) A .docx formatted letter INCLUDING the reviewers' reports and your detailed point-by-point responses to their comments. As part of the EMBO Press transparent editorial process, the point-by-point response is part of the Review Process File (RPF), which will be published alongside your paper.

4) A complete author checklist, which you can download from our author guidelines. Please insert information in the checklist that is also reflected in the manuscript. The completed author checklist will also be part of the RPF.

5) Please note that all corresponding authors are required to supply an ORCID ID for their name upon submission of a revised manuscript.

6) It is mandatory to include a 'Data Availability' section after the Materials and Methods. Before submitting your revision, primary datasets produced in this study need to be deposited in an appropriate public database, and the accession numbers and database listed under 'Data Availability'. Please remember to provide a reviewer password if the datasets are not yet public.

In case you have no data that requires deposition in a public database, please state so in this section. Note that the Data Availability Section is restricted to new primary data that are part of this study.

7) For data quantification: please specify the name of the statistical test used to generate error bars and P values, the number (n) of independent experiments (specify technical or biological replicates) underlying each data point and the test used to calculate p-values in each figure legend. The figure legends should contain a basic description of n, P and the test applied.

Graphs must include a description of the bars and the error bars (s.d., s.e.m.).

8) At EMBO Press we ask authors to provide source data for the main manuscript figures. You will receive a separate email with instructions for providing source data with your revised manuscript, including how to upload and organize the files.

9) Our journal encourages inclusion of *data citations in the reference list* to directly cite datasets that were re-used and obtained from public databases. Data citations in the article text are distinct from normal bibliographical citations and should directly link to the database records from which the data can be accessed. In the main text, data citations are formatted as follows: "Data ref: Smith et al, 2001" or "Data ref: NCBI Sequence Read Archive PRJNA342805, 2017". In the Reference list, data citations must be labeled with "[DATASET]". A data reference must provide the database name, accession number/identifiers and a resolvable link to the landing page from which the data can be accessed at the end of the reference.

10) We replaced Supplementary Information with Expanded View (EV) Figures and Tables that are collapsible/expandable online. A maximum of 5 EV Figures can be typeset. EV Figures should be cited as 'Figure EV1, Figure EV2" etc... in the text and their respective legends should be included in the main text after the legends of regular figures.

- For the figures that you do NOT wish to display as Expanded View figures, they should be bundled together with their legends in a single PDF file called *Appendix*, which should start with a short Table of Content. Appendix figures should be referred to in the main text as: "Appendix Figure S1, Appendix Figure S2" etc.

- Additional Tables/Datasets should be labeled and referred to as Table EV1, Dataset EV1, etc. Legends have to be provided in a separate tab in case of .xls files. Alternatively, the legend can be supplied as a separate text file (README) and zipped together with the Table/Dataset file.

11) The paper explained: EMBO Molecular Medicine articles are accompanied by a summary of the articles to emphasize the major findings in the paper and their medical implications for the non-specialist reader. Please provide a draft summary of your article highlighting
- the medical issue you are addressing,
- the results obtained and
- their clinical impact.

This may be edited to ensure that readers understand the significance and context of the research. Please refer to any of our published articles for an example.

12) Author contributions: You will be asked to provide CRediT (Contributor Role Taxonomy) terms in the submission system. These replace a narrative author contribution section in the manuscript.

13) A "Disclosure and Competing Interests Statement" should be provided in the main text.

14) Every published paper includes a 'Synopsis' to further enhance discoverability. Synopses are displayed on the journal webpage and are freely accessible to all readers. They include a short stand first (maximum of 300 characters, including space) as well as 2-5 one-sentences bullet points that summarizes the paper. Please write the bullet points to summarize the key NEW findings. They should be designed to be complementary to the abstract - i.e. not repeat the same text. We encourage inclusion of key acronyms and quantitative information (maximum of 30 words / bullet point). Please use the passive voice. Please attach these in a separate file or send them by email, we will incorporate them accordingly.

Please also suggest a striking image or visual abstract to illustrate your article as a PNG file 550 px wide x 300 px high.

15) Include a Reagents and Tools Table as part of the Methods section, which can be downloaded from our author guidelines.

16) As part of the EMBO Publications transparent editorial process initiative (see our Editorial at http://embomolmed.embopress.org/content/2/9/329), EMBO Molecular Medicine will publish online a Review Process File (RPF) to accompany accepted manuscripts.
In the event of acceptance, this file will be published in conjunction with your paper and will include the anonymous referee reports, your point-by-point response and all pertinent correspondence relating to the manuscript. Let us know whether you agree with the publication of the RPF and as here, if you want to remove or not any figures from it prior to publication.
Please note that the Authors checklist will be published at the end of the RPF.

EMBO Molecular Medicine has a "scooping protection" policy, whereby similar findings that are published by others during review or revision are not a criterion for rejection. Should you decide to submit a revised version, I do ask that you get in touch after three months if you have not completed it, to update us on the status.

I look forward to seeing a revised form of your manuscript as soon as possible.

With kind regards,

Lise

Lise Roth, Ph.D
Senior Editor
EMBO Molecular Medicine

***** Reviewer's comments *****

Referee #3 (Comments on Novelty/Model System for Author):

This is overall a high quality manuscript with an extensive data set. I am concerned about the validity of data presented in Figure 6 (see my comment 4. to the authors).
The medical impact is low, because I don't think that the heterologous prime-boost vaccination strategy will be implemented in clinical practice any time soon. However, the work does contribute to our overall understanding of the immune response to vaccination, in particular against respiratory viruses.

Referee #3 (Remarks for Author):

The authors have made substantial revisions to the manuscript and overall improved its quality. A few issues remain as the authors sometimes overstate their findings and do not always sufficiently address limitations of their experimental work.

1. Line 326 - 334. The authors should focus on the data that are statistically significant. There was no significant difference in the IL-5 and IL-13 concentrations in the IM-PBS-IM vs IM-IN-IN groups and between the IM-IN-IN vs IN-IN-IM groups. This does not support the statement that "IM mRNA used as a boost to the mucosal prime shifts the primed immune response towards Th2 responses". It is also not clear what the "singly IM mRNA" group (line 330) refers to.
2. Line 336-337. "with heterologous IM mRNA-IN NE/IVT/S generating more IL-17A than homologous IN NE/IVT/S or heterologous vaccination with IN NE/IVT/S priming". The data (Fig. 3F) do not show a difference between these groups.
3. In line 447, the authors refer to the IN-IN-IM group as "heavily Th2 polarized". This is not supported by the data as discussed under comment 1.
4. Figure 6E, Lines 651-658, Lines 941-942. The authors report little or no infiltration of inflammatory cells in muscle at 24 h after mRNA injection. This would be unexpected and is not consistent with the published literature (e.g., Liang et al., Molecular Therapy 2017; Hassett et al., Molecular Therapy Nucleic Acids, 2024; Meany et al., Science Advances, 2025). I suspect that this is because of a technical issue (missing the injection site) and not actually true. Also, the data (Fig. 6D and E) are listed as Neutrophils % and Monocytes % - % of what?
5. Line 638-639. "markedly higher recruitment of Ly6C+monocytes to the nasal mucosa than the reverse IN NE/IVT/S-IM mRNA sequence." and line 944-945. "In contrast, MHCII+ expressing Ly6C+monocytes were markedly reduced with the reverse IN NE/IVT/S-IM mRNA." This difference is likely at least in part be due to the timing, i.e. 4 weeks (IN prime - IM boost) vs 24h (IM prime - IN boost) after the last intranasal immunization as suggested by the higher % of neutrophils and monocytes in the PBS prime - IN boost group vs the IN prime - IM boost group. The authors should take this in consideration for the interpretation of all differences (cell types, chemokines, cytokines, etc) between groups in the nasal mucosa.

Referee #4 (Comments on Novelty/Model System for Author):

The statistical tests and animal models are appropriate.
The novelty is low since many groups have reported the results of heterologous immunizations. The medical impact of the work is low since IN followed by IM immunization is not a likely clinical scenario.

Referee #4 (Remarks for Author):

This manuscript reports the effects of heterologous immunization with WT SARS CoV-2 protein, adjuvanted with a nanoparticle, and compares the immunogenicity and protection to an approved vaccine. Different immunization routes are tested and cellular and humoral immune responses are reported.

Major concerns:

The amount of S protein used for immunization is very high (listed as 20 mcg in the results and 15 mcg in the methods, thus there is discrepancy). Most studies in mice use 1 mcg or less of S protein. Comparing the results with animals immunized with less than 2 mcg of mRNA vaccine is difficult. The rationale for the choice of vaccine quantities is not discussed.

A key weakness of this study is that heterologous immunization routes have been reported by many other groups and the data presented here do not add anything new to the literature. Furthermore, IN prime followed by IM booster is not a likely clinical scenario and thus this comparison is unclear.

Most of the population has received multiple boosters with vaccines containing variant S protein; thus, use of the WT S is outdated, and the rationale for evaluating the booster responses in the background of a systemic prime with WT S is invalid.

Reduction of viral load to the LOD in most of the immunization groups by day 2 in the nose is likely due to the non-physiological amount of S protein used for immunization.

Evaluation of responses in lymph nodes is insufficient to show that tissue-resident T cell responses are generated in the mucosa. Tetramers were used in lung tissue, but not in the nose, where it is important to detect mucosal responses. Cytokine responses by flow cytometry in mucosal tissues was not conducted; evaluation of cytokines in homogenates does not determine whether the responses were generated by the antigen-specific CD8 and CD4 T cells. Evaluation by intracellular cytokine staining is the accepted method.

Referee #5 (Remarks for Author):

This study analyzed the effects of different vaccine forms and vaccination sequences on systemic and mucosal immune responses, using multiple vaccination sequences such as intramuscular injection first (mRNA vaccine), followed by mucosal (subunit vaccine nasal drop), or mucosal vaccination followed by intramuscular injection, to investigate the immune response produced by SARS-CoV2 vaccines. It was found that different sequences lead to distinctly different Th biases (such as Th1/Th2/Th17 balance) and antibody subtype distributions, revealing that the type I interferon (IFN-I) signaling induced by initial intramuscular mRNA vaccines remodels the innate immune microenvironment locally (injection site) and throughout the body (such as the spleen). This "pre-stimulation state" allows subsequent mucosal inoculation to be more efficiently "amplified," thereby explaining the molecular mechanisms of sequencing dependence and providing immunological basis for vaccination strategies for respiratory infectious disease vaccines, which is found to be the most difficult scientific questions and reviewed recently (Wang et al. Science Bulletin 2026 PMID: 42150938). There are some questions about this study that are still recommended for further discussion or analysis, though the questions raised by the previous round of review has been addressed.

Major comments:

1. This "prime-pull" strategy has been widely reported. Based on this, this study compared different immune sequences and highlighted the important role of IFN-I signaling during priming. The authors blocked the effect of the "prime-pull" with IFN-I antibodies. It is worth to analyze if I subunit vaccines are directly injected into the muscle accompanied with IFN-I injection, then subunit vaccine nasal drops can enhance systemic immunity from mucosal nasal drops? It is recommended that the authors supplement related experiments to further demonstrate the important role of IFN-I signaling in the "prime" process, if time permits. Or at least this should be discussed.

2. On the other hand, if type II interferon signals are blocked, for example, does adding anti-IFN- affect the immune response of "prime-pull" strategy? (Since Th1 cells producing IFN- may also affect prime boost effects). Again, at least this should be discussed if workload is time-consuming.

3. In Fig. 6A, compared to IN+IM group, serum IFN- levels in IM are significantly higher than those in IM+IN group, but the authors believe IFN- levels are crucial for vaccine response induced by IM+IN. How can this inconsistency be explained? Please discuss.

4. In Fig.6, TNF and CCL2 levels induced by IN+IM group are significantly lower than those of IM+IN group. Do these two signals depend on IFN-I? It is generally believed that TNF signals are generated by TLR-related PAMP signals, while IFN-I is generated by RIG-I signals and are not related to upstream or downstream processes. Do TNF and CCL2 signals positively regulate the prime boost effect?

Minor comments:

1. I suggest the author revise the title of each figure, some are too long (such as Fig. 5 and Fig. 7) In Fig. 6, title is missing.
2. In Fig. 5M, the IL-17A "+" should be superscript.
3. In Fig. 6, please standardize the gene names, like Il1b and Tnf.

***** Reviewer's comments *****

Referee #3 (Comments on Novelty/Model System for Author):

This is overall a high quality manuscript with an extensive data set. I am concerned about the validity of data presented in Figure 6 (see my comment 4. to the authors).
The medical impact is low, because I don't think that the heterologous prime-boost vaccination strategy will be implemented in clinical practice any time soon. However, the work does contribute to our overall understanding of the immune response to vaccination, in particular against respiratory viruses.

We thank the reviewer for reviewing our manuscript again and very much appreciate these encouraging comments that our efforts to revise the manuscript have significantly improved it. We also thank the reviewer for these additional valuable comments and have made the suggested revisions accordingly within the text and addressed the comments as detailed below, and hope that it is now acceptable for publication.

Referee #3 (Remarks for Author):

The authors have made substantial revisions to the manuscript and overall improved its quality. A few issues remain as the authors sometimes overstate their findings and do not always sufficiently address limitations of their experimental work.

1. Line 326 - 334. The authors should focus on the data that are statistically significant. There was no significant difference in the IL-5 and IL-13 concentrations in the IM-PBS-IM vs IM-IN-IN groups and between the IM-IN-IN vs IN-IN-IM groups. This does not support the statement that "IM mRNA used as a boost to the mucosal prime shifts the primed immune response towards Th2 responses". It is also not clear what the "singly IM mRNA" group (line 330) refers to.

Response: We agree these differences were small for IL13 and also agree that indeed the IL5 changes were not statistically significant by one-way ANOVA with the group size. We believe, however, the IL5 differences are biologically relevant as the differences between the IM-PBS-IM and the IN-IN-IM groups vs. the IM-IN-IN group were substantial (mean levels being ~2000 and 1000 pg/mL vs. 350 pg/mL, respectively, and the highest IL5 levels being ~ 5600 and 2500 pg/mL vs. 600pg/mL, respectively). This may have been harder to distinguish due to the position of our axis break. We have adjusted this in Fig. 3F to better illustrate this difference and have also adjusted the text in this section to temper the statement accordingly on lines: 310-321 (markup version). We also thank the reviewer for pointing out the typo on (initial) line 330. We have removed the “singly IM mRNA” in this sentence (line 315 of markup version).

2. Line 336-337. "with heterologous IM mRNA-IN NE/IVT/S generating more IL-17A than homologous IN NE/IVT/S or heterologous vaccination with IN NE/IVT/S priming". The data (Fig. 3F) do not show a difference between these groups.

Response: We agree that while IL17A trends higher in the IM-IN group in the spleen compared to the IN-IM group (as it also does in the cLN), there is spread and it is not statistically significant. Thus, we have removed this statement (lines 323-325 of markup version).

3. In line 447, the authors refer to the IN-IN-IM group as "heavily Th2 polarized". This is not supported by the data as discussed under comment 1.

Response: As in our comment 1 response, we have removed "heavily" to tone down this statement.

4. Figure 6E, Lines 651-658, Lines 941-942. The authors report little or no infiltration of inflammatory cells in muscle at 24 h after mRNA injection. This would be unexpected and is not consistent with the published literature (e.g., Liang et al., Molecular Therapy 2017; Hassett et al., Molecular Therapy Nucleic Acids, 2024; Meany et al., Science Advances, 2025). I suspect that this is because of a technical issue (missing the injection site) and not actually true. Also, the data (Fig. 6D and E) are listed as Neutrophils % and Monocytes % - % of what?

We appreciate the reviewer for pointing this out and have thoroughly reviewed these manuscripts suggested by the reviewer and have also looked at other work as well. We agree that these responses are lower compared to some of these references. However, we do not believe this is due to a technical issue of missing the injection site, as we have performed characterization of the surrounding muscle for intramuscular vaccines and see similar changes in infiltration mirrored within the hamstring as in the quadriceps. Furthermore, while our interests were mostly on the neutrophils, and inflammatory monocytes, we did see increased infiltration of other cell types (not shown) after IM mRNA immunization including pDCs which is in line with the induction of IFNI pathways we show in the muscle in Figure 6C. We noted that while these papers also demonstrate an increase in infiltrates in the muscle, much of the influx of certain cell types, especially neutrophils, is already on the downward trajectory at the 24h time point as the response shifts to the draining lymph nodes. This is also shown in Dowell et al., ACS Nano 2024 with the Moderna mRNA vaccine (vs. our study Pfizer mRNA vaccine). This is the time point which we chose for our sampling of the injection site in Figure 6 as a compromise, as we wanted to also capture infiltration in the dLNs (Fig. EV4). Some of the differences between these provided references may be due to this somewhat "later" time point as well as differences in the mRNA vaccines used (different LNPs, different mRNA dosages, different antigen (Hassett et al.) or different formulation (adjuvanted hydrogel (Meany et al.)) as well as a different species (NHPs (Liang et al)). Figs. 6D and E neutrophil % and monocyte % are of total live singlet cells. We thank the reviewer for noting this point of confusion and have clarified the figure legend accordingly to better clarify this.

5. Line 638-639. "markedly higher recruitment of Ly6C+monocytes to the nasal mucosa than the reverse IN NE/IVT/S-IM mRNA sequence." and line 944-945. "In contrast, MHCII+ expressing Ly6C+monocytes were markedly reduced with the reverse IN NE/IVT/S-IM mRNA." This difference is likely at least in part be due to the timing, i.e. 4 weeks (IN prime - IM boost) vs 24h (IM prime - IN boost) after the last intranasal immunization as suggested by the higher % of neutrophils and monocytes in the PBS prime - IN boost group vs the IN prime - IM boost group. The authors should take this in consideration for the interpretation of all differences (cell types, chemokines, cytokines, etc) between groups in the nasal mucosa.

Response: We appreciate this insightful comment as the timing interval between the last IN immunization indeed could influence these differences in part, as the recruitment of Ly6C+ monocytes is typically a transient effect occurring shortly after administration of vaccine. Therefore, the reviewer makes a good point that this observation may be impacted by the different time intervals between last intranasal booster and time of analysis for the IN prime-IM boost vs. IM prime-IN boost (4 wks vs 24hrs, respectively). However, we also note that comparing the IM mRNA→IN NE/IVT/S vs. IN NE/IVT/S→IN NE/IVT/S groups (which received the IN NE/IVT/S intranasal boost at the same time point) shows higher Ly6C+ monocyte recruitment with the heterologous IM mRNA primed, IN NE/IVT/S boosted mice compared to homologous intranasal vaccination only IN NE/IVT/S→IN NE/IVT/S group (Fig. 6D middle). Therefore, this suggests that IM mRNA priming enhances the recruitment of Ly6C+ monocytes to the mucosa after IN NE/IVT/S booster, as compared to the animals that received intranasal vaccination only and that this is not simply an artifact of timing. We have also now mentioned this in the revised discussion section lines 584-595 of markup version. Similarly, the singly immunized group IN PBS→IN NE/IVT/S which also received the IN immunization at the same time interval (24h before analysis) showed reduced levels of cytokines in the nasal tissue compared to the IM mRNA→IN NE/IVT/S and IN NE/IVT/S→IN NE/IVT/S, demonstrating the impact of previously established immunity on the overall outcomes (Fig. 6B (IL1b, TNFa, CCL2)). Discussion of this has now been emphasized in the text.

Referee #4 (Comments on Novelty/Model System for Author):

The statistical tests and animal models are appropriate.
The novelty is low since many groups have reported the results of heterologous immunizations. The medical impact of the work is low since IN followed by IM immunization is not a likely clinical scenario.

Referee #4 (Remarks for Author):

This manuscript reports the effects of heterologous immunization with WT SARS CoV-2 protein, adjuvanted with a nanoparticle, and compares the immunogenicity and protection to an approved vaccine. Different immunization routes are tested and cellular and humoral immune responses are reported.

We thank the reviewer for their time in reviewing our manuscript and for pointing out certain areas of potential unclarity which we have adjusted accordingly. We hope these changes have improved the manuscript. Our responses are detailed below.

Major concerns:

1. The amount of S protein used for immunization is very high (listed as 20 mcg in the results and 15 mcg in the methods, thus there is discrepancy). Most studies in mice use 1 mcg or less of S protein. Comparing the results with animals immunized with less than 2 mcg of mRNA vaccine is difficult. The rationale for the choice of vaccine quantities is not discussed.

Response: To achieve robust immune responses through the intranasal route, significantly higher amounts of recombinant antigen are required for administration compared to the intramuscular route. Thus, 15ug-20ug is not generally considered high in the field for IN vaccination with recombinant protein antigens.

Furthermore, for SARS-CoV-2, there are predominantly two stabilized constructs of spike protein used in vaccination studies which are often used interchangeably (and sometimes not distinguished in the methods). Herein we used the S2P version of spike protein which corresponds to the same stabilized version of the spike protein encoded by the mRNA vaccine (contains 2 proline substitutions). We have indicated this in the appendix supplemental methods (adjuvants and antigens section). Some groups exploring prime/pull approaches or IN alone approaches have used the hexapro version of the spike protein which contains six proline substitutions. This version has improved stability but also is much more immunogenic. We chose the S2P version herein as it matches the mRNA used and allows us to better distinguish differences between treatment groups. We have added this sentence for selection rationale at the end of this paragraph in the appendix supplemental methods (adjuvants and antigen section). The dose of S protein was reduced to 15ug for the challenge study and the IFNAR blocking study to better distinguish differences in protection and dependency on IFNIs. We have indicated this in the immunization section in the main methods section, and we have added further clarification at the end of the paragraph in the methods in the "Characterization of IFN-I signaling at priming in heterologous immunization" section. In contrast, the typical dose of the BNT162b2 mRNA vaccine standardized from human dosing adjusted to mice is typically 1-2 ug.

2. A key weakness of this study is that heterologous immunization routes have been reported by many other groups and the data presented here do not add anything new to the literature. Furthermore, IN prime followed by IM booster is not a likely clinical scenario and thus this comparison is unclear.
Most of the population has received multiple boosters with vaccines containing variant S protein; thus, use of the WT S is outdated, and the rationale for evaluating the booster responses in the background of a systemic prime with WT S is invalid.

We appreciate the reviewer's comment regarding translatability. While indeed most of the population has received multiple IM boosters for COVID19, our primary goal in this manuscript is to demonstrate that vaccination on a naïve background is not the same as vaccination on an immune-experienced baseline. We respectfully believe that this is a highly clinically relevant and significant topic. This is especially pertinent with emerging studies demonstrating the impact of pre-existing immunity on shaping both downstream innate and adaptive immunity. Thus, we feel that the question of how route of prior immune priming influences later boost responses, and understanding the fundamental mechanisms behind these differences have significant importance not only to heterologous vaccination regimen development but also have significant implications for understanding how mucosal infections/exposures interplay with systemic vaccinations through adaptive and trained immunity. We also agree that SARS-CoV-2 has evolved far beyond the WT S, however, this was used as our model antigen given the immunological tools that are readily available (mRNA, tetramers, validated animal challenge models etc).

3. Reduction of viral load to the LOD in most of the immunization groups by day 2 in the nose is likely due to the non-physiological amount of S protein used for immunization.

Response: We agree that even more suboptimal doses of S protein and mRNA would provide greater differences in the viral load readouts for these groups. In the challenge we used lower dosing for the S protein (see comment 1) to better distinguish differences between groups. The unadjuvanted S protein given IN alone followed by IM mRNA boosting gave detectable viral loads in both the lungs and nasal

turbinates, demonstrating that this dose of S protein is not sufficient for protection without adjuvant, and that this level of S protein is not overtly high. Our main point in this figure is that even though protection in these groups was achieved, the profile of protective responses varied significantly based on immunization regimen and sequence.

4. Evaluation of responses in lymph nodes is insufficient to show that tissue-resident T cell responses are generated in the mucosa. Tetramers were used in lung tissue, but not in the nose, where it is important to detect mucosal responses. Cytokine responses by flow cytometry in mucosal tissues was not conducted; evaluation of cytokines in homogenates does not determine whether the responses were generated by the antigen-specific CD8 and CD4 T cells. Evaluation by intracellular cytokine staining is the accepted method.

Response: We thank the reviewer for these comments. We strongly agree that mucosal T cell responses should be evaluated both at the levels of the mucosal draining LNs as well as within the mucosal tissue (tissue-resident T cell responses). Accordingly, we did actually perform tetramer staining to quantify S-specific T cells in the draining LNs, the lung tissue, as well as in the mucosal tissue of the "nose" within the nasal-associated lymphoid tissue (NALT) Figs. 2D, E, G, H. Furthermore, besides the cytokine measurements in lung homogenate post-challenge as mentioned by the reviewer, we did indeed perform antigen-specific stimulation and intracellular staining for T cell responses in vaccinated-not challenged mice within both the mucosal draining LNs (cLNs) and the mucosal tissue (lung) in Figs. 3G-J, and L-O, respectively to assess recall responses.

Referee #5 (Remarks for Author):

We greatly appreciate the reviewer for these very insightful comments and for taking the time to review our work thoroughly. We have attempted to address these comments to the best of our ability and include new discussion and explanations in the manuscript accordingly. We believe that these additions have greatly improved the overall quality of the paper.

This study analyzed the effects of different vaccine forms and vaccination sequences on systemic and mucosal immune responses, using multiple vaccination sequences such as intramuscular injection first (mRNA vaccine), followed by mucosal (subunit vaccine nasal drop), or mucosal vaccination followed by intramuscular injection, to investigate the immune response produced by SARS-CoV2 vaccines. It was found that different sequences lead to distinctly different Th biases (such as Th1/Th2/Th17 balance) and antibody subtype distributions, revealing that the type I interferon (IFN-I) signaling induced by initial intramuscular mRNA vaccines remodels the innate immune microenvironment locally (injection site) and throughout the body (such as the spleen). This "pre-stimulation state" allows subsequent mucosal inoculation to be more efficiently "amplified," thereby explaining the molecular mechanisms of sequencing dependence and providing immunological basis for vaccination strategies for respiratory infectious disease vaccines, which is found to be the most difficult scientific questions and reviewed recently (Wang et al. Science Bulletin 2026 PMID: 42150938). There are some questions about this study that are still recommended for further discussion or analysis, though the questions raised by the previous

round of review has been addressed.

Major comments:

1. This "prime-pull" strategy has been widely reported. Based on this, this study compared different immune sequences and highlighted the important role of IFN-I signaling during priming. The authors blocked the effect of the "prime-pull" with IFN-I antibodies. It is worth to analyze if I subunit vaccines are directly injected into the muscle accompanied with IFN-I injection, then subunit vaccine nasal drops can enhance systemic immunity from mucosal nasal drops? It is recommended that the authors supplement related experiments to further demonstrate the important role of IFN-I signaling in the "prime" process, if time permits. Or at least this should be discussed.

We agree that this is a very interesting point to examine whether IFNI signaling alone is sufficient for this conditioning or whether this prime-pull IFNI driven effect is unique to the mRNA platform. While IFN-I given mucosally with, or given IP preceding mucosal vaccination has been shown to enhance mucosal responses to vaccination, the IFN-I response post-IM mRNA immunization clearly diminishes by the booster time point. Although we have not directly performed co-parenteral administration of IFNI with the subunit vaccine, we have performed similar prime/pull vaccination studies in the past using parenteral adjuvants such as MF59 (Addavax) with recombinant antigen. This adjuvant does not trigger as robust of an IFNI response as the mRNA vaccines, and accordingly upon IN NE/IVT/S boost showed significantly lower enhancement of mucosal immunity than observed here with mRNA priming. While other differences in the mRNA platform also include the longer duration of antigen production/persistence within the site of injection compared to subunit antigens which likely also contributes in part to the magnitude of this effect, IFN-I induction has been shown to induce an innate memory-like state through epigenetic reprogramming, which leads to enhancement of downstream responses to vaccination for certain vaccines. Further investigation is currently underway into the differences trained immune response signatures induced by the regimens in this study and how they impact the synergistic mucosal response observed. We have now included discussion of this in lines 879-889 of the markup version.

2. On the other hand, if type II interferon signals are blocked, for example, does adding anti-IFN-γ affect the immune response of "prime-pull" strategy? (Since Th1 cells producing IFN-γ may also affect prime boost effects). Again, at least this should be discussed if workload is time-consuming.

We also agree that examining the role of IFN-γ in promoting the immune responses we observe with the prime-pull strategy is a valuable factor to examine. Type I IFNs and IFN-γ are tightly intertwined, with induction of type I IFNs strongly promoting/enhancing and regulating the production of IFN-γ. Furthermore, IFN-I induction during priming has been seen to epigenetically program newly formed T cells, priming them to be more capable of secreting larger amounts of IFN-γ in response to booster vaccination. Rapid local IFN-γ production by antigen-specific Th1 cells during a boost has been shown (through its induction of chemokine production in the mucosal tissue) to facilitate recruitment of circulating immune cells into the mucosal tissue, including innate immune cells such as monocytes, as well as helping to recruit and fortify residency of resident memory T cells in the mucosa. Further, $CD4^+$ T_{RM}s enhance local immune responses by secreting IFN-γ, promoting the formation of promoting the formation $CD4^+$ T_{RM}s as well as BRM's. As such we would hypothesize that IFN-γ also plays a critical role in promoting mucosal responses by the heterologous vaccination strategy. Since IFNI acts upstream

of IFNg we focused our studies here on blocking IFNI signaling given the already large scope of our work. However, we will include this valuable suggestion in future studies to dissect which components are specifically due to IFN-γ induced by vaccination and now include discussion of this in the manuscript in lines 889-898 of the markup version.

In Fig. 6A, compared to IN+IM group, serum IFN-α levels in IM are significantly higher than those in IM+IN group, but the authors believe IFN-α levels are crucial for vaccine response induced by IM+IN. How can this inconsistency be explained? Please discuss.

We thank the reviewer for raising this point. Indeed the serum IFN-a levels in the singly IM mRNA immunized IN PBS→IM mRNA and the homologous IM mRNA prime/boost groups are higher than that induced by the IN NE/IVT/S→IM mRNA group after the boost immunization. We apologize for any confusion. To better clarify, our conclusions were that IFN-a induced during the prime leads to the establishment of an immune environment which is conducive to later enhanced mucosal responses to mucosal vaccination during the boost phase. In other words IFNa during the prime sets the stage for these optimal mucosal responses we observed upon boosting intranasally. Furthermore, we conclude that in order for this to occur, the induced IFNα burst has to precede the mucosal vaccination, which is why the sequence of vaccination impacted the overall response outcomes significantly. We have tried to further emphasize this on lines 659-661 of the markup version. The serum cytokines shown in figure 6A were measured 24h after the boost. Thus, the high IFN-a induced by IN PBS→IM mRNA and IM mRNAx2 led to enhanced mucosal responses after IN NE/IVT/S boosting (earlier figures), and it may be the case that additional boosting of the IN NE/IVT/S→IM mRNA group with another IN NE/IVT/S final boost (IN NE/IVT/S→IM mRNA→IN NE/IVT/S) may give enhancement in mucosal responses.

4. In Fig.6, TNF and CCL2 levels induced by IN+IM group are significantly lower than those of IM+IN group. Do these two signals depend on IFN-I? It is generally believed that TNF signals are generated by TLR-related PAMP signals, while IFN-I is generated by RIG-I signals and are not related to upstream or downstream processes. Do TNF and CCL2 signals positively regulate the prime boost effect?

We appreciate the reviewer for bringing up this interesting point. CCL2 is expression is upregulated by IFNI signaling, driving the recruitment of inflammatory monocytes. This was consistent with what we observed in the nasal tissue, with the IM+IN and the IN+IN groups also resulting in the highest frequency of Ly6C+ monocytes, and the low CCL2 inducing IN+IM group showing the least amount of Ly6C monocyte recruitment compared to these groups. Indeed, we believe this recruitment of inflammatory monocytes is an important component driving the prime/boost effect as we discuss in the manuscript. The TNFa induction observed is also very interesting as it is consistent with our combined NE/IVT adjuvant which is designed to target both TLRs as well as RIG-I to result in an innate immune response more closely emulating natural viral infection in the mucosa. TNFa can also act in a positive feedback loop with IFNIs further amplifying this response. Thus, it would be very insightful to examine whether TNFa and CCL2 either individually or together are required for this positive regulation of the prime boost effect and plan to incorporate this great suggestion in our next studies.

Minor comments:

1. I suggest the author revise the title of each figure, some are too long (such as Fig. 5 and Fig. 7) In Fig. 6, title is missing.
2. In Fig. 5M, the IL-17A "+" should be superscript.
3. In Fig. 6, please standardize the gene names, like Il1b and Tnf.

We greatly appreciate the reviewer for noting these typos. We have corrected these mistakes and revised all long titles of the figures to be more concise.

16th Sep 2026

Dear Dr. Wong,

Thank you for submitting your revised manuscript, and please accept my apologies for the delay in getting back to you, which was due to annual leave among both the referees and members of the editorial team.

I have now received the reports from the three referees. As you will see below, referees #3 and #5 are satisfied with the revisions, while referee #4 raises some further concerns. We have discussed the reports within the editorial team and would like to invite you to submit a minor revision addressing referee #4's remaining points through additional discussion and appropriate referencing, where applicable. We do not consider additional experiments necessary at this stage.

Provided that these points and the editorial requests below are satisfactorily addressed, I should be able to accept the manuscript for publication.

In addition, please take into account the following editorial requests:

1/ Manuscript text
- Please use Track Changes for any new modifications to the text.
- We suggest revising the title, in particular to reword the phrase "uniquely needs IFN-Is" for clarity. For example, you might consider: "Spatiotemporal heterologous vaccination relies on type I interferons at priming to enhance mucosal immunity.
- Please revise the Abstract for clarity and readability, as several expressions are unnecessarily complex (e.g., "reverse sequential order", "pronouncedly more durable", and "allowing for synergism"). We also suggest simplifying the concluding sentence. Similarly, please consider shortening and simplifying the Results section headings, which are currently rather lengthy and complex.
- Methods:
o Animal experiments: Please provide the IACUC protocol/approval numbers for the animal experiments.
o Biosecurity: In view of the SARS-CoV-2 challenge experiments, please complete the relevant biosecurity and dual-use research questions in the Author Checklist and, where applicable, provide the required information on regulatory approval in the manuscript.
o Cell lines: Please provide information and complete the relevant sections of the Author Checklist regarding cell line authentication and mycoplasma testing.
o Antibodies: Please provide antibody dilutions.
o Please confirm whether there are any restrictions on the availability of the in-house NE/IVT reagents.
o Statistics: Please provide statements on sample-size determination, inclusion/exclusion criteria, and randomization, and complete the corresponding sections in the Author Checklist.
o Methods: Please carefully check the units reported throughout the Methods and correct them where necessary.
o The supplementary methods currently in the Appendix should be merged to the methods in the main manuscript file.
- Data Availability: Please clarify the following sentence: "The source data of this paper are collected in the following database record".
- Please merge the funding section with Acknowledgements, and remove the heading.
- Author contributions: Please remove this section from the manuscript and provide the CRediT (Contributor Role Taxonomy) terms in the submission system instead.

2/ Figures
- Please correct the nomenclature in the legend of the tables to Table EV1 and Table EV2. Please add a legend to Dataset EV1 with the title and a short description of the data presented.
- Appendix: Move the suppl. Methods to the manuscript text. We would encourage you to make your Appendix Figures into additional EV figures. If you choose to keep an Appendix, please accept the changes and upload the final appendix version as a PDF.
- Please provide the exact p values in the figures or legends of Fig. 1B-K; 2B, D, E, H; 3B-E, G-J, L-N; 4B-E, G, H, I, J, M, O, P, Q-S, U; 5B, C, F, G, H-J, K-N; 6A, B, C, D, E, G, I, J, K; 7B, F, G, H, J, K-N; EV1 B, F; EV2 B-G, I; EV4 D, E; EV5 A-D.

3/ Source Data:
We would encourage you to provide the FCS files underlying the flow cytometry data, where possible. These could, for example, be deposited in a public repository such as BioStudies.

4/ Thank you for providing the paper explained. Please include it in the main manuscript text, after the abstract.

5/ Synopsis: I introduced minor edits in your text, please let me know if you agree or amend as you see fit:
Sequential heterologous-route immunization using a rationally designed mucosal adjuvant enhances systemic and mucosal immunity compared with parenteral mRNA-only vaccination, highlighting the role of pre-established immunity in shaping

subsequent vaccination responses.
- Vaccine type and priming route imprint T cell Th bias, dictate mucosal TRM and sIgA induction, and skew systemic antibody class switching.
- Type I IFN induction at priming conditions responses to subsequent mucosal immunization, enhancing mucosal cellular and humoral immunity.
- Heterologous-route prime/boost immunization enhances the longevity (>3 months) of systemic and mucosal responses compared with homologous immunization.
- Robust mucosal immunity established through heterologous-route immunization can be boosted after a prolonged interval (>3 months) through either mucosal or parenteral routes.
- The same regimen can elicit distinct immune outcomes when administered in different chronological orders, highlighting the role of pre-existing immunity in shaping subsequent responses.

6/ As part of the EMBO Publications transparent editorial process initiative (see our Editorial at http://embomolmed.embopress.org/content/2/9/329), EMBO Molecular Medicine will publish online a Review Process File (RPF) to accompany accepted manuscripts.
This file will be published in conjunction with your paper and will include the anonymous referee reports, your point-by-point response and all pertinent correspondence relating to the manuscript. Let us know whether you agree with the publication of the RPF.
Please note that the Authors checklist will be published at the end of the RPF.

I look forward to receiving your revised manuscript.

With kind regards,

Lise Roth

Lise Roth, PhD
Senior Editor
EMBO Molecular Medicine

***** Reviewer's comments *****

Referee #3 (Comments on Novelty/Model System for Author):

The concept of systemic priming and mucosal boosting is not new, but the authors have done this in the context of a novel adjuvant system and provided a detailed analysis of the immune response.

Referee #3 (Remarks for Author):

The authors have adequately addressed my comments/concerns.

Referee #4 (Comments on Novelty/Model System for Author):

The amount of protein used to immunize mice is very high. Although the authors state that others also use these amounts, no references are provided to support this claim.

Referee #4 (Remarks for Author):

The authors have made some changes in response to the review. However, many other points are rebutted without references for the statements. Experiments that could have been conducted to address the comments (eg looking at cytokine production by nasal Trm cells) were not performed.

Referee #5 (Remarks for Author):

The authors have addressed all the issues I raised.

Responses to Reviewer Comments:

***** Reviewer's comments *****

Response: We are grateful to all of the reviewers for their comments and are very glad that our prior revisions have now sufficiently improved the manuscript.

Referee #3 (Comments on Novelty/Model System for Author):

The concept of systemic priming and mucosal boosting is not new, but the authors have done this in the context of a novel adjuvant system and provided a detailed analysis of the immune response.

Referee #3 (Remarks for Author):

The authors have adequately addressed my comments/concerns.

Referee #4 (Comments on Novelty/Model System for Author):

The amount of protein used to immunize mice is very high. Although the authors state that others also use these amounts, no references are provided to support this claim.

Referee #4 (Remarks for Author):

The authors have made some changes in response to the review. However, many other points are rebutted without references for the statements. Experiments that could have been conducted to address the comments (eg looking at cytokine production by nasal Trm cells) were not performed.

Response: We thank the reviewer for reviewing our manuscript again and agree adding some references would put antigen dose selection in better context. Thus, in addition to our previous additions in the last revision stating the different formats of WT S protein used in the field (S-2P vs. Hexapro) in the methods, we have now added more extensive discussion about the different formats of the WT SARS-CoV-2 spike protein (S-2P vs. the Hexapro (two proline substitutions vs. the more immunogenic six proline substitutions, respectively)) in the discussion section. Furthermore, we have added references to this discussion which cite studies using a similar range of dosing IN for the S-2P which we use herein, and the lower dose used IN for the more immunogenic Hexapro (lines 853-861 in markup version).

Referee #5 (Remarks for Author):

The authors have addressed all the issues I raised.

23rd Sep 2026

Dear Dr. Wong, Dear Pam,

Thank you for sending your revised files. I am pleased to inform you that your manuscript is accepted for publication and is now being sent to our publisher to be included in the next available issue of EMBO Molecular Medicine.

Your manuscript will be processed for publication by EMBO Press. It will be copy edited and you will receive page proofs prior to publication. Please note that you will be contacted by Springer Nature Author Services to complete licensing and payment information.

You may qualify for financial assistance for your publication charges - either via a Springer Nature fully open access agreement or an EMBO initiative. Check your eligibility: https://link.springer.com/journal/44321/how-to-publish-with-us

Should you be planning a Press Release on your article, please notify the production team via this form in order to coordinate publication and release dates: https://forms.cloud.microsoft/e/eH4A8JVP6g

If you have any questions, please do not hesitate to contact the Editorial Office. Thank you for your contribution to EMBO Molecular Medicine.

With kind regards,

Lise

Lise Roth, Ph.D
Senior Editor
EMBO Molecular Medicine

>>> Please note that it is EMBO Molecular Medicine policy for the transcript of the editorial process (containing referee reports and your response letter) to be published as an online supplement to each paper. If you do NOT want this, you will need to inform the Editorial Office via email immediately. More information is available here: https://link.springer.com/partners/embo-press/editorial-policies#Peer%20review

EMBO Press Author Checklist

Corresponding Author Name: Pamela Wong	
Journal Submitted to: EMBO Molecular Medicine	
Manuscript Number: EMM-2025-22617	

USEFUL LINKS FOR COMPLETING THIS FORM
The EMBO Journal - Author Guidelines
EMBO Reports - Author Guidelines
Molecular Systems Biology - Author Guidelines
EMBO Molecular Medicine - Author Guidelines

Reporting Checklist for Life Science Articles (updated January
This checklist is adapted from Materials Design Analysis Reporting (MDAR) Checklist for Authors. MDAR establishes a minimum set of requirements in transparent reporting in the life sciences (see Statement of Task: 10.31222/osf.io/9sm4x). Please follow the journal's guidelines in preparing your
Please note that a copy of this checklist will be published alongside your article.

Abridged guidelines for figures
1. Data
The data shown in figures should satisfy the following conditions:
- the data were obtained and processed according to the field's best practice and are presented to reflect the results of the experiments in an accurate and unbiased manner.
- ideally, figure panels should include only measurements that are directly comparable to each other and obtained with the same assay.
- plots include clearly labeled error bars for independent experiments and sample sizes. Unless justified, error bars should not be shown for technical
- if n<5, the individual data points from each experiment should be plotted. Any statistical test employed should be justified.
- Source Data should be included to report the data underlying figures according to the guidelines set out in the authorship guidelines on Data

2. Captions
Each figure caption should contain the following information, for each panel where they are relevant:
- a specification of the experimental system investigated (eg cell line, species name).
- the assay(s) and method(s) used to carry out the reported observations and measurements.
- an explicit mention of the biological and chemical entity(ies) that are being measured.
- an explicit mention of the biological and chemical entity(ies) that are altered/varied/perturbed in a controlled manner.
- the exact sample size (n) for each experimental group/condition, given as a number, not a range;
- a description of the sample collection allowing the reader to understand whether the samples represent technical or biological replicates (including how many animals, litters, cultures, etc.).
- a statement of how many times the experiment shown was independently replicated in the laboratory.
- definitions of statistical methods and measures:
  - common tests, such as t-test (please specify whether paired vs. unpaired), simple χ2 tests, Wilcoxon and Mann-Whitney tests, can be unambiguously identified by name only, but more complex techniques should be described in the methods section;
  - are tests one-sided or two-sided?
  - are there adjustments for multiple comparisons?
  - exact statistical test results, e.g., P values = x but not P values < x;
  - definition of 'center values' as median or average;
  - definition of error bars as s.d. or s.e.m.

Please complete ALL of the questions below.
Select "Not Applicable" only when the requested information is not relevant for your study.

Materials

Newly Created Materials	Information included in the manuscript?	In which section is the information available? (Reagents and Tools Table, Materials and Methods, Figures, Data Availability Section)
New materials and reagents need to be available; do any restrictions apply?	Not Applicable	new materials and reagents (NE/IVT) are available upon request

Antibodies	Information included in the manuscript?	In which section is the information available? (Reagents and Tools Table, Materials and Methods, Figures, Data Availability Section)
For **antibodies** provide the following information:<br>- Commercial antibodies: RRID (if possible) or supplier name, catalogue number and or/clone number<br>- Non-commercial: RRID or citation	Yes	Reagents and Tools Table, Table EV1

DNA and RNA sequences	Information included in the manuscript?	In which section is the information available? (Reagents and Tools Table, Materials and Methods, Figures, Data Availability Section)
**Short novel DNA or RNA including primers, probes**: provide the sequences.	Yes	Reagents and Tools Table, Table EV2

Cell materials	Information included in the manuscript?	In which section is the information available? (Reagents and Tools Table, Materials and Methods, Figures, Data Availability Section)
**Cell lines:** Provide species information, strain. Provide accession number in repository **OR** supplier name, catalog number, clone number, and/**OR** RRID.	Yes	Reagents and Tools Table
**Primary cultures:** Provide species, strain, sex of origin, genetic modification status.	Yes	Materials and Methods
Report if the cell lines were recently **authenticated** (e.g., by STR profiling) and tested for mycoplasma contamination.	Yes	Cell lines used were authenticated by ATCC (BEI Resources) along with mycoplasma testing prior to purchase, and strictly used at low passages.

Experimental animals	Information included in the manuscript?	In which section is the information available? (Reagents and Tools Table, Materials and Methods, Figures, Data Availability Section)
**Laboratory animals or Model organisms:** Provide species, strain, sex, age, genetic modification status. Provide accession number in repository **OR** supplier name, catalog number, clone number, **OR** RRID.	Yes	Reagents and Tools Table
**Animal observed in or captured from the field:** Provide species, sex, and age where possible.	Not Applicable	
Please detail **housing and husbandry conditions**.	Yes	Materials and Methods

Plants and microbes	Information included in the manuscript?	In which section is the information available? (Reagents and Tools Table, Materials and Methods, Figures, Data Availability Section)
**Plants:** provide species and strain, ecotype and cultivar where relevant, unique accession number if available, and source (including location for collected wild specimens).	Not Applicable	
**Microbes:** provide species and strain, unique accession number if available, and source.	Not Applicable	

Human research participants	Information included in the manuscript?	In which section is the information available? (Reagents and Tools Table, Materials and Methods, Figures, Data Availability Section)
If collected and within the bounds of privacy constraints report on age, sex and gender or ethnicity for all study participants.	Not Applicable	

Core facilities	Information included in the manuscript?	In which section is the information available? (Reagents and Tools Table, Materials and Methods, Figures, Data Availability Section)
If your work benefited from core facilities, was their service mentioned in the acknowledgments section?	Yes	Reagents and Tools Table, Acknowledgements

Design

Study protocol	Information included in the manuscript?	In which section is the information available? (Reagents and Tools Table, Materials and Methods, Figures, Data Availability Section)
If study protocol has been **pre-registered, provide DOI in the manuscript**. For clinical trials, provide the trial registration number **OR** cite DOI.	Not Applicable	
Report the **clinical trial registration number** (at ClinicalTrials.gov or equivalent), where applicable.	Not Applicable	

Laboratory protocol	Information included in the manuscript?	In which section is the information available? (Reagents and Tools Table, Materials and Methods, Figures, Data Availability Section)
Provide DOI OR other citation details if **external detailed step-by-step protocols** are available.	Not Applicable	

Experimental study design and statistics	Information included in the manuscript?	In which section is the information available? (Reagents and Tools Table, Materials and Methods, Figures, Data Availability Section)
Include a statement about **sample size** estimate even if no statistical methods were used.	Yes	Materials and Methods
Were any steps taken to minimize the effects of subjective bias when allocating animals/samples to treatment (e.g. **randomization procedure**)? If yes, have they been described?	Yes	Materials and Methods
Include a statement about **blinding** even if no blinding was done.	Yes	Materials and Methods
Describe **inclusion/exclusion criteria** if samples or animals were excluded from the analysis. Were the criteria pre-established?<br><br>If sample or data points were omitted from analysis, report if this was due to attrition or intentional exclusion and provide justification.	Yes	No data points were excluded from the analysis.
For every figure, are **statistical tests** justified as appropriate? Do the data meet the assumptions of the tests (e.g., normal distribution)? Describe any methods used to assess it. Is there an estimate of variation within each group of data? Is the variance similar between the groups that are being statistically compared?	Yes	Statistical tests were justified for each data set using assumptions of normality or lack therof based upon the type of measurements being examined. Statistics were determined by GraphadPrism which provides additional criteria on the appropriateness of the statistical test for the data type.

Sample definition and in-laboratory replication	Information included in the manuscript?	In which section is the information available? (Reagents and Tools Table, Materials and Methods, Figures, Data Availability Section)
In the figure legends: state number of times the experiment was **replicated** in laboratory.	Yes	
In the figure legends: define whether data describe **technical or biological replicates**.	Yes	

Ethics

Ethics	Information included in the manuscript?	In which section is the information available? (Reagents and Tools Table, Materials and Methods, Figures, Data Availability Section)
Studies involving **human participants**: State details of **authority granting ethics approval** (IRB or equivalent committee(s), provide reference number for approval.	Not Applicable	
Studies involving **human participants**: Include a statement confirming that **informed consent** was obtained from all subjects and that the experiments conformed to the principles set out in the WMA Declaration of Helsinki and the Department of Health and Human Services Belmont Report.	Not Applicable	
Studies involving **human participants:** For publication of **patient photos**, include a statement confirming that consent to publish was obtained.	Not Applicable	
Studies involving experimental **animals**: State details of **authority granting ethics approval** (IRB or equivalent committee(s), provide reference number for approval. Include a statement of compliance with ethical regulations.	Yes	Materials and Methods
Studies involving **specimen and field samples:** State if relevant **permits** obtained, provide details of authority approving study; if none were required, explain why.	Not Applicable	

Dual Use Research of Concern (DURC)	Information included in the manuscript?	In which section is the information available? (Reagents and Tools Table, Materials and Methods, Figures, Data Availability Section)
Could your study fall under dual use research restrictions? Please check biosecurity documents and list of **select agents and toxins** (CDC): https://www.selectagents.gov/sat/list.htm	Not Applicable	
If you used a select agent, is the security level of the lab appropriate and reported in the manuscript?	Not Applicable	
If a study is subject to dual use research of concern regulations, is the name of the **authority granting approval and reference number** for the regulatory approval provided in the manuscript?	Not Applicable	

Reporting

The MDAR framework recommends adoption of discipline-specific guidelines, established and endorsed through community initiatives. Journals have their own policy about requiring specific guidelines and recommendations to complement MDAR.

Adherence to community standards	Information included in the manuscript?	In which section is the information available? (Reagents and Tools Table, Materials and Methods, Figures, Data Availability Section)
State if relevant guidelines or checklists (e.g., **ICMJE, MIBBI, ARRIVE, PRISMA**) have been followed or provided.	Not Applicable	
For **tumor marker prognostic studies,** we recommend that you follow the **REMARK** reporting guidelines (see link list at top right). See author guidelines, under 'Reporting Guidelines'. Please confirm you have followed these guidelines.	Not Applicable	
For **phase II and III randomized controlled trials**, please refer to the **CONSORT** flow diagram (see link list at top right) and submit the CONSORT checklist (see link list at top right) with your submission. See author guidelines, under 'Reporting Guidelines'. Please confirm you have submitted this list.	Not Applicable	

Data Availability

Data availability	Information included in the manuscript?	In which section is the information available? (Reagents and Tools Table, Materials and Methods, Figures, Data Availability Section)
Have **primary datasets** been deposited according to the journal's guidelines (see 'Data Deposition' section) and the respective accession numbers provided in the Data Availability Section?	Not Applicable	
Were **human clinical and genomic datasets** deposited in a public access-controlled repository in accordance to ethical obligations to the patients and to the applicable consent agreement?	Not Applicable	
Are **computational models** that are central and integral to a study available without restrictions in a machine-readable form? Were the relevant accession numbers or links provided?	Not Applicable	
If publicly available data were reused, provide the respective **data citations in the reference list**.	Not Applicable	
